# Noncanonical role of astrocytic mitochondrial Cx43: suppressing IDH3α to sustain glycolytic homeostasis against depression

**DOI:** 10.1038/s41419-025-08309-1

**Published:** 2025-12-08

**Authors:** Junrui Ye, Hongyun Wang, Ye Peng, Shasha Wang, Ruifang Zheng, Yuqi Chen, Ruolan Yuan, Zhenzhen Wang, Xu Yan, Wenbin He, Gang Li, Hongshuo Sun, Zhongping Feng, Shifeng Chu, Zhao Zhang, Naihong Chen

**Affiliations:** 1 State Key Laboratory of Bioactive Substances and Functions of Natural Medicines, Institute of Materia Medica & Neuroscience Center, Chinese Academy of Medical Sciences and Peking Union Medical College, Beijing, China; 2 Xinjiang Institute of Materia Medica, Urumqi, China; 3 National International Joint Research Center for Molecular Chinese Medicine, Shanxi University of Chinese Medicine, Taiyuan, China; 4 Graduate school, Inner Mongolia Medical University, Hohhot, China; 5 Department of Physiology, Faculty of Medicine, University of Toronto, Toronto, ON Canada

**Keywords:** Astrocyte, Depression, Metabolic pathways

## Abstract

Depression remains a pervasive global health challenge, compounded by limited therapeutic efficacy that is partly attributed to incompletely understood metabolic underpinnings. In this study, we reveal a noncanonical mitochondrial function of astrocytic connexin 43 (Cx43) whereby it directly inhibits isocitrate dehydrogenase 3α (IDH3α), thereby sustaining glycolytic metabolism and lactate production essential for neuronal excitability. Astrocyte-specific deletion of Cx43 in the prelimbic cortex (PrL) recapitulates hallmark depressive phenotypes, characterized by reduced lactate synthesis, diminished neuronal excitability, and depressive-like behaviors. Loss of Cx43 enhances IDH3α activity, prompting a metabolic reprogramming from aerobic glycolysis toward oxidative phosphorylation (OXPHOS) driven by glutamine-fueled anaplerosis, resulting in suppressed glucose uptake and decreased lactate output. This metabolic impairment restricts astrocytic lactate supply, depriving neurons of a critical energetic substrate. Importantly, this reprogramming occurs independently of gap junction intercellular communication, as demonstrated using a channel function-deficient dominant-negative Cx43 mutant. Restoration of mitochondrial Cx43 in astrocytes rescues neuronal excitability and ameliorates depressive-like phenotypes. Collectively, our findings identify mitochondrial Cx43 as a vital regulator of IDH3α activity, essential for astrocyte-neuron metabolic coupling, and highlight a promising target for therapeutic intervention in depression.

## Introduction

Depression remains a global health burden with limited treatment efficacy, partly due to incomplete understanding of its metabolic underpinnings [1, 2]. Mounting evidence underscores the pivotal role of the prefrontal cortex (PFC) in the pathophysiology of depression, attributed to its high glycolytic activity, which generates sufficient lactate to support neuronal excitability [3, 4]. The reduction of glycolysis and lactate levels in the PFC is a common metabolic hallmark observed in both clinical depression and animal models [5–8]. However, the available antidepressants mainly targeted on the monoamine systems [9], leaving a critical therapeutic gap in depression management.

Glycolysis is the primary metabolic pathway in astrocytes [10, 11], producing lactate that is transported extracellularly via monocarboxylate transporters (MCT) [12]. This lactate is subsequently taken up by neurons and converted into pyruvate, entering the tricarboxylic acid (TCA) cycle in a process known as the lactate shuttle [13, 14]. Lactate, the byproduct of glycolysis, exerts antidepressant effects by enhancing stress resistance and promoting neurogenesis [15–17]. Under resting conditions, glucose undergoes aerobic glycolysis, generating two molecules of lactate [11]. Deficiency of lactate dehydrogenase A (LDHA), the rate-limiting glycolytic enzyme in astrocytes, leads to reduced neuronal excitability and the emergence of depressive-like behaviors [6]. Upon neuronal excitation, elevated extracellular glutamate stimulates glycolysis through membrane-associated phosphoglycerate kinase (PGK), supplying localized ATP to Na-K ATPase and thereby enabling astrocytes to meet the increased energetic and neurotransmitter regulatory demands [13, 18]. Thus, astrocytic glycolytic function is critical for maintaining and responding to neuronal energy metabolism and excitability, playing a pivotal role in the modulation of depressive symptoms.

Clinical investigations have revealed a marked reduction in the number of astrocytes within the PFC of patients with depression who died by suicide, accompanied by decreased levels of the gap junction protein connexin 43 (Cx43) [19, 20]. Our previous studies further demonstrated that in the chronic unpredictable mild stress (CUMS) model of depression, Cx43 expression is selectively diminished in the PFC, even in the absence of changes in astrocyte density [21]. As the most abundantly expressed gap junction protein in the brain, Cx43 facilitates intercellular transfer of small molecules including lactate, glucose, and ATP via gap junction channels [19, 22]. Notably, the reduced Cx43 protein levels observed in depression models are concomitant with impaired gap junction-mediated intercellular communication [21–23]. Disruption of the Cx43-mediated network, whether induced by stress or genetic manipulation, compromises the distribution of energy substrates throughout this network, thereby detrimentally impacting neuronal function [24, 25]. Moreover, Cx43 also exists in both monomeric and polymeric forms and localizes to astrocytic mitochondria, where it may contribute to the maintenance of mitochondrial homeostasis [26–29]. Nonetheless, it remains unclear whether Cx43 downregulation directly causes glycolytic dysfunction in depression.

Using a multidisciplinary approach encompassing neuroimaging, electrophysiological, biochemical, and behavioral assays, we demonstrate a critical role for Cx43 in depression via the reprogramming of astrocytic central carbon metabolism. Through the use of Cx43 knockout models, we reveal that Cx43 regulates metabolism independently of its canonical gap junction function. Furthermore, we identify a previously unrecognized interaction between Cx43 and mitochondrial isocitrate dehydrogenase 3α (IDH3α), which modulates oxidative phosphorylation (OXPHOS) and ATP synthesis, thereby influencing glucose uptake and glycolysis. Crucially, depressive-like behaviors induced by Cx43 deficiency can be ameliorated by mitochondrial overexpression of Cx43, indicating that mitochondrial Cx43 in astrocytes represents a viable target for antidepressant development. Our findings uncover a novel noncanonical mechanism whereby mitochondrial Cx43 governs astrocytic metabolism and propose a therapeutic strategy aimed at astrocytic metabolic reprogramming to mitigate depressive symptoms.

## Results

### Astrocytic Cx43 deletion disrupts lactate homeostasis and impairs neuronal excitability

Reduced glycolysis in the PFC constitutes a significant pathological feature of depression [6, 11]. To observe the potential relationship between Cx43 and glycolysis in depression, we established a chronic social defeat stress (CSDS) mice model (Fig. S1A), as detailed in the supplementary methods. CSDS-exposed mice exhibited pronounced depressive-like behaviors (Fig. S1B and C), concomitant with reduced body weight gain and daily food intake compared to control (Fig. S1E and F). Consistent with our previous findings [21], immunoblotting analysis revealed region-specific Cx43 downregulation in the PrL and hippocampus of mice subjected to CSDS, while no significant differences were detected in the striatum and hypothalamus (Fig. S1D). Furthermore, Seahorse analysis of isolated PrL demonstrated a significant decline in glycolytic capacity in the CSDS group, accompanied by a notable reduction in lactate (Fig. S1G and H). These findings suggested that Cx43 deficiency might involve into the compromised glycolysis in the PrL.

**Fig. 1:**
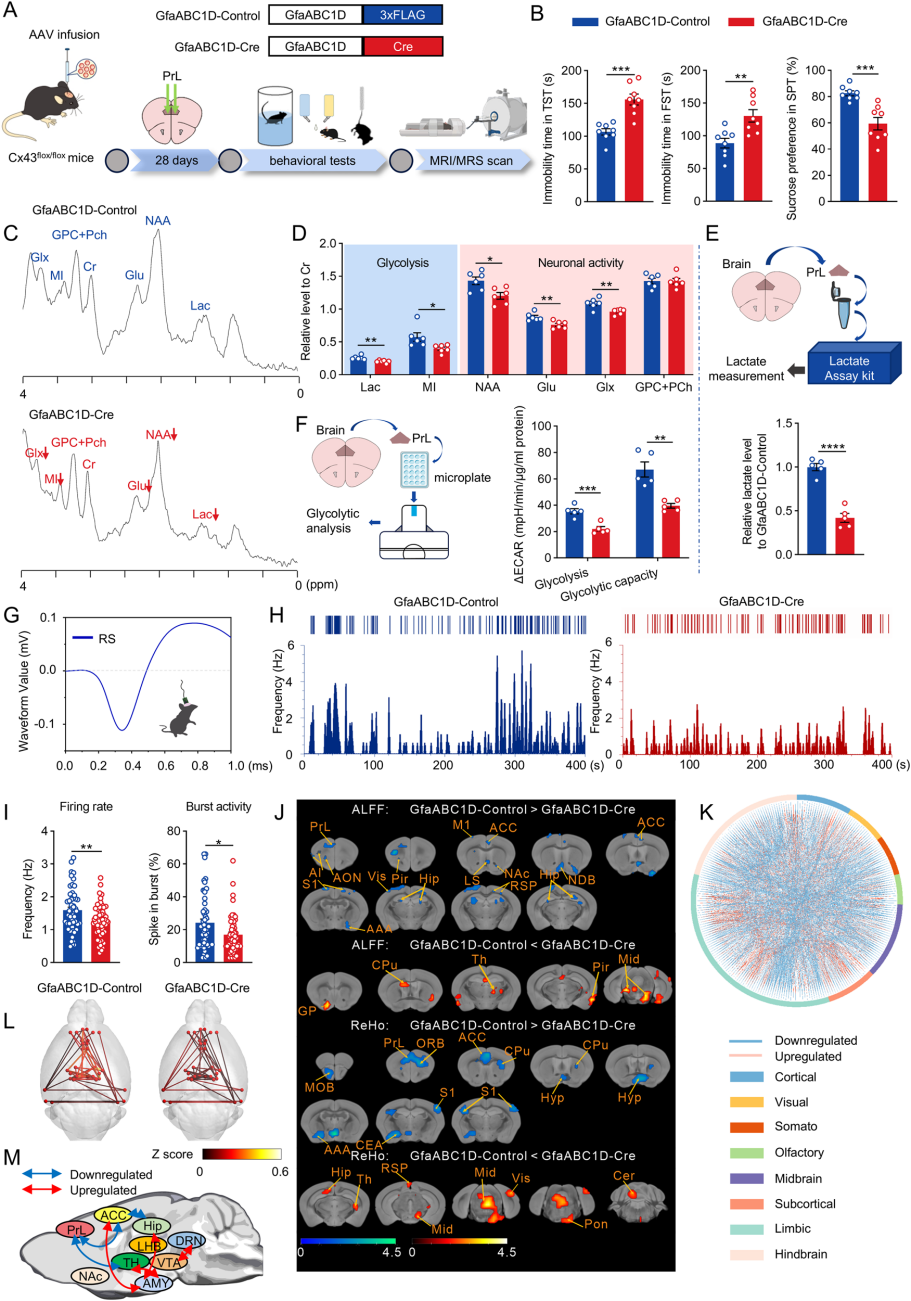
Astrocytic Cx43 deletion compromises lactate homeostasis and neuronal excitability. **A** The diagram of experimental settings. **B** Immobility time in the TST and FST, and sucrose preference in the SPT. n = 8. **C** Representative images of ^1^H MRS spectra acquired from the PrL of GfaABC1D-Control and GfaABC1D-Cre mice. **D** The relative metabolite concentrations in the PrL of GfaABC1D-Control and GfaABC1D-Cre mice. n = 6. **E** The level of lactate in the PrL detected by the lactate assay kit. n = 5. **F** Quantitative glycolytic capacity of PrL by Seahorse XF analysis. n = 5. **G** The waveform of representative putative pyramidal neuron (RS) units. **H** The representative firing rate histogram of pyramidal neurons in the PrL. **I** Firing rate and percent of spikes in bursting mode recorded from the PrL in the GfaABC1D-Control and GfaABC1D-Cre mice. n = 53 from 4 mice per group. **J** Statistical maps of voxel *t* values of ALFF and ReHo comparisons of two chosen groups. The color bars were used to signify the *t*-value of the group analysis (the color is brighter; the *t* value is higher). **K** Connectome map indicating significant changes (*P* < 0.05) in functional connectivity: outer circle lines represent brain regions of the respective color as indicated in the legend. **L** Functional network alterations are depicted in this schematic, showing the varying degrees of functional connectivity between node pairs through lines of thicker widths corresponding to higher Z-score values. **M** Representative brain volumes of resting-state functional connectivity in the limbic system. Arrows indicate directionality of connections from the indicated seed regions; arrow color reflects type of connection change based on type-of-connection legend. Two-tailed Student’s *t*-test or Welch’s *t*-test were performed between two groups; Mann–Whitney test in (**I**) (Burst activity). All data represent the mean ± SEM. ^*^P < 0.05, ^**^P < 0.01, ^***^P < 0.001, ^****^P < 0.0001.

As well known, Cx43 is preferentially expressed in astrocytes within the brain [19, 22]. However, data from the protein atlas also indicate the presence of Cx43 in microglia (refer to https://www.proteinatlas.org). To delineate the distinct contributions of Cx43 reduction in astrocytes versus microglia in depressive-like behaviors, we generated conditional knockout mice with targeted deletion of Cx43 in either astrocytes (via AAV2/9-GfaABC1D-Cre, Fig. 1A and S2A–D) or microglia (via AAV-MG1.2-Cx3cr1-Cre, Fig. S2E–I) within the PrL of Cx43^flox/flox^ mice. The behavioral tests demonstrated that deletion of microglial Cx43 did not elicit depressive-like behaviors, as assessed by the tail suspension test (TST), forced swimming test (FST), and sucrose preference test (SPT) (Fig. S2J). Conversely, the deletion of astrocytic Cx43 resulted in increased immobility time in the TST and FST and reduced sucrose preference in the SPT, all indicative of depressive-like phenotypes (Fig. 1B).

The deficiency of Cx43 also resulted in a reactive phenotype in astrocytes, characterized by increased branching in Sholl analysis (Fig. S3A and B), elevated reactive astrocytic markers at the mRNA level (Fig. S3C) and enhanced secretion of inflammatory cytokines, such as granulocyte-macrophage colony stimulating factor (GM-CSF) and granulocyte colony stimulating factor (G-CSF) (Fig. S3D and E).

Based on the behavioral and phenotype changes induced by astrocytic Cx43 deletion, we assessed the lactate levels in the PrL using in vivo ^1^H magnetic resonance spectroscopy (MRS). This analysis revealed significant reductions in the ratios of lactate/creatine (Cr) in GfaABC1D-Cre mice compared to those in GfaABC1D-Control mice (Fig. 1C and D). The reduction in lactate level was further corroborated by direct quantification of isolated PrL samples (Fig. 1E), both of which revealed a remarkable reduction in lactate induced by the loss of astrocytic Cx43, potentially due to decreased glycolytic activity. To confirm our suspicions, we measured the extracellular acidification rate (ECAR) in the isolated PrL samples. The ECAR of GfaABC1D-Cre mice, following both glucose and oligomycin injections, was significantly lower than that in GfaABC1D-Control mice, indicating a reduction in basal glycolysis and glycolytic capacity resulting from the knockout of astrocytic Cx43 (Fig. 1F).

In addition, we also observed the decreased *N*-acetylaspartate (NAA), myo-inositol (MI), glutamate (Glu) and glutamate + glutamine (Glx) levels (Fig. 1D), which implied that neuronal excitability was compromised following the loss of astrocytic Cx43. This speculation was further supported by electrophysiological recordings from pyramidal neurons in the PrL of GfaABC1D-Cre mice, which exhibited reduced firing frequency and spike burst activity compared to those in GfaABC1D-Control mice (Fig. 1G–I).

Moreover, functional magnetic resonance imaging (fMRI) was employed to extend our investigation to brain-wide activity. The low frequency fluctuation (ALFF) demonstrated a notable decline in several regions of the limbic system, including the PrL, anterior cingulate cortex (ACC), agranular insular area (AI), anterior olfactory nucleus (AON), diagonal band nucleus (NDB), lateral septal nucleus (LS), primary motor cortex (M1), piriform cortex (Pir), anterior amygdalar area (AAA), hippocampus (Hip), retrosplenial cortex (RSP) and nucleus accumbens (NAc). The assessment of regional homogeneity (ReHo) revealed a marked reduction in the limbic system areas, including the PrL, olfactory bulb (MOB), orbital cortex (ORB), ACC, caudoputamen (CPu), hypothalamus (Hyp), AAA, primary sensory cortex (S1), and central amygdalar nucleus (CEA). Conversely, an elevation in ReHo was discernible in regions encompassing the Hip, thalamus (Th), midbrain (Mid), RSP, visual cortex (Vis), pons (Pon), and cerebellum (Cer), suggesting a disruption in local synchronization (Fig. 1J).

To observe the impact of astrocytic Cx43 in PrL on overall brain function, we segmented the brain into 258 regions of interest across eight sections: cortical, visual, somatic, olfactory, midbrain, subcortical, limbic, and hindbrain regions. Intergroup comparisons of pairwise correlation matrices across the entire brain revealed notable alterations in brain-wide resting-state functional connectivity (Fig. 1K). The default mode network (DMN) and limbic system to which the PFC belongs are intricately tied to activities such as emotional regulation and motivational decision-making [30, 31]. We delved deeper into the alterations in functional connectivity within the limbic system and the DMN. Cx43 knockout in the PrL notably diminished the functional connections between the PrL and Th; the PrL and ACC; the ACC and Hip, while augmenting the connections between the ACC and amygdala (AMY); the Th and AMY, as well as the ventral tegmental area (VTA) and dorsal raphe nucleus (DRN) (Fig. 1L and M). These findings imply that the loss of astrocytic Cx43 in the PrL elicits aberrations in both the intensity and synchronization of neural activities, giving rise to extensive connectivity irregularities indicative of depressive-like behaviors, particularly within the neural network associated with depression.

### Deletion of Cx43 reduces astrocytic lactate production by suppressing glucose metabolic flux

**Fig. 2:**
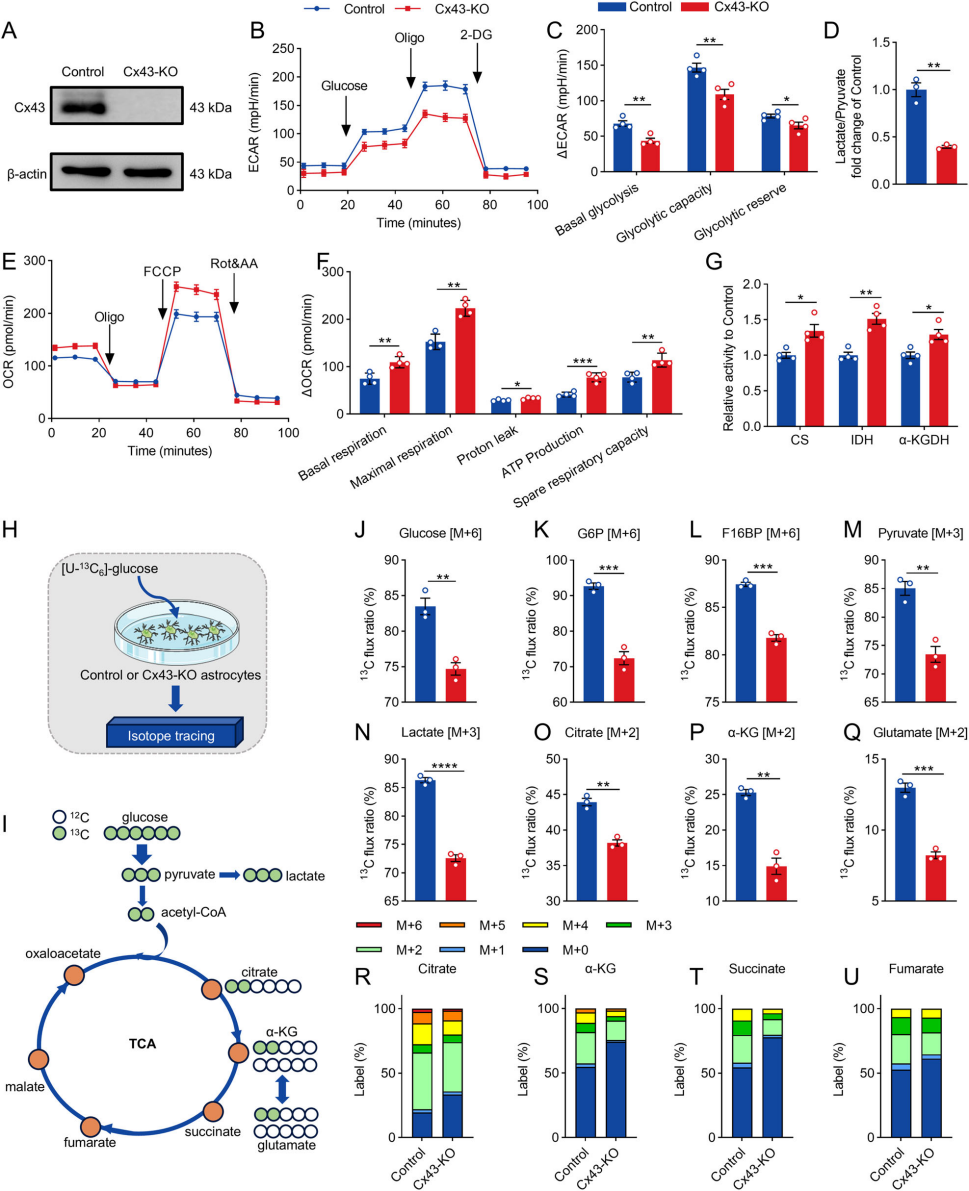
Astrocytic Cx43 deletion impairs glucose metabolism in astrocytes. **A** Western blots showed Cx43 expression in Control and Cx43-KO astrocytes. **B** The real-time changes of ECAR traces in Control and Cx43-KO astrocytes. **C** Quantification of basal glycolysis, glycolytic capacity and glycolytic reserve. n = 4. **D** The relative ratio of lactate/pyruvate in the quantification of the metabolic flux assay. n = 3. **E** The real-time changes of OCR traces in Control and Cx43-KO astrocytes. **F** Quantification of basal respiration, maximal respiration, proton leak, ATP production and spare respiratory capacity. n = 4. **G** The enzyme activities of citrate synthase (CS), isocitrate dehydrogenase (IDH), and α-ketoglutarate dehydrogenase (α-KGDH) in Control and Cx43-KO astrocytes. n = 4. **H**, **I** Control and Cx43-KO astrocytes were cultured with 2 g/L [U-^13^C_6_]-D-glucose for 24 h. **J**–**N** The ^13^C-labeled flux ratio of metabolites in the glycolysis, including glucose, glucose-6-phosphate (G6P), fructose-1,6-bisphosphate (F16BP), pyruvate, and lactate. n = 3. **O**–**U** The ^13^C-labeled flux ratio and the mass isotopomer distributions of metabolites, including glutamate, α-ketoglutarate (α-KG), citrate, succinate, and fumarate. n = 3. Two-tailed Student’s *t* test was performed between two groups. All data represent the mean ± SEM. ^*^P < 0.05, ^**^P < 0.01, ^***^P < 0.001, ^****^P < 0.0001.

Under physiological conditions, astrocytes exhibit a heightened glycolytic activity, which plays a crucial role in maintaining lactate homeostasis within the brain [4, 11, 32]. In the glycolytic pathway, a single glucose molecule is typically metabolized into two lactate molecules, representing the primary route for lactate production [11]. To observe the impact of Cx43 on glycolytic metabolism, we used an in vitro Cx43-KO astrocyte model (Fig. 2A) and performed Seahorse XF analysis. Our findings revealed that upon sequential addition of glucose and oligomycin, the ECAR in the Cx43-KO astrocytes was markedly reduced as compared to those in control astrocytes (Fig. 2B). This indicated that Cx43 deletion substantially decreased basal and glycolytic capacities in astrocytes (Fig. 2C).

Lactate, as a product of glycolysis, is transformed from pyruvate by LDHA. Analysis of the lactate-to-pyruvate ratio demonstrated a significant decrease in Cx43-KO astrocytes (Fig. 2D), suggesting an inhibition of pyruvate-to-lactate conversion. The residual pyruvate can be converted to acetyl-CoA in the mitochondria, which subsequently enters the TCA cycle [33, 34]. Therefore, we investigated the effect of Cx43 deletion on mitochondrial respiration. Our results revealed a significant increase in the oxygen consumption rate (OCR), leading to enhanced basal and maximal mitochondrial respiration, proton leak, spare respiratory capacity, and elevated ATP production upon Cx43 deletion (Fig. 2E and F). This upregulation of the TCA cycle was further supported by the increased activities of rate-limiting enzymes, including citrate synthase (CS), isocitrate dehydrogenase (IDH), and α-ketoglutarate dehydrogenase (α-KGDH) (Fig. 2G). These findings indicated that there is a metabolic reprogramming from glycolysis to OXPHOS in Cx43-KO astrocytes.

To elucidate whether a reduced lactate-to-pyruvate conversion drove the elevated TCA cycle activity, we used [U-^13^C_6_]-glucose to trace changes in glucose metabolites (Fig. 2H and I). Our analysis demonstrated a significant reduction in ^13^C-labeled glucose [M + 6] levels in astrocytes following Cx43 deletion (Fig. 2J). Concordantly, the levels of glycolytic intermediates, including glucose-6-phosphate (G6P) [M + 6] and fructose-1,6-bisphosphate (F16BP) [M + 6], were markedly reduced (Fig. 2K and L). This contributed to reduced production of pyruvate [M + 3] and lactate [M + 3] (Fig. 2M and N), indicating a substantial downregulation of glycolytic flux.

Upon decarboxylation by pyruvate dehydrogenase, pyruvate [M + 3] released one molecule of CO_2_, forming acetyl-CoA [M + 2], subsequently entering the TCA cycle. We observed significant declines in TCA cycle intermediates driven by glucose metabolism, including citrate [M + 2] (Fig. 2O and R) and α-ketoglutarate (α-KG) [M + 2] (Fig. 2P), accompanied by altered mass isotopomer distributions of α-KG, succinate, and fumarate in Cx43-deficient astrocytes (Fig. 2S–U). We also observed a marked decrease in glutamate [M + 2] levels (Fig. 2Q), suggesting that Cx43 deletion impaired glutamate synthesis. These findings demonstrate that Cx43 loss in astrocytes leads to a comprehensive suppression of glucose metabolism. However, the proportion of unlabeled TCA cycle intermediates increased significantly, suggesting the involvement of glucose-independent energy substrates in the enhanced OXPHOS observed upon Cx43 deletion.

### Glutamine-fueled anaplerosis supports the enhanced OXPHOS in Cx43-KO astrocytes

**Fig. 3:**
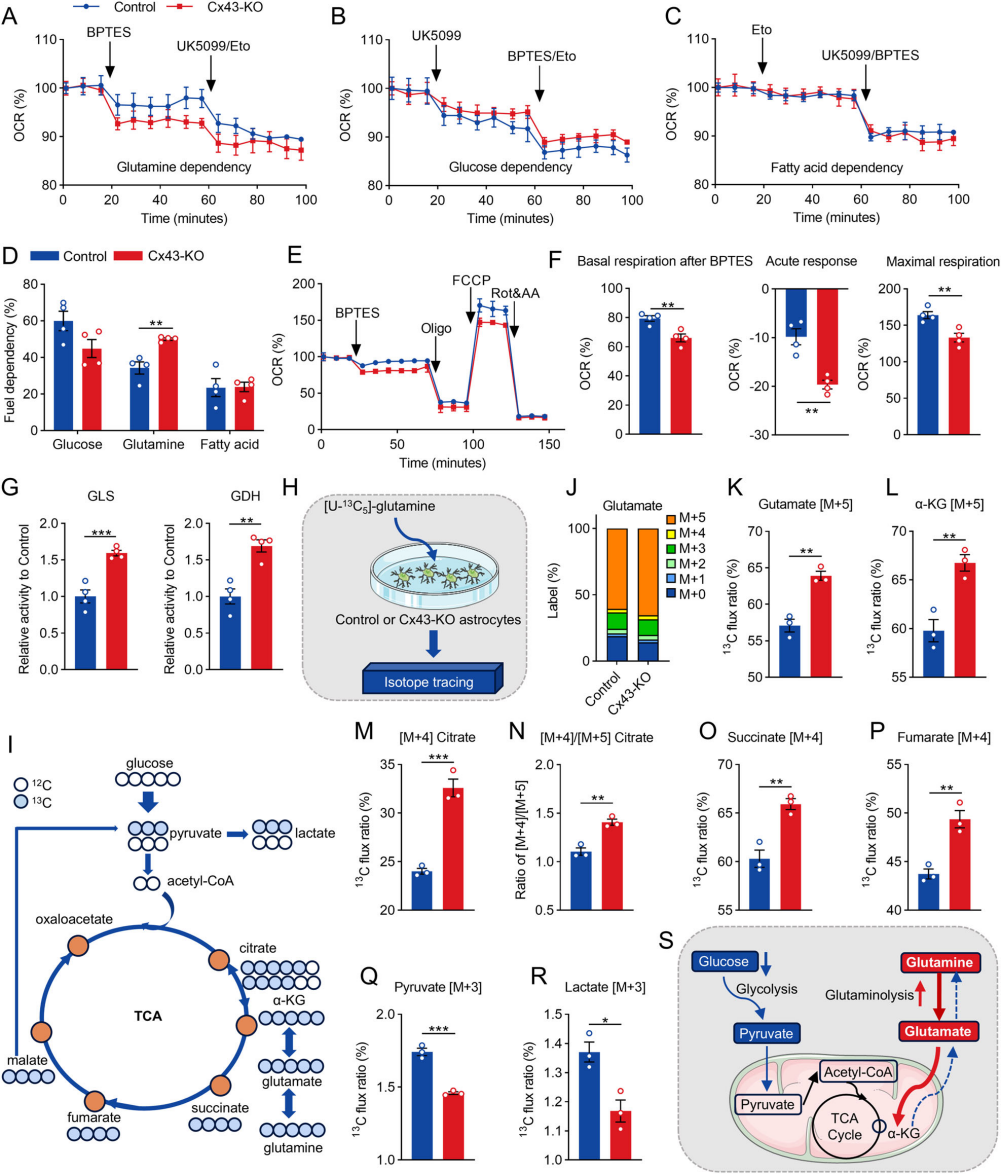
Astrocytic Cx43 deletion elevates OXPHOS driven by glutamine-fueled anaplerosis. The fuel flexibility assay determined by real-time changes in the OCR trace, reveals the dependency of astrocytes on glutamine (**A**), glucose (**B**) and fatty acids (**C**) for OXPHOS. **D** The dependency of astrocytes on glucose, glutamine, or fatty acids for OXPHOS. n = 4. **E** The real-time changes of OCR trace in the glutamine oxidation stress test. **F** Quantification of basal respiration after BPTES, acute response, and maximal respiration. n = 4. **G** The enzymatic activities of glutaminase (GLS) and glutamate dehydrogenase (GDH). n = 4. **H**, **I** Control and Cx43-KO astrocytes were cultured with 4 mM [U-^13^C_5_]-glutamine for 12 h. **J**, **K** The ^13^C-labeled flux ratio and the mass isotopomer distributions of glutamate. **L**–**P** The ^13^C-labeled flux ratio of metabolites of metabolites in the TCA cycle, including α-ketoglutarate (α-KG), citrate, succinate, and fumarate. n = 3. **Q**, **R** The [M + 3] ^13^C-labeled flux ratio of metabolites in the glycolysis, including pyruvate and lactate. n = 3. **S** Schematic diagram depicting the changes of glucose and glutamine dependency in OXPHOS. Two-tailed Student’s *t* test was performed between two groups. All data represent the mean ± SEM. ^*^P < 0.05, ^**^P < 0.01, ^***^P < 0.001.

It is widely recognized that alternative substances can serve as energy substrates in the TCA cycle, highlighting the metabolic flexibility that enables cells to adapt to various stresses [33, 34]. Our findings revealed an intriguing metabolic paradox: despite Cx43 deletion leading to diminished glucose metabolic flux, astrocytes exhibited a marked increase in OCR value (Fig. 2), strongly suggesting the involvement of alternative substrates in mitochondrial respiration. Therefore, we employed an energy substrate preference assay to examine the alterations in substrate utilization patterns in Cx43-KO astrocytes. UK-5099 (a mitochondrial pyruvate carrier inhibitor), BPTES (a glutaminase inhibitor), and etomoxir (Eto, a carnitine palmitoyl-transferase 1 A inhibitor) were employed to assess the substrate dependency of oxidation in astrocytes. Our findings revealed a striking substrate-specific effect in Cx43-KO astrocytes. Based on the response of OCR values to various substrate oxidation inhibitors, it was observed that Cx43-KO significantly enhances the dependency on glutamine oxidation (Fig. 3A and D), while no significant differences were noted in their dependency on glucose and fatty acid oxidation (Fig. 3B–D). Moreover, inhibition of glutamine utilization led to a more pronounced decrease in the OCR of Cx43-KO astrocytes (Fig. 3E). A comprehensive analysis of mitochondrial respiratory function revealed significant reductions in basal respiration and maximal respiratory capacity, with a heightened acute response observed in Cx43-KO astrocytes treated with BPTES (Fig. 3F). These findings collectively indicated that enhanced glutamine utilization served as an alternative energy substrate in Cx43-KO astrocytes.

Glutamine can be enzymatically converted to α-KG, a key intermediate in the TCA cycle, through the sequential actions of glutaminase (GLS) and glutamate dehydrogenase (GDH). We observed significantly enhanced activities of both GLS and GDH in Cx43-KO astrocytes (Fig. 3G), indicating an increased capacity for glutamine utilization. To trace the changes in glutamine metabolites, we cultured astrocytes with [U-^13^C_5_]-glutamine and performed a comprehensive metabolic flux analysis (Fig. 3H and I). Our findings revealed a significant increase in glutamate [M + 5] and α-KG [M + 5] levels following Cx43 knockout (Fig. 3J–L), indicating enhanced glutaminolysis. To distinguish between oxidative decarboxylation and reductive carboxylation of TCA cycle intermediates derived from α-KG [M + 5], we examined the citrate [M + 4] and [M + 5] levels. Upon entry into the TCA cycle via α-KG, glutamine-derived α-KG undergoes oxidative decarboxylation to succinyl-CoA to generate citrate [M + 4] through the forward TCA cycle. Alternatively, α-KG can undergo reductive carboxylation via backflux to produce citrate [M + 5] [35]. Our results demonstrated a marked increase in the [M + 4]/[M + 5] citrate ratio accompanied by elevated citrate [M + 4] levels (Fig. 3M and N). Furthermore, we observed significant increases in downstream metabolites succinate [M + 4] and fumarate [M + 4] in the forward TCA cycle (Fig. 3O and P), suggesting enhanced oxidative decarboxylation of α-KG.

Given that citrate levels are also influenced by acetyl-CoA, we investigated the flux through malic enzyme (ME), which catalyzes the conversion of malate to pyruvate, linking glutamine to glucose metabolism. ME relative flux serves as an indicator of the connection between glycolysis and the TCA cycle [36]. Notably, Cx43 knockout resulted in decreased levels of pyruvate [M + 3] and lactate [M + 3] (Fig. 3Q and R), signifying a reduced ME relative flux and further supporting the dysregulation of glucose metabolism in these cells. Collectively, Cx43-KO astrocytes exhibit pronounced reliance on glutamine within the TCA cycle instead of glucose utilization (Fig. 3S).

### Astrocytic metabolic reprogramming induced by Cx43 loss is independent of its canonical function

It is well established that Cx43 preferentially assembles into hexameric structures, facilitating the formation of gap junctions that enable the transfer of energetic metabolites within the astrocytic network, a process critical for maintaining brain homeostasis [19, 37]. However, whether the metabolic reprogramming was mediated through its role in gap junctions remained unclear.

**Fig. 4:**
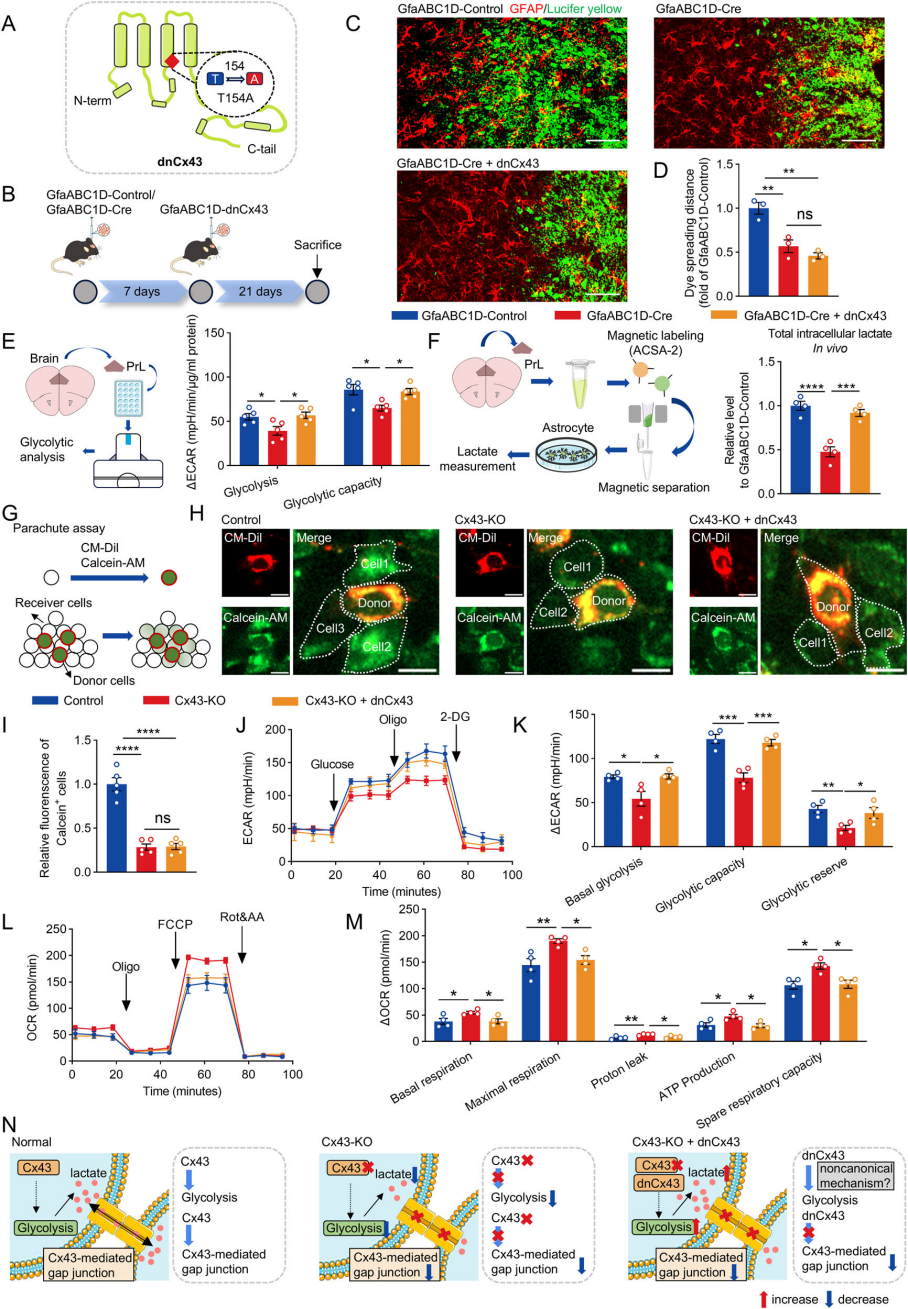
Cx43 modulates astrocytic metabolism independently of its gap junction function. **A** The schematic representation of the dnCx43 mutation. **B** Temporal flowchart of the model construction. **C** Representative images of the PrL from the Lucifer yellow diffusion assay. Scale bar, 50 μm. **D** Quantification of Lucifer yellow diffusion distance among astrocytes. n = 3. **E** Quantitative glycolytic capacity of PrL by Seahorse XF analysis. n = 5. **F** The schematic representation of the experimental procedure for the isolation of astrocytes from adult mice using magnetic bead sorting and the total intracellular lactate level in the isolated astrocytes in the PrL. n = 4. **G** Schematic of the parachute assay. **H** Representative images from the parachute assay. Scale bar, 20 μm. **I** Quantification of relative fluorescence intensity in Calcein-positive cells from the parachute assay. n = 5. **J** The real-time changes of ECAR traces in astrocytes. **K** Quantification of basal glycolysis, glycolytic capacity and glycolytic reserve. n = 4. **L** The real-time changes of OCR traces in astrocytes. **M** Quantification of basal respiration, maximal respiration, proton leak, ATP production and spare respiratory capacity. n = 4. **N** Comparative mechanistic diagram of dnCx43 and Cx43-KO models. One-way ANOVA followed by Dunnett’s multiple comparisons test in multiple groups. All data represent the mean ± SEM. ^*^P < 0.05, ^**^P < 0.01, ^***^P < 0.001, ^****^P < 0^.^0001. ns no significant difference.

To elucidate this issue, we injected an AAV-vector expressing a dominant-negative mutant of Cx43 (dnCx43) into the PrL, introducing a mutation at position 154, where threonine is substituted with alanine (Fig. 4A). This mutation selectively inhibiting Cx43-mediated gap junction function by disrupting conductivity, while permitting the formation of non-functional gap junction adhesion structures [38]. This dnCx43 expression also offers the advantage of minimally disrupting non-channel-mediated activities [24, 39].

To determine whether Cx43 regulates lactate production independently of its gap junction function, we stereotaxically injected AAV2/9-GfaABC1D-Cre in the PrL, followed seven days later by AAV2/9-GfaABC1D-dnCx43 (Fig. 4B). Quantitative immunoblotting showed that dnCx43 expression restored total Cx43 protein levels in the PrL (Fig. S4A and B). Pure mitochondria were isolated from magnetically sorted astrocytes [40] and immunoblotting confirmed the presence of both endogenous Cx43 and exogenous dnCx43 in astrocyte mitochondria (Fig. S4C–F). Lucifer yellow dye diffusion combined with immunofluorescence staining showed that astrocyte-specific Cx43 deletion substantially reduced dye diffusion among astrocytes, consistent with impaired gap-junctional coupling, and that expression of channel function-deficient dnCx43 failed to rescue this deficit (Fig. 4C and D). Seahorse analysis indicated that dnCx43 expression enhanced glycolytic function in the PrL (Fig. 4E), implying that Cx43 regulates lactate production is independent of its gap junctional channel activity. To eliminate potential confounding effects arising from intercellular lactate transfer, magnetic bead separation was employed to isolate astrocytes from the PrL, after which we quantified the total intracellular lactate levels. Remarkably, overexpression of dnCx43 restored the decrease in lactate levels induced by Cx43 knockout (Fig. 4F).

To further corroborate these findings, we cultured astrocytes in vitro and transfected them with a vector encoding dnCx43. Gap junction channel function was assessed using the parachute assay [41, 42] (Fig. 4G). Consistent with in vivo observations, Cx43 knockout markedly impaired dye spread via astrocytic networks, and dnCx43 overexpression did not restore gap‑junctional coupling (Fig. 4H and I). Importantly, Seahorse metabolic flux analysis revealed that expression of dnCx43 in Cx43‑deficient astrocytes reinstated metabolic reprogramming, characterized by enhanced glycolysis and concomitant suppression of OXPHOS (Fig. 4J–M). Besides gap junction channels, the monocarboxylate transporters MCT1 and MCT4 are principal mediators of lactate transport at the astrocyte membrane [10]. To assess their contribution to the observed metabolic phenotype, we knocked down MCT1 and MCT4 (Fig. S5A and B). Downregulation of either transporter had no effect on glycolytic flux or OXPHOS (Fig. S5C–J), indicating that MCT1 and MCT4 do not mediate the metabolic reprogramming described above.

Collectively, these findings indicate that the overexpression of dnCx43, which lacks gap junction function, restores metabolic reprogramming in Cx43-KO astrocytes. This suggests that the regulatory role of Cx43 in astrocytic metabolism operates independently of its canonical functions as a gap junction protein (Fig. 4N).

### Cx43–IDH3α interaction maintains glucose uptake and aerobic glycolysis

**Fig. 5:**
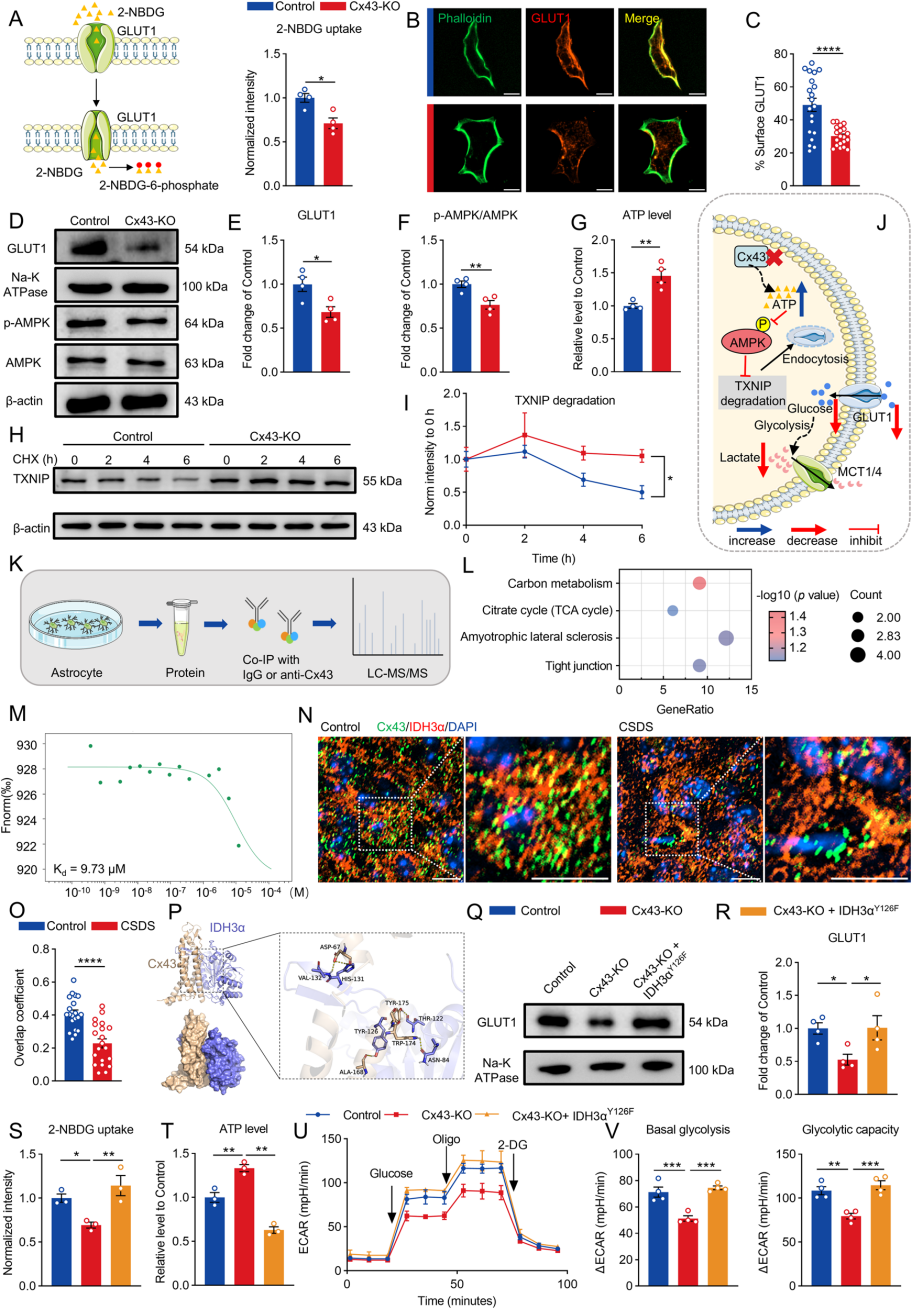
Cx43 regulates glucose uptake by interacting with IDH3α through the AMPK/TXNIP/GLUT1 pathway. **A** Glucose uptake ability assessed by 2-NBDG uptake. n = 4. **B**, **C** Representative images of immunofluorescence staining and quantification of cell surface GLUT1 in Control and Cx43-KO astrocytes. Scale bar = 20 μm. n = 20 cells. **D** Western blot analysis of membrane GLUT1, p-AMPK, AMPK, Na-K ATPase and β-actin in Control and Cx43-KO astrocytes. Quantification of protein levels and p-AMPK/AMPK ratio are shown in (**E**, **F**). n = 4. **G** The intracellular ATP level of Control and Cx43-KO astrocytes, n = 4. **H** Western blot showing TXNIP stability in cycloheximide (CHX)-treated Control and Cx43-KO astrocytes. **I** Quantification of TXNIP expression in (**H**) at indicated times, n = 3. **J** Schematic diagram depicting the regulation of glucose uptake and glycolysis through the AMPK/TXNIP/GLUT1 pathway. **K** Schematic representation of the anti-Cx43 IP-MS experiment. **L** KEGG enrichment analysis of differential interacting proteins. **M** Dose–response curve of IDH3α and Cx43 interaction determined by MST. **N** The representative image of immunofluorescence staining for Cx43 (green), IDH3α (red) and DAPI (blue) in the PrL region. Scale bar, 10 μm. **O** Quantitative analysis of overlap coefficient of Cx43 and IDH3α. n = 20 cells. **P** The binding mode based on Cx43–IDH3α interaction dynamics simulation, overall view (left) and local view (right). The blue solid line represents hydrogen bonding, and the gray dotted line represents hydrophobicity. **Q**, **R** Western blot analysis of membrane GLUT1 and Na-K ATPase in Cx43-KO and IDH3α^Y126F^ mutant astrocytes. n = 4. **S** Glucose uptake ability assessed by 2-NBDG uptake. n = 3. **T** The intracellular ATP level of Cx43-KO and IDH3α^Y126F^ mutant astrocytes. n = 3. **U** The real-time changes of ECAR trace in astrocytes, and the quantification (**V**) of basal glycolysis and glycolytic capacity. n = 4. Two-tailed Student’s *t* test was performed between two groups; two-way ANOVA followed by the Geisser–Greenhouse correction in (**I**); one-way ANOVA followed by Dunnett’s multiple comparisons test in multiple groups. All data represent the mean ± SEM. ^*^P < 0.05, ^**^P < 0.01, ^***^P < 0.001, ^****^P < 0^.^0001.

Aerobic glycolysis is the primary metabolic pathway of astrocytes [43]. However, deletion of Cx43 resulted in a marked reduction in glucose oxidative dependency (Fig. 3D). Tracing with ^13^C-labeled glucose revealed that Cx43-KO significantly reduced glucose utilization (Fig. 2J). To further evaluate the impact of Cx43 deletion on glucose uptake, we used the fluorescent glucose analog 2-NBDG [44], which confirmed that Cx43-KO substantially diminished glucose uptake (Fig. 5A). Moreover. immunofluorescence analysis showed a pronounced reduction in membrane-associated GLUT1 in Cx43-KO astrocytes compared to control astrocytes (Fig. 5B and C), providing a mechanistic explanation for the decrease in glucose uptake.

Membrane-associated GLUT1 is negatively regulated by thioredoxin-interacting protein (TXNIP), which facilitates the endocytosis of membrane-bound GLUT1 [45]. Our investigations revealed that Cx43 knockout led to increased ATP content (Fig. 5G), thereby suppressing AMPK phosphorylation (Fig. 5D and F). This cascade of events resulted in reduced TXNIP degradation (Fig. 5H and I). The subsequent accumulation of TXNIP promoted the endocytosis of GLUT1, resulting in a reduction in membrane-associated GLUT1 levels (Fig. 5E).

The AMPK activator metformin was employed to corroborate the role of AMPK signaling in regulating membrane-associated GLUT1 and glucose uptake following Cx43 deletion. Treatment with metformin (2.5 mM) markedly enhanced GLUT1 localization at the plasma membrane (Fig. S6A and B) and increased glucose uptake (Fig. S6C), thereby alleviating the glycolytic impairment induced by Cx43 knockout (Fig. S6D–F). The AMPK/TXNIP/GLUT1 axis is critical for astrocyte-mediated metabolic support of neurons [12], and TXNIP has been implicated as a central regulator of astrocytic glucose hypometabolism in depressive state [46]. To further define the contribution of TXNIP to Cx43-dependent regulation of glucose metabolism, we knocked down TXNIP in Cx43-KO astrocytes (Fig. S6G). TXNIP depletion significantly restored plasma membrane distribution of GLUT1, glucose uptake, and glycolytic capacity (Fig. S6H–L), indicating that intracellular accumulation of TXNIP resulting from reduced AMPK phosphorylation is a key driver of metabolic dysregulation. AMPK activation promotes TXNIP degradation [45], which predominantly occurs via the ubiquitin–proteasome pathway and requires prior phosphorylation of TXNIP [45, 47, 48]. Consistent with inhibited degradation, Cx43 deletion reduced both TXNIP phosphorylation and ubiquitination (Fig. S6M–P). Collectively, these results support a mechanism whereby Cx43 ablation suppresses AMPK phosphorylation, impairs TXNIP turnover, and thereby precipitates astrocyte glycolytic dysfunction.

AMPK inactivation is contingent upon an elevated ATP/AMP ratio [49]. Loss of Cx43 markedly increased ATP production, disrupting glucose uptake mediated by the AMPK/TXNIP/GLUT1 signaling pathway (Fig. 5J). However, the direct mechanisms by which Cx43 regulated metabolism to promote excessive ATP production, subsequently affecting glucose uptake and glycolysis, warranted further investigation.

To further elucidate the mechanisms by which Cx43 regulated astrocytic metabolism, we conducted LC-MS/MS analysis of the anti-Cx43 co-immunoprecipitation complex and identified 67 potential interacting proteins (Fig. 5K). Subsequent KEGG and GO enrichment analyses suggested that these interacting proteins were associated with carbon metabolism and the TCA cycle (Fig. 5L and Fig. S7A). Notably, IDH3α was identified among the proteins involved in carbon metabolism. IDH3 catalyzes the irreversible conversion of isocitrate to α-KG, serving as a rate-limiting enzyme in the TCA cycle, with the α subunit playing a critical role in its catalytic activity [50]. IDH3α is located within the mitochondria, while in astrocytes, Cx43 is also found within the mitochondria [29]. Hence, their interactions may be a pivotal factor in metabolic regulation.

Microscale thermophoresis (MST) was employed to investigate their binding affinity, revealing a dissociation constant (KD) of 9.73 μM for the Cx43–IDH3α interaction (Fig. 5M). We found that Cx43 partially co-localized with IDH3α in the PrL. Moreover, in the CSDS model, the expression of Cx43 and its co-localization with IDH3α in the PrL were significantly diminished (Fig. 5N and O), suggesting that the reduced interaction between Cx43 and IDH3α might contribute to the pathogenesis of depression. Subsequently, molecular docking revealed stable hydrogen bond interactions between Cx43 and IDH3α (Fig. 5P). Dynamic simulations indicated a highly stable Cx43–IDH3α complex with low flexibility and a binding free energy of −103.06 ± 3.44 kcal/mol (Fig. S7B–D). These analyses also identified Tyr126 of IDH3α as a crucial residue for interacting with Cx43 (Fig. 5P; Fig. S7E). The tyrosine residue at position 126 of IDH3α is indispensable for its catalytic function [51]. Therefore, we hypothesized that Cx43 deletion alleviated the suppression of IDH activity, thereby promoting the TCA cycle.

To recapitulate Cx43-mediated inhibition of IDH3α in a wild-type context and to functionally counteract the increased IDH3α activity caused by Cx43 deficiency, we generated Cx43-KO astrocytes expressing an IDH3α^Y126F^ mutant in which tyrosine 126 was substituted with phenylalanine (Fig. S7I). Previous work has shown that the Y126F mutation substantially abrogates IDH3α enzymatic activity [52]. Tyrosine, owing to its polar hydroxyl-containing side chain, can participate in hydrogen bonding with Cx43 (Fig. 5P) and contribute to the enzyme’s structural integrity and catalytic function [51], whereas phenylalanine is nonpolar and lacks these capabilities. To test whether Tyr126 involves the interaction between Cx43 and IDH3α, HEK-293T cells were co-transfected with Flag–Cx43 and either Myc-IDH3α (wild-type) or Myc-IDH3α^Y126F^ (Fig. S7F). Co-immunoprecipitation followed by immunoblotting revealed that the Y126F mutation markedly weakened the Cx43–IDH3α interaction (Fig. S7G and H), indicating that Tyr126 is a critical residue for their binding. Moreover, transfection of IDH3α^Y126F^ in Cx43-KO astrocytes led to increased membrane-associated GLUT1 (Fig. 5Q and R; Fig. S7J) and enhanced glucose uptake (Fig. 5S). These alterations also rescued the elevated ATP production (Fig. 5T) and the impaired glycolytic capacity (Fig. 5U and V) observed after Cx43 deletion, indicating that restoration of IDH3α activity suppression can ameliorate the abnormal increase in OXPHOS and the glycolytic dysfunction caused by Cx43 loss. Together, these results indicate that mitochondrial Cx43 maintains the low-oxidative-phosphorylation, high-glycolysis metabolic phenotype of astrocytes by inhibiting IDH3α activity.

### Lactate reduction underlies impaired neuronal OXPHOS and depressive-like behaviors following Cx43 knockout

**Fig. 6:**
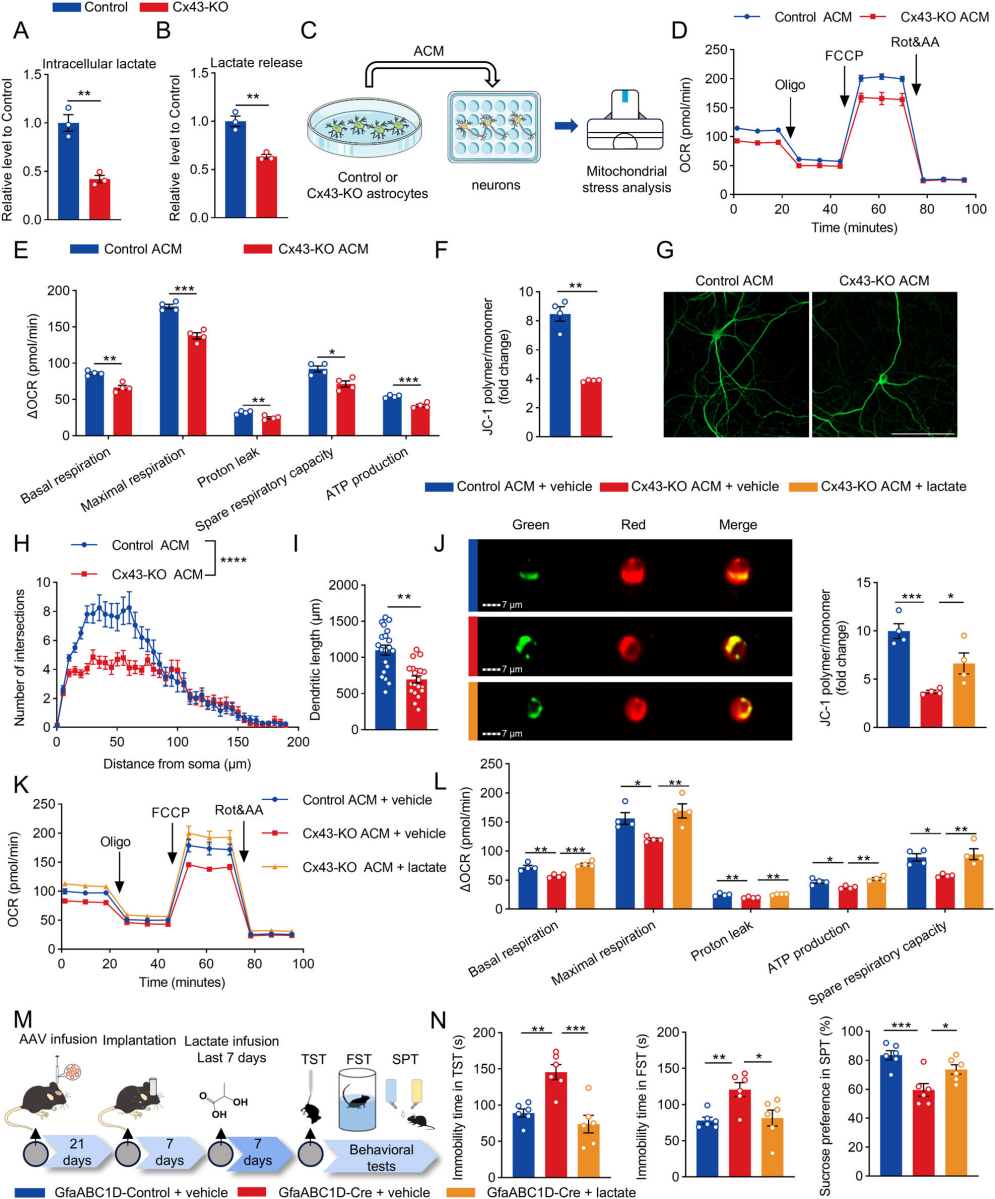
Lactate supplementation rescues neuronal OXPHOS and depressive-like behaviors induced by astrocytic Cx43 deletion. **A** The intracellular lactate level in the astrocytes. n = 4. **B** The lactate level in the astrocytic medium supernatant. n = 4. **C** The experimental diagram of neuronal OXPHOS treated with astrocyte-conditioned medium (ACM). **D** The real-time changes of neuronal OCR trace induced by ACM. **E** Quantification of basal respiration, maximal respiration, proton leak, spare respiratory capacity and ATP production in ACM-treated neurons. n = 4. **F** The mitochondrial membrane potential detected by JC-1 staining in neurons. n = 4. **G** MAP2 staining of ACM-treated neurons (scale bar, 100 μm) and their Sholl analysis (**H**) and quantification of the length of dendritic branches (**I**). n = 20 neurons. **J** The mitochondrial membrane potential of ACM-treated neurons detected by JC-1 staining. n = 4. **K** The real-time changes of OCR trace in neurons. **L** Quantification of basal respiration, maximal respiration, proton leak, ATP production and spare respiratory capacity. n = 4. **M** The schematic diagram and timeline of lactate infusion in mice. **N** Assessment of immobility time in the TST and FST and sucrose preference in the SPT. n = 6. Two-tailed Student’s *t* test was performed between two groups; two-way ANOVA followed by the Geisser–Greenhouse correction in **H**; one-way ANOVA followed by Dunnett’s multiple comparisons test was performed in multiple groups. All data represent the mean ± SEM. ^*^P < 0.05, ^**^P < 0.01, ^***^P < 0.001.

The lactate shuttle represents a critical mechanism through which astrocytes supply energy substrates to neurons [11]. Extracellular lactate enters neurons via the MCT2 and is reconverted into pyruvate to engage in the TCA cycle [6, 10, 11]. Knockout of Cx43 resulted in a reduction of both intracellular and released lactate levels in astrocytes (Fig. 6A and B). To investigate whether the reduction in astrocytic lactate affects the neuronal OXPHOS, we replaced the neuronal culture medium with astrocyte-conditioned medium (ACM) (Fig. 6C). Firstly, an MCT2 blocker (AR-C155858) was employed (Fig. S8A) to determine whether lactate in the ACM affects the neuronal OXPHOS, which revealed a significant reduction in neuronal mitochondrial respiratory function. Specifically, basal respiration, maximum respiration, and ATP production were all markedly diminished (Fig. S8B and C), underscoring the critical role of lactate in the ACM in supporting neuronal OXPHOS (Fig. S8D). Next, we observed that the neuronal OCR in the Cx43-KO ACM were significantly lower than those in the Control ACM group both before and after FCCP addition (Fig. 6D), indicating a significant decline in basal respiration, maximum respiration, spare respiratory capacity, proton leak, and ATP production (Fig. 6E). The compromised mitochondrial respiration was further evidenced by a decrease in the membrane potential, as shown by JC-1 staining (Fig. S9A and B). The ratio of JC-1 polymers to monomers was significantly decreased in neurons incubated with Cx43-KO ACM (Fig. 6F). Sholl analysis demonstrated a significant reduction in dendritic complexity (Fig. 6G and H) and total dendrite length (Fig. 6I) in neurons treated with Cx43-KO ACM compared with those treated with Control ACM. These findings indicated that the loss of Cx43 in astrocytes impaired neuronal OXPHOS and their dendritic complexity, providing direct evidence of disrupted lactate homeostasis and resultant neuronal dysfunction due to Cx43 deletion in vitro.

To investigate the biological role of reduced lactate in neuronal energy metabolism in Cx43-KO ACM, we supplemented ACM with 2 mM lactate. JC-1 staining showed that lactate supplementation significantly restored mitochondrial membrane potential (Fig. 6J; Fig. S9C and D) and improved neuronal OXPHOS (Fig. 6K and L), indicating a reversal of neuronal mitochondrial dysfunction caused by Cx43-KO ACM. Moreover, lactate also significantly ameliorated the reduction in branching complexity of primary neurons induced by Cx43-KO ACM (Fig. S10A and B).

To assess the impact of astrocyte-derived lactate on dendritic complexity within depression- associated mPFC subregions (ACC, PrL, and infralimbic cortex (IL)), we analyzed acute brain slices from Thy1-GFP transgenic mice subjected to CSDS. Dendritic complexity was quantified by measuring the area of GFP fluorescence. Cx43-KO ACM reduced terminal dendritic complexity across the PrL, IL, and ACC versus Control ACM. Supplementation with lactate markedly ameliorated the observed decrease in dendritic complexity (Fig. S10C–F). Whole-cell recordings from pyramidal neurons revealed that, following 2 h of exposure to Cx43-KO ACM, no significant alterations in resting membrane potential or input resistance were observed across the three subregions (Fig. S11A–F). In contrast, treatment with Cx43-KO ACM led to a reduction in neuronal firing frequencies in response to depolarizing current injections, whereas lactate supplementation significantly augmented firing frequencies (Fig. S11G–I). These findings underscore the pivotal role of lactate in preserving neuronal dendritic structure and excitability.

Next, we administered lactate into the PrL, where astrocytic Cx43 was specifically knocked out, and observed significant improvements in depressive-like behaviors after 7 days of lactate supplementation (Fig. 6M), as manifested by reduced immobility times in the TST and FST and increased sucrose preference in the SPT (Fig. 6N). These results further indicate that the decreased astrocyte-derived lactate is a critical factor in the neuronal mitochondrial dysfunction and depressive-like behaviors in astrocytic Cx43 knockout models.

### Cx43 knockout disrupts astrocytic glycolytic responses to heightened neuronal activity

In addition to physiological glycolysis, there exists a distinctive mechanism that promotes lactate production during periods of heightened neuronal excitability, known as glutamate-driven glycolysis [18]. Upon neuronal activation, released glutamate is transported into astrocytes through EAAT2 in a Na^+^-dependent manner. Within astrocytes, the influx of glutamate drives the occurrence of glycolysis [13]. Due to insufficient mitochondrial distribution in peripheral astrocytic processes (PAPs), the ATP required for this process is generated by membrane-associated glycolytic enzyme PGK through localized glycolysis [53–56].

To further investigate the impact of Cx43 on the metabolic activity of astrocytes during heightened neuronal excitability, we established a cell model that simulated neuronal excitation through glutamate with the concentration at 200 μM. Initially, we noted that the knockout of Cx43 did not alter the membrane distribution of the glutamate transporter EAAT2 on astrocytes (Fig. S12A and B). Consistent with previous reports [18], glutamate stimulation significantly enhanced glycolysis and lactate production in astrocytes. However, Cx43 knockout markedly diminished this glycolytic response (Fig. S12C–E), indicating that Cx43 was essential for glutamate-driven glycolysis. Through 2-NBDG uptake assays, we found that glutamate significantly augmented glucose uptake in astrocytes, whereas the loss of Cx43 conspicuously inhibited this increase (Fig. S12F). This suggested that Cx43 knockout directly inhibited the augmentation of lactate synthesis at the metabolic substrate level.

Furthermore, our results demonstrated that glutamate stimulation significantly enhanced PGK activity, while the absence of Cx43 inhibited this effect (Fig. S12G). Prior research has indicated that PGK activity is regulated by AMPK [57]. We observed that Cx43 deficiency led to reduced AMPK phosphorylation (Fig. 5D, F), which may explain the decrease in PGK activity. Notably, Cx43 knockout resulted in a substantial reduction in PGK activity, whereas treatment with the AMPK activator metformin significantly restored it (Fig. S12H). These findings support the hypothesis that reduced AMPK activation due to Cx43 deficiency impairs PGK activity.

### Mitochondria-specific overexpression of Cx43 restores astrocytic metabolism and mitigates depressive-like behaviors

**Fig. 7:**
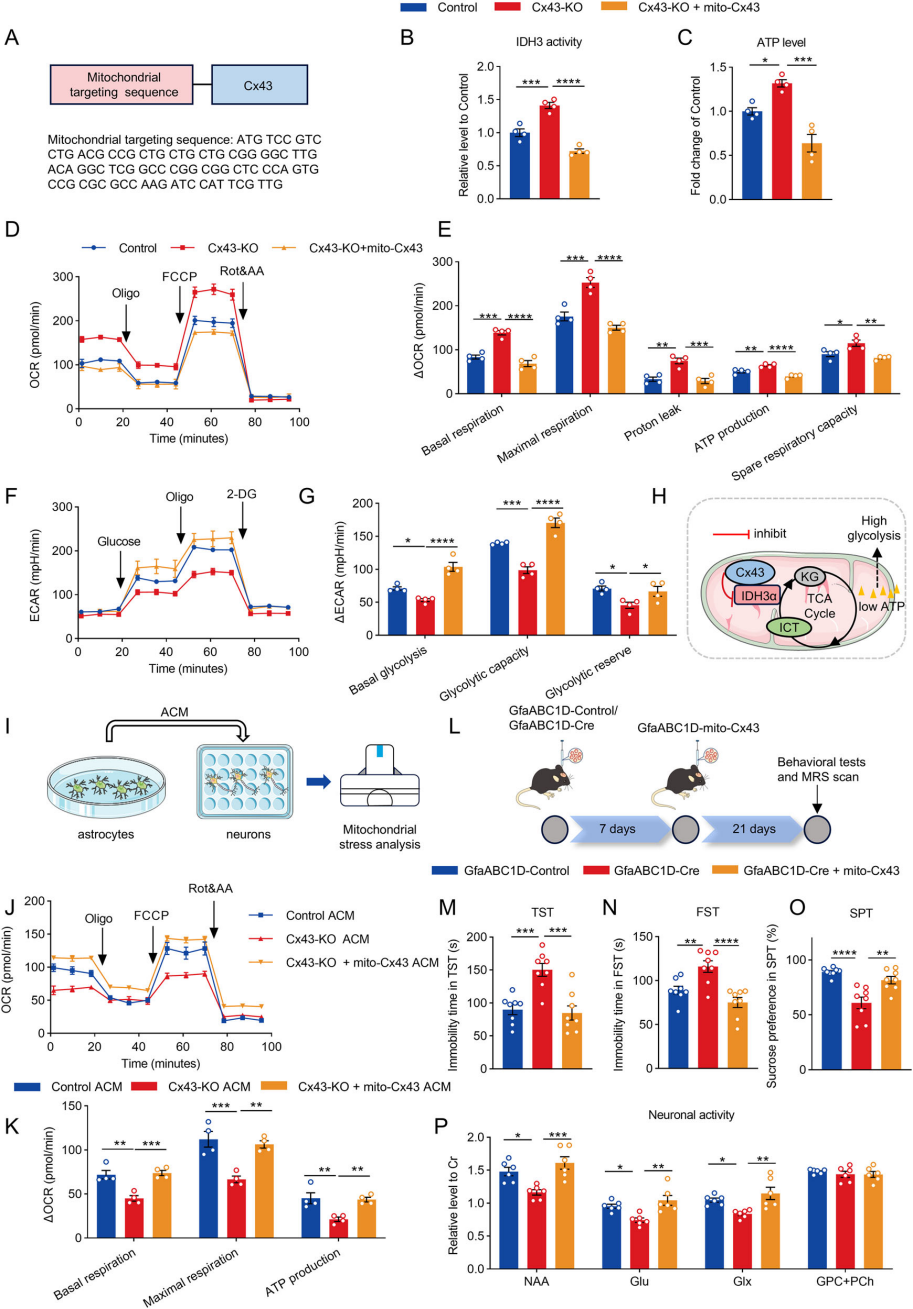
Mitochondria-specific overexpression of Cx43 ameliorates neuronal OXPHOS and depressive-like behaviors in mice induced by astrocytic Cx43 knockout. **A** Mitochondria targeting sequence. **B** The enzyme activities of mitochondrial isocitrate dehydrogenase 3 (IDH3). n = 4. **C** The intracellular ATP level of astrocytes. n = 4. **D** The real-time changes of OCR traces in astrocytes. n = 4. **E** Quantification of basal respiration, maximal respiration, proton leak, ATP production and spare respiratory capacity. n = 4. **F** The real-time changes of ECAR traces in astrocytes. **G** Quantification of basal glycolysis, glycolytic capacity and glycolytic reserve. n = 4. **H** Schematic diagram depicting the regulatory roles of Cx43 in astrocytic metabolism. **I** The experimental diagram of neurons treated with astrocyte-conditioned medium (ACM). **J** The real-time changes of OCR traces in neurons. n = 4. **K** Quantification of basal respiration, maximal respiration and ATP production. n = 4. **L** Temporal flowchart of the model construction. **M**–**O** Immobility time in the TST and FST and sucrose preference in the SPT in mice, n = 8 mice per group. **P** The relative metabolite concentrations in the PrL detected by in vivo ^1^H MRS. n = 6. One-way ANOVA followed by Dunnett’s multiple comparisons test was performed in multiple groups. All data represent the mean ± SEM. ^*^P < 0.05, ^**^P < 0.01, ^***^P < 0.001, ^****^P < 0.0001.

In the aforementioned study, we elucidated the noncanonical role of mitochondrial Cx43 in astrocyte metabolic homeostasis and its critical mechanisms in the pathogenesis of depression. To further validate the feasibility of targeting mitochondrial Cx43 in astrocytes for antidepressant treatment, we established a model with mitochondria-specific overexpression of Cx43 (Fig. 7A). This was achieved by inserting a mitochondrial targeting sequence into a vector encoding the Cx43 gene [58, 59]. Notably, astrocyte-specific mitochondrial overexpression of Cx43 significantly restored the decreased IDH3 enzymatic activity (Fig. 7B) and intracellular ATP levels (Fig. 7C) observed upon Cx43 knockout. Moreover, mitochondrial Cx43 overexpression inhibited the aberrant increase in OXPHOS and the concomitant reduction in glycolytic activity induced by Cx43 deletion (Fig. 7D–G), demonstrating that physiological levels of astrocytic mitochondrial Cx43 were essential for maintaining a low oxidative phosphorylation and high glycolysis metabolic phenotype (Fig. 7H).

Furthermore, using a conditioned medium neuronal culture model (Fig. 7I), we found that overexpression of mitochondrial Cx43 in astrocytes ameliorated the impaired neuronal OXPHOS caused by Cx43-KO ACM (Fig. 7J and K). At the animal level, targeted astrocytic mitochondrial Cx43 overexpression in the PrL of astrocyte-specific Cx43 knockout mice significantly ameliorated depressive-like behaviors (Fig. 7L–O). ^1^H MRS revealed that astrocytic mitochondrial Cx43 overexpression in the PrL restored neuronal activity markers, including NAA and Glu/Glx levels, which were reduced upon Cx43 deletion, indicating recovery of neuronal function (Fig. 7P). Collectively, these findings establish mitochondrial Cx43 in astrocytes as a pivotal regulator of normal neuronal activity and a promising target for antidepressant treatment.

## Discussion

**Fig. 8:**
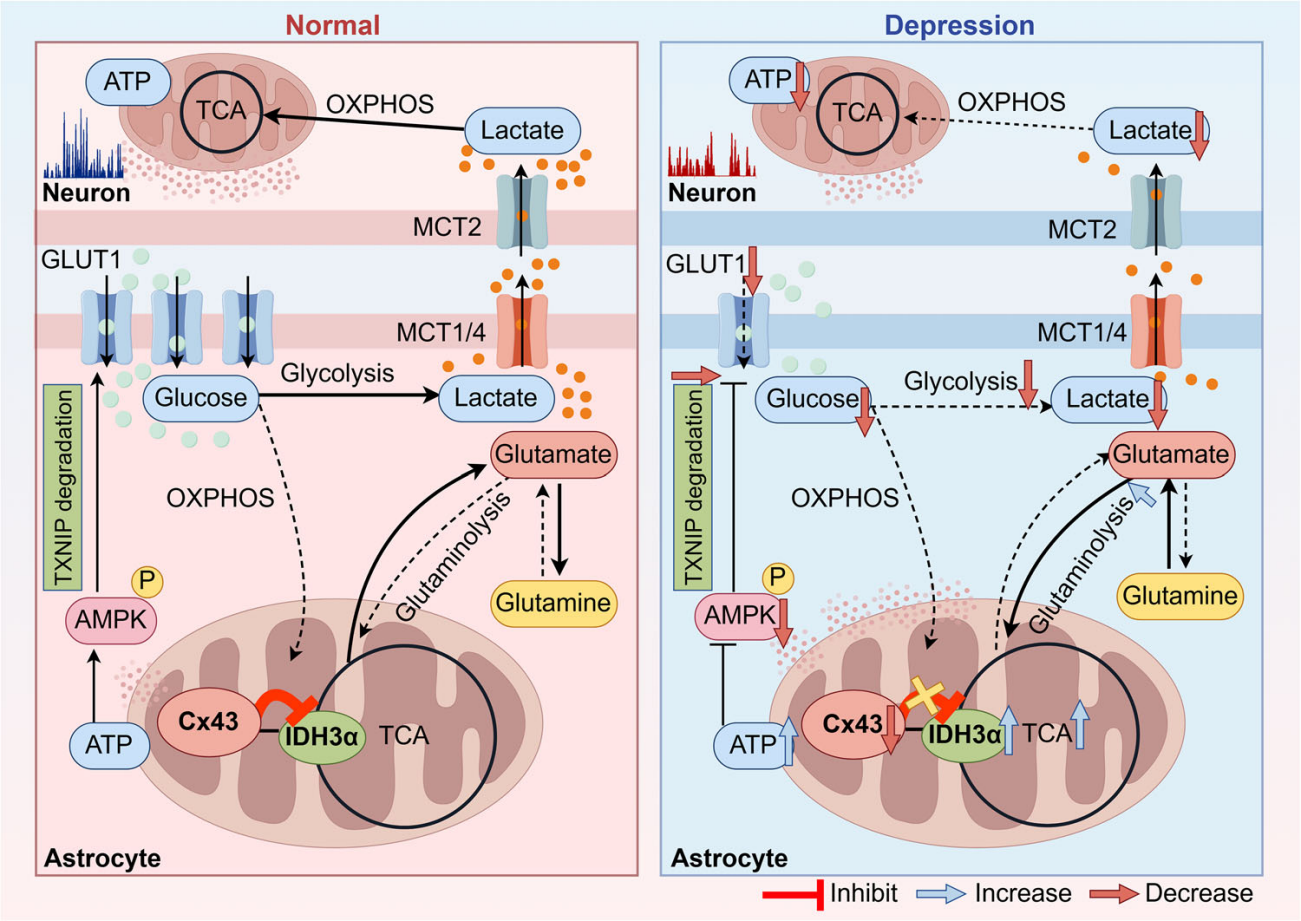
Schematic illustrating the noncanonical role of mitochondrial Cx43 in the regulation of astrocyte glucose metabolism. Under physiological conditions, mitochondrial Cx43 interacts with IDH3α, restraining tricarboxylic acid (TCA) cycle activity and thus maintaining relatively low intracellular ATP levels in astrocytes. The consequent low ATP/AMP ratio activates AMPK, which promotes TXNIP degradation and thereby stabilizes membrane-localized GLUT1, enhancing glucose uptake and glycolytic flux and increasing lactate production for neuronal use. By contrast, in the pathological context of depression, loss of mitochondrial Cx43 relieves inhibition of IDH3α, driving glutamine-fueled oxidative phosphorylation and excessive ATP generation. Elevated ATP suppresses AMPK activation, resulting in intracellular accumulation of TXNIP, impaired plasma membrane localization of GLUT1, reduced glucose uptake and glycolysis, and diminished lactate release from astrocytes, thereby limiting lactate availability to neurons and contributing to neurological dysfunction. (Fig. 8 was generated by Figdraw).

In this study, we demonstrate that the knockout of astrocytic Cx43 in the PrL leads to reduced lactate levels, decreased neuronal excitability, and impaired brain functional connectivity, all indicative of clinical depressive states. Interestingly, the cellular metabolic phenotype changes induced by Cx43 loss are independently of its gap junction function. Mechanistically, we identified a previously unrecognized interaction between Cx43 and IDH3α within mitochondria, which negatively regulates IDH3 activity and plays a critical role in maintaining astrocytic metabolic homeostasis. The attenuation of Cx43 results in a diminished inhibitory influence on mitochondrial IDH3α, leading to a substantial enhancement of OXPHOS and an increase in intracellular ATP production. This alteration is accompanied by a reduction in glucose uptake and glycolytic flux, a decline in lactate levels, and an inadequate neuronal energy supply, ultimately facilitating the pathogenesis of depression (Fig. 8). Our findings underscore the noncanonical role of astrocytic Cx43 in regulating glucose uptake and metabolic homeostasis, offering a novel therapeutic strategy for depression by targeting astrocytic metabolic restoration.

The reduced expression of Cx43 in the PrL region is a significant pathological feature of depression [19, 23]. However, the mechanisms underlying the diminished expression of Cx43 remain incompletely understood. Our preliminary studies have indicated that corticosterone, a key glucocorticoid elevated during stress, significantly reduces the expression of Cx43 in astrocytes within the PFC while simultaneously promoting the phosphorylation of Cx43 at serine 368 and tyrosine 265 [60, 61]. Furthermore, corticosterone facilitates the formation of annular gap junctions by Cx43 in astrocytes, which subsequently activates the autophagy-lysosomal pathway and enhancing Cx43 degradation [60, 62]. These findings suggest a close association between elevated corticosterone levels induced by stress and the reduced level of Cx43 in astrocytes, providing a potential explanation for the observed pathological changes in the CSDS mouse model.

Distinct brain regions exhibit variable glycolytic capabilities, with the PFC demonstrating notably high glycolytic activity [3]. Lactate, a key by-product of glycolytic metabolism, is significantly reduced in the PFC of individuals with depression [6, 63]. Notably, antidepressant treatment mitigates this reduction, highlighting a strong association between lactate levels and depressive states [16, 17]. In our investigation, astrocyte-specific knockout of Cx43 in the PrL resulted in a pronounced reduction of lactate, concomitant with decreased neuronal excitability and reduced levels of the excitatory neurotransmitter glutamate. These findings suggest that astrocytic Cx43 is crucial for maintaining lactate homeostasis in depressive disorders. Unlike neurons, astrocytes rely predominantly on aerobic glycolysis as their principal metabolic pathway under physiological conditions, releasing lactate mainly through the monocarboxylate transporters MCT1 and MCT4 to support neurons with energy substrates, whereas mitochondrial OXPHOS remains comparatively minimal [10–12, 43]. Our research uncovered that astrocytic deletion of Cx43 resulted in a comprehensive repression of glucose metabolism and, ultimately, a notable reduction in lactate production. These metabolic changes were consistent with the metabolic hallmarks found in depression [46].

Cx43, the most abundant member of the connexin family, predominantly forms gap junctions to facilitate intercellular metabolite exchange, a process essential for supporting neuronal activity [19, 24, 25, 64]. This raises the question of whether the metabolic alterations observed in Cx43-KO astrocytes are dependent on its gap junction function. Our study revealed that the regulation of metabolism by Cx43 occurred independently of its canonical gap junction function (Fig. 4). The noncanonical roles of Cx43 have garnered considerable attention. Nevertheless, its specific regulatory functions remain unclear. In addition to its localization at the plasma membrane, where it forms gap junction channels and hemi-channels, Cx43 is also situated within the cytoplasm and organelles such as mitochondria, where it engages in protein-protein interactions that facilitate the regulation of diverse physiological processes within the cell [27, 65]. Given that mitochondria are the most critical organelles for cellular energy metabolism [34], mitochondrial Cx43 may play a pivotal role in regulating cellular respiration and metabolism [26]. In cardiomyocytes, mitochondrial Cx43 interacts with proteins associated with cellular respiration, modulating the production of reactive oxygen species (ROS) and ATP [28, 66], while also contributing to the maintenance of ionic homeostasis within the mitochondria [29, 67]. However, despite the presence of Cx43 in the mitochondria of astrocytes [29], its specific functions remain inadequately defined. Our analysis elucidated that IDH3α, the catalytic subunit of IDH3, serves as a crucial interacting partner of Cx43. The interaction between mitochondrial Cx43 and IDH3α sustains a metabolic profile characterized by low OXPHOS and high glycolysis in astrocytes. Thus, in depressive states, the downregulation of Cx43 compromises gap junction-mediated intercellular communication, thereby promoting neuroinflammatory responses and other core pathological features of depression [23, 68, 69]. Meanwhile, through a noncanonical mechanism involving intracellular protein-protein interactions, this reduction in Cx43 expression drives astrocytic metabolic reprogramming and impairs lactate support for neurons. Collectively, these disturbances disrupt cerebral homeostasis, which is mechanistically linked to the emergence of depressive-like behaviors.

The generation of ATP is intricately linked to astrocytic responses to glutamate [70], with the influx of glutamate further promoting glycolysis [13, 18]. In this study, we observed an interesting phenomenon in which the loss of Cx43 induces metabolic reprogramming in astrocytes, characterized by an increase in ATP production concomitant with a reduction in lactate generation. Several factors may contribute to this metabolic alteration. Firstly, the absence of Cx43 directly reduced the distribution of GLUT1 on the cell membrane (Fig. 5), thereby inhibiting glycolysis at the source of metabolic substrate. Secondly, while Cx43 deficiency promoted mitochondrial ATP production, it concurrently reduced the generation of glycolytic ATP. Although the activity of the key glycolytic enzyme Na-K ATPase is fueled by both glycolytic and mitochondrial-derived ATP [18, 53], astrocytic processes located adjacent to neuronal synapses, known as PAPs, lack mitochondria and predominantly rely on glycolytic ATP to respond to rapid excitatory synaptic activity [54, 55]. PGK is a membrane-bound glycolytic enzyme that catalyzes the production of ATP during glycolysis and plays a crucial role in driving Na-K ATPase activity and regulating the influx of glutamate [56, 71]. In the context of Cx43 deficiency, the inhibition of AMPK activation (Fig. 5) led to a reduction in the activity of PGK (Fig. S12). This resulted in diminished ATP generated from glycolysis and impaired the mechanism by which glutamate influx stimulates glycolysis. Thirdly, Cx43 deficiency was associated with increased glutamine-fueled anaplerosis and a concomitant reduction in glutamate synthesis (Figs. 2 and 3), which lowered the levels of glutamate and glutamine within the brain (Fig. 1C and D), ultimately leading to reduced neuronal excitability (Fig. 1 and Fig. S11) and further compromising glycolytic activity driven by glutamate. Thus, the loss of Cx43 not only obstructs glycolysis in astrocytes under resting conditions but also impairs their ability to adequately support neuronal metabolism, contributing to the dysregulation of neural function and the emergence of depressive-like behaviors.

In conclusion, this study elucidates a noncanonical role of astrocytic Cx43 within mitochondria, demonstrating its critical involvement in maintaining the unique glucose metabolic profile of astrocytes through interaction with IDH3α. Inhibition of IDH3 activity by Cx43 leads to reduced ATP production while promoting a high rate of glucose uptake, thereby providing sufficient substrate for lactate production and sustaining neuronal excitability. A decrease in Cx43 levels in the PrL triggers metabolic reprogramming in astrocytes, characterized by reduced lactate production and increased glutamine consumption. This metabolic reprogramming results in a significant deficiency of energy substrates and neurotransmitter precursors essential for neuronal excitability, ultimately contributing to the manifestation of depressive-like symptoms. These findings position mitochondrial Cx43 in astrocytes as a promising therapeutic target for depression, suggesting a potential strategy for restoring astrocytic metabolic homeostasis.

## Methods and materials

### Animals

All animal care and experimental procedures adhered to the guidelines outlined in the National Institutes of Health (NIH) Guide for the Care and Use of Laboratory Animals and were approved by the Institutional Animal Care and Use Committee of Chinese Academy of Medical Sciences and Peking Union Medical College (Ethics number: 00005246). The animal study complied with the ARRIVE guidelines.

Male C57BL/6 (6–8 weeks old) mice and male CD-1 mice (4–6 months old) were obtained from Charles River Laboratories (Beijing, China). Male Thy1-GFP transgenic mice (6–8 weeks old) were provided by the Institute of Genetics and Developmental Biology, Chinese Academy of Sciences. All animals were housed under standard housing conditions with unrestricted access to food and water. Animals were allocated to experimental groups using a randomization method.

### Stereotaxic injection

Mice were anesthetized with isoflurane (1.5–2%) and secured in a stereotaxic apparatus (RWD Life Science, China). A scalp incision was made, and two small holes were drilled into the skull, one on each side. The cannula was implanted unilaterally in the right PrL, coordinates from bregma: AP, +2.34 mm; DV, −2 mm; ML, −0.25 mm) and affixed to the skull using dental cement. Following surgery, the mice recover for at least 1 week. On the injection days, 1 μL of 10 mM lactate (aladdin, China) or saline solution was administered through an internal cannula at a speed of 0.1 μL/min, controlled by a microinjector (Lead Fluid, China). For the vector injection, 500 nL of the viral vector was bilaterally injected into the PrL (coordinates from bregma: AP, +2.34 mm; DV, −2 mm; ML, ±0.25 mm) at a speed of 50 nL/min. 4 weeks after recovery from anesthesia on a heating pad, the mice were subjected to further experiments. Recombinant AAV vectors, including AAV-MG1.2-Cx3cr1-3xFLAG-WPRE, AAV2/9-GfaABC1D-3xFLAG-WPRE, AAV-MG1.2-Cx3cr1-Cre-WPRE, AAV2/9-GfaABC1D-Cre-WPRE, AAV2/9-GfaABC1D-mito-Cx43-WPRE and AAV2/9-GfaABC1D-Cx43(T154A)-WPRE were constructed and packaged by OBiO Technology (Shanghai, China).

### Cell culture

The immortalized mouse astrocytes (JN0661-B) were obtained from GuangZhou Jennio Biotech Co., Ltd, and were derived from primary mouse astrocytes (M1800-57, ScienCell, USA). The Cx43-KO astrocytes were generated from the immortalized mouse astrocytes using the CRISPR/Cas9 system by OBiO Technology (Shanghai, China). HEK-293T cells (CL-0005) were purchased from Procell (Wuhan, China). Astrocytes and HEK-293T cells were cultured in a high-glucose DMEM medium (Thermo Fisher Scientific, USA) consisting of 10% fetal bovine serum (Thermo Fisher Scientific, USA) and 1% penicillin/streptomycin at 37 °C in a 5% CO_2_ incubator. The cells used in all experiments were within 10 passages, and astrocytes exceeded 90% confluence at the time of metabolic assays. All cell lines were recently authenticated by STR profiling and tested negative for mycoplasma contamination.

Primary neuronal cultures were established by dissociating the cortices from embryonic day 18 C57BL/6 mouse embryos. The neurons were maintained in neurobasal medium (Thermo Fisher Scientific, USA) supplemented with 2% B27 supplement (Thermo Fisher Scientific, USA), 1% GluMAX (Thermo Fisher Scientific, USA), and 1% penicillin/streptomycin at 37 °C in a 5% CO_2_ incubator. The medium was refreshed every 3 days by replacing half the medium with fresh medium.

### Statistical analysis

Statistical analyses were performed using MATLAB R2013b (The MathWorks, USA) and GraphPad Prism 9.5.0 software (GraphPad Software, USA). All data are presented as mean ± standard error of the mean (SEM). Data distribution was assessed using the Shapiro–Wilk test. Statistical significance between the two groups was determined using two-tailed Student’s *t* test, Welch’s *t*-test, or the Mann–Whitney test. Multiple group comparisons were conducted using one-way analysis of variance (ANOVA) followed by Dunnett’s multiple comparisons test. Comparisons between two independent variables were assessed using two-way ANOVA followed by Geisser–Greenhouse correction. Statistical significance was set at *P* < 0.05.

## Supplementary information

Supplementary Information

Original blots

Reproducibility checklist

## Data Availability

The datasets used and/or analyzed during the current study are available from the corresponding authors upon reasonable request. Original blots are provided in the Supplemental Material. More detailed methods can be found in Supplementary Information.
