# Supplementary material for: Noncanonical role of astrocytic mitochondrial Cx43: suppressing IDH3α to sustain glycolytic homeostasis against depression
Source: Cell Death Dis. 2025 Dec 8;17(1):94. doi: 10.1038/s41419-025-08309-1 (PMC12830930; doi:10.1038/s41419-025-08309-1)
Supplement: Supplementary file 1 — Supplementary Information [file 41419_2025_8309_MOESM1_ESM.docx]

**Supplementary Information**

**Noncanonical role of astrocytic mitochondrial Cx43: suppressing IDH3α to sustain glycolytic homeostasis against depression​**

**Authors**

Junrui Ye†, Hongyun Wang†, Ye Peng, Shasha Wang, Ruifang Zheng, Yuqi Chen, Ruolan Yuan, Zhenzhen Wang, Xu Yan, Wenbin He, Gang Li, Hongshuo Sun, Zhongping Feng, Shifeng Chu^*^, Zhao Zhang^*^, Naihong Chen^*^

†, These authors contributed equally to this work: Junrui Ye, Hongyun Wang

*Corresponding authors. State Key Laboratory of Bioactive Substances and Functions of Natural Medicines, Institute of Materia Medica & Neuroscience Center, Chinese Academy of Medical Sciences and Peking Union Medical College, 2 Nanwei Road, Xicheng district, Beijing, 100050, China.

E-mail addresses: chushifeng@imm.ac.cn; zhangzhao@imm.ac.cn; chennh@imm.ac.cn

**The file includes:**

1. Supplementary figures (Figures S1–S12)

2. Supplementary table (Table S1)

3. Supplementary methods

4. References

**1. Supplementary figures**


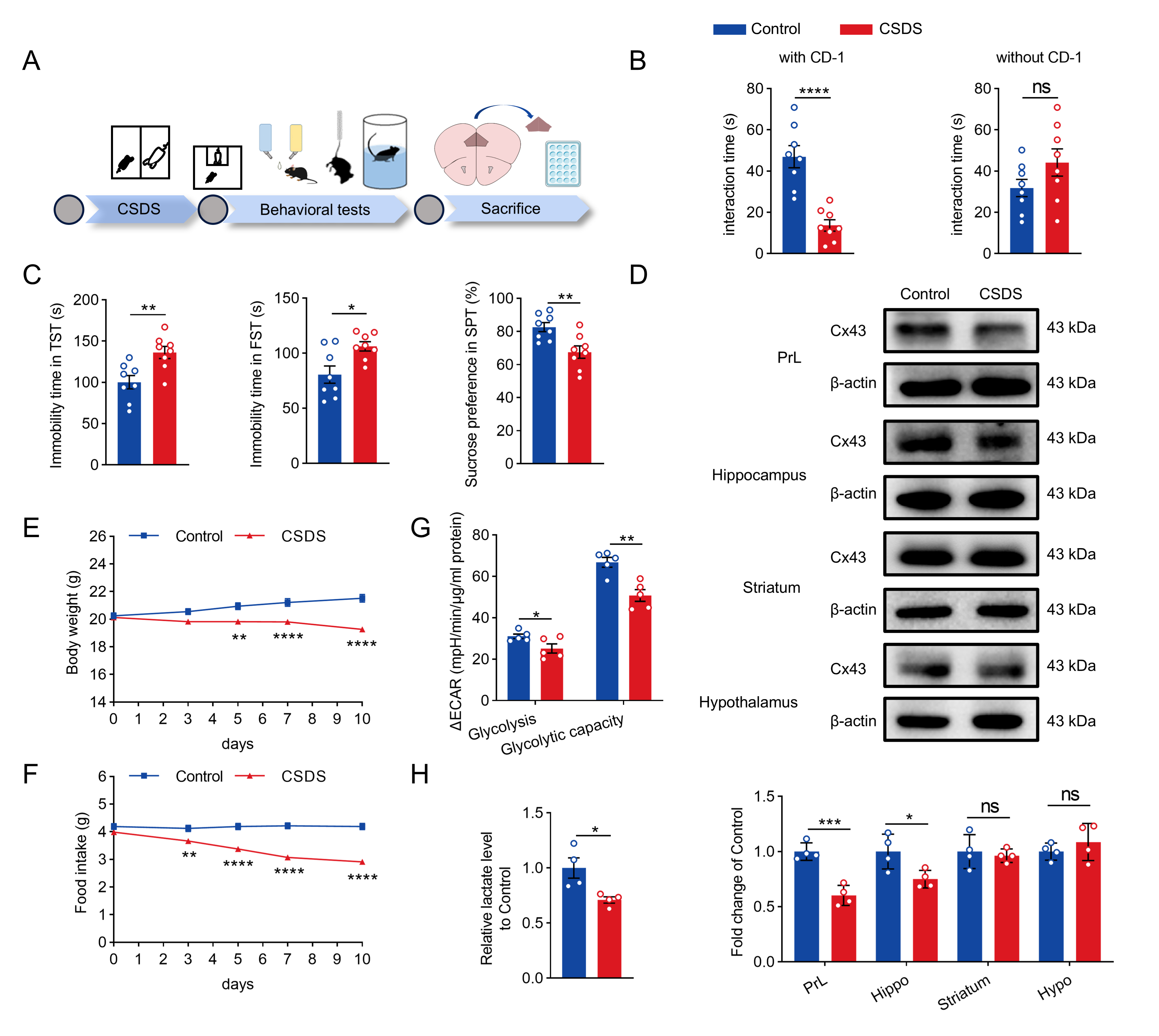


**Fig. S1.** Reduced glycolytic capacity and lactate levels in the PrL region of the chronic social defeat stress (CSDS) model. (A) The experimental diagram of the generation of the CSDS model. (B) The interaction time in the social interaction test. n = 8. (C) Immobility time in the TST and FST, and sucrose preference in the SPT. n = 8. (D) The expression of Cx43 in the PrL, hippocampus (Hippo), striatum, and hypothalamus (Hypo) assessed by Western blot. n = 4. (E) Changes in body weight of mice over the 10-day period. n = 8. (F) Daily food intake variations in mice during the 10-day period. n = 8. (G) Quantitative glycolytic capacity of PrL by Seahorse XF analysis. n = 5. (H) The level of lactate in the PrL detected by the lactate assay kit. n = 4. Two-tailed Student’s *t*-test was performed between two groups. Two-way ANOVA followed by Geisser–Greenhouse correction in E and F. All data represent the mean ± SEM. ns, no significant difference. ^*^*P* < 0.05, ^**^*P* < 0.01, ^***^*P* < 0.001 ^****^*P* < 0.0001.


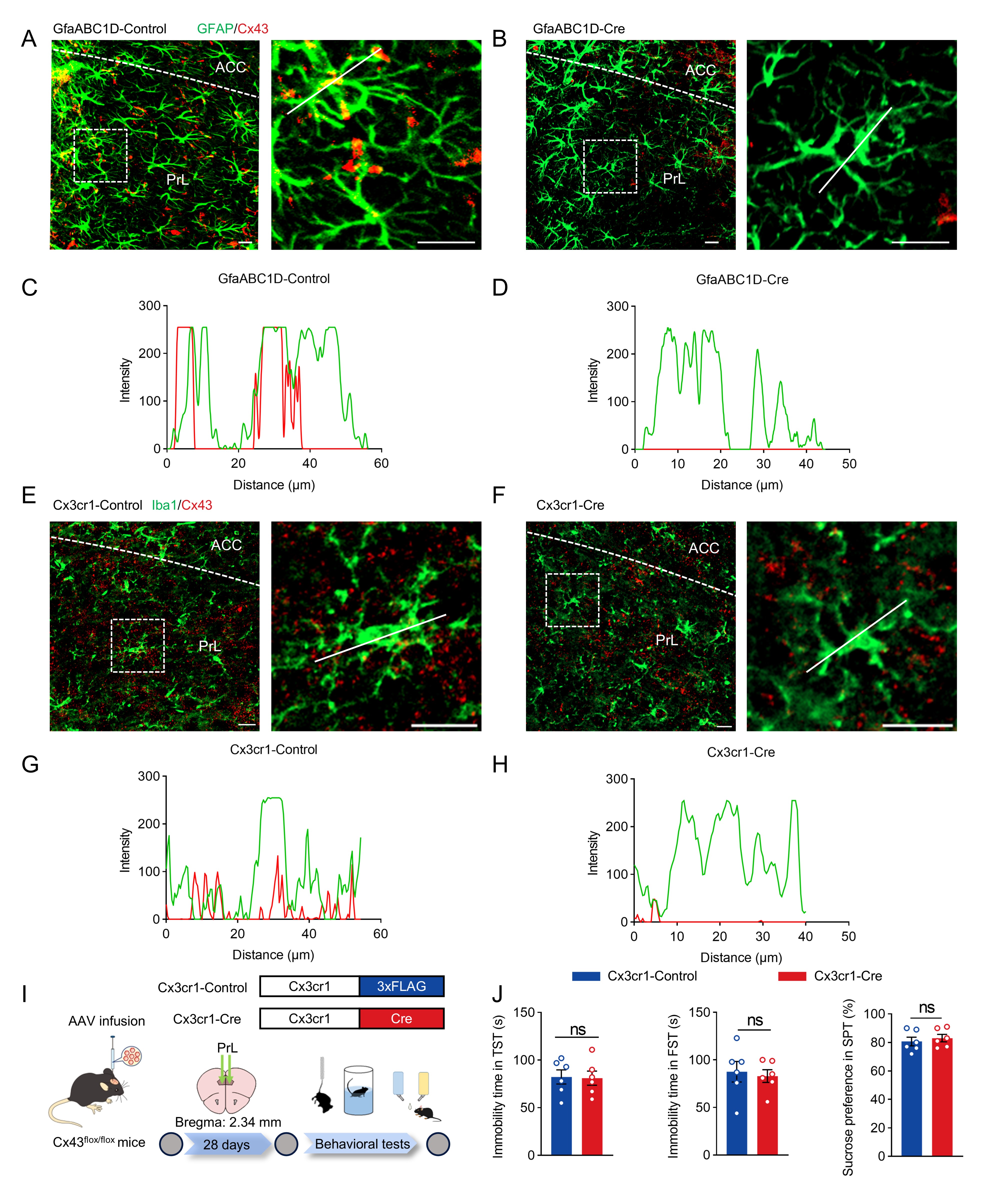


**Fig. S2.** Cellular-specific Cx43 deletion validation and no depressive-like phenotypes induced by microglia-specific Cx43 knockout. (A–B) Validation of astrocyte-specific Cx43 knockout with representative immunofluorescence images demonstrating colocalization of GFAP^+^ astrocytes (green) and Cx43 (red). Scale bar, 20 μm. (C–D) Fluorescence intensity profiles along a designated white line across the cell body indicate the colocalization of GFAP and Cx43. (E–F) Validation of microglia-specific Cx43 knockout with representative immunofluorescence images demonstrating colocalization of Iba1^+^ microglia (green) and Cx43 (red). Scale bar, 20 μm. (G–H) Fluorescence intensity profiles along a designated white line across the cell body indicate the colocalization of Iba1 and Cx43. (I) The experimental flowchart of the generation of the microglia-specific Cx43 knockout mice. (J) Immobility time in the TST and FST, and sucrose preference in the SPT. n = 6. Two-tailed Student’s *t*-test was performed between two groups. All data represent the mean ± SEM. ns, no significant difference.


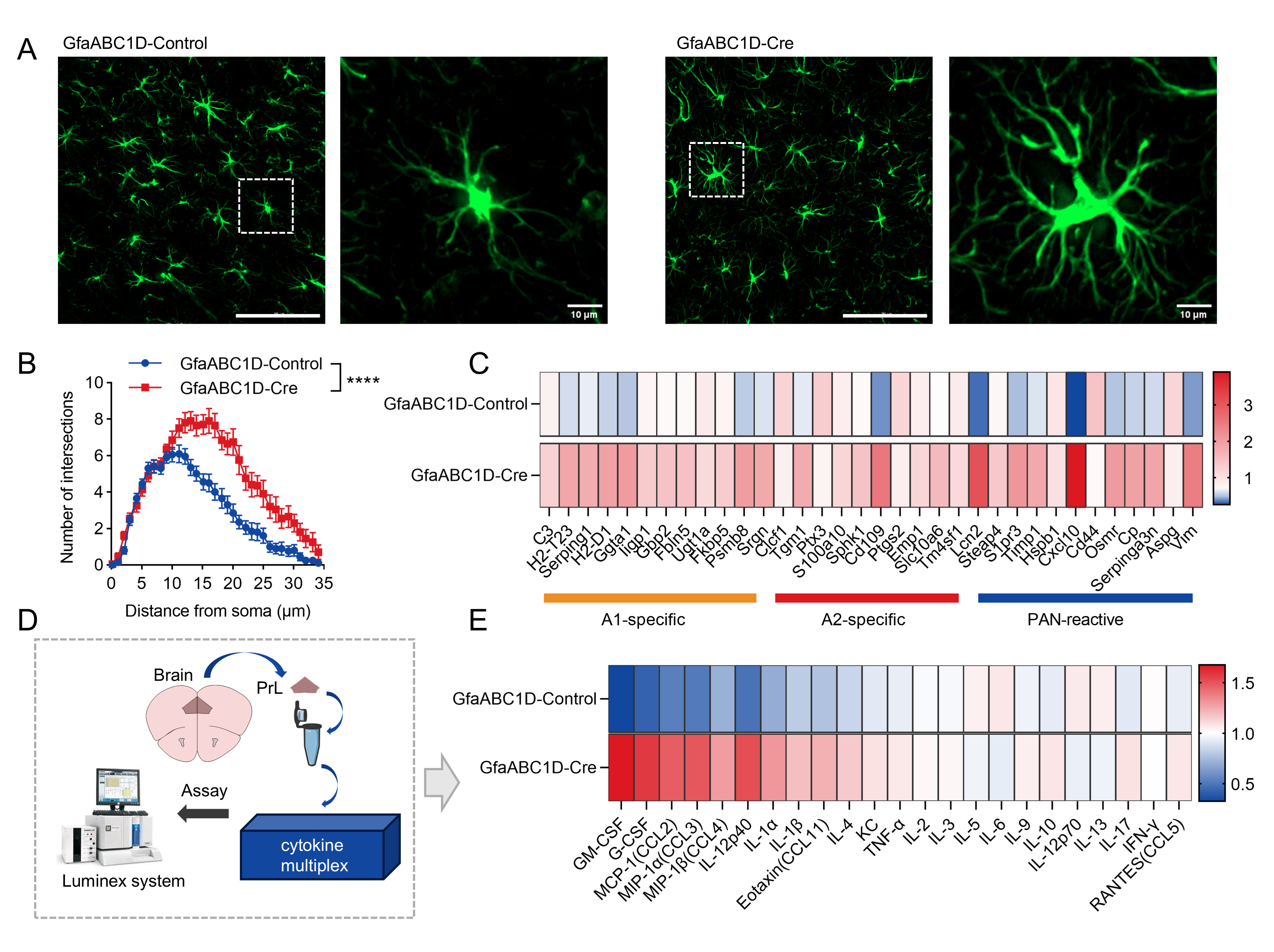
 **Fig. S3.** Astrocytic-specific knockout of Cx43 causes the reactive astrocytic phenotype. (A) Representative immunofluorescence images of GFAP. Scale bar, 100 μm (left), 10 μm (right). (B) Sholl analysis of the branch complexity of astrocytes. n = 20 astrocytes. (C) Heatmap of astrocytic activation-related gene expression in the isolated PrL tissue quantified by qPCR. (D) The experimental diagram of cytokine multiplex assay. (E) Cytokine and chemokine level in the PrL measured by multiplex Luminex assay. n = 3. Two-way ANOVA followed by the Geisser–Greenhouse correction in B. All data represent the mean ± SEM. ^****^*P* < 0.0001.


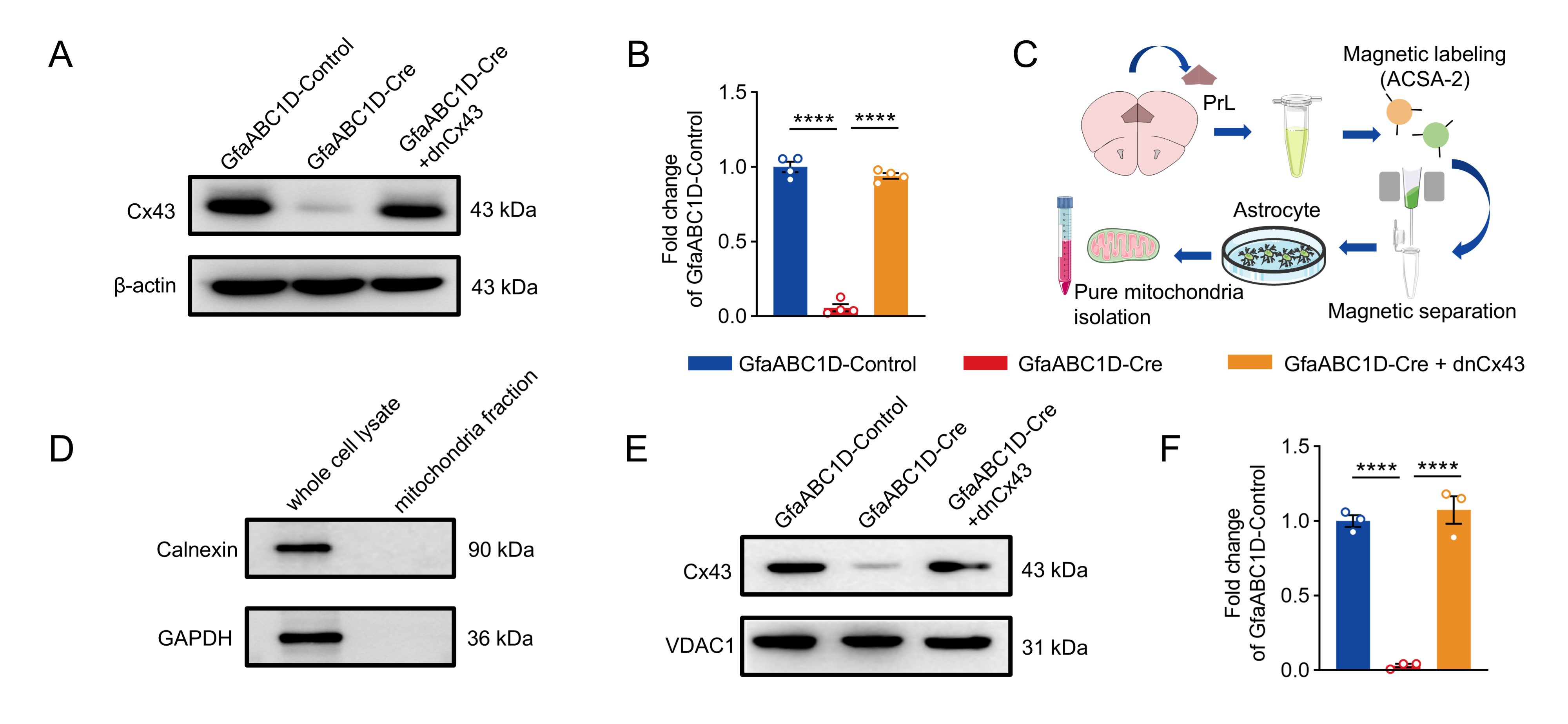


**Fig. S4.** Validation of Cx43 and dnCx43 expression in the PrL. (A, B) Validation of Cx43 expression in the PrL using Western blotting. Representative immunoblots of Cx43 (A) and their quantitative analysis (B) n = 4. (C) Schematic of the pure mitochondria isolation procedure from astrocytes of PrL. (D) Confirming the purity of the isolated mitochondrial fraction by Western blot. Calnexin and GAPDH were used as markers for the endoplasmic reticulum and cytosol respectively. (E) Representative immune blots for the endogenous and exogenous of Cx43 in mitochondrial fractions. (F) Quantitative analysis of Cx43 expression in astrocytic mitochondria. n = 3. One-way ANOVA followed by Dunnett's multiple comparisons test was performed. All data represent the mean ± SEM. ^****^*P* < 0.0001.


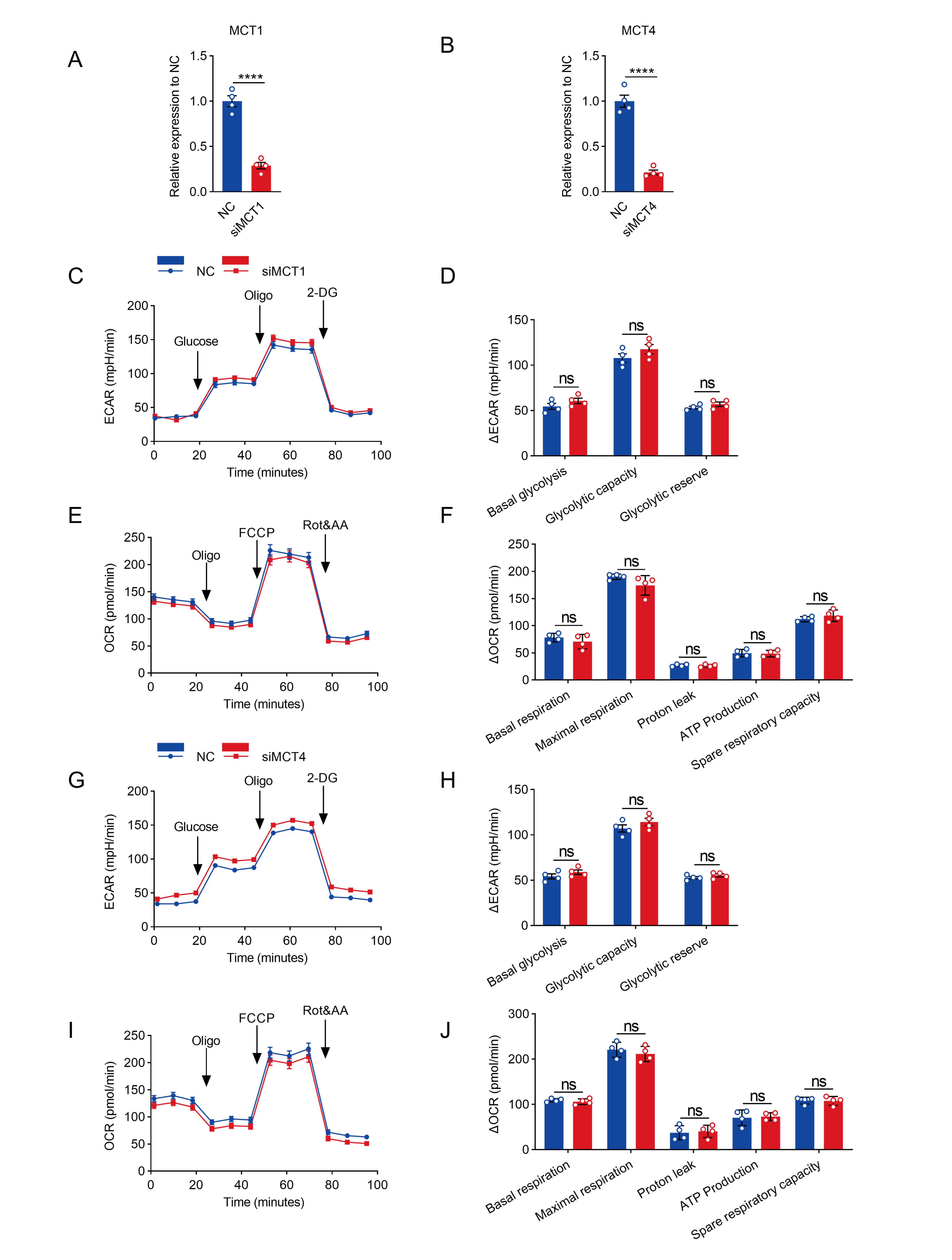


**Fig. S5.** Knockdown of MCT1 or MCT4 does not affect the metabolic activity of astrocytes. (A, B) The level of mRNA expression was tested using qPCR. n = 4. (C, G) The real-time changes of ECAR traces in MCT1-knockdown (C) or MCT4-knockdown (G) astrocytes. (D, H) Quantification of basal glycolysis, glycolytic capacity and glycolytic reserve in MCT1-knockdown (D) or MCT4-knockdown (H) astrocytes. n = 4. (E, I) The real-time changes of OCR traces in MCT1-knockdown (E) or MCT4-knockdown (I) astrocytes. (F, J) Quantification of basal respiration, maximal respiration, proton leak, ATP production and spare respiratory capacity in MCT1-knockdown (F) or MCT4-knockdown (J) astrocytes. n = 4. Two-tailed Student’s *t*-test was performed between two groups. All data represent the mean ± SEM. ns, no significant difference.


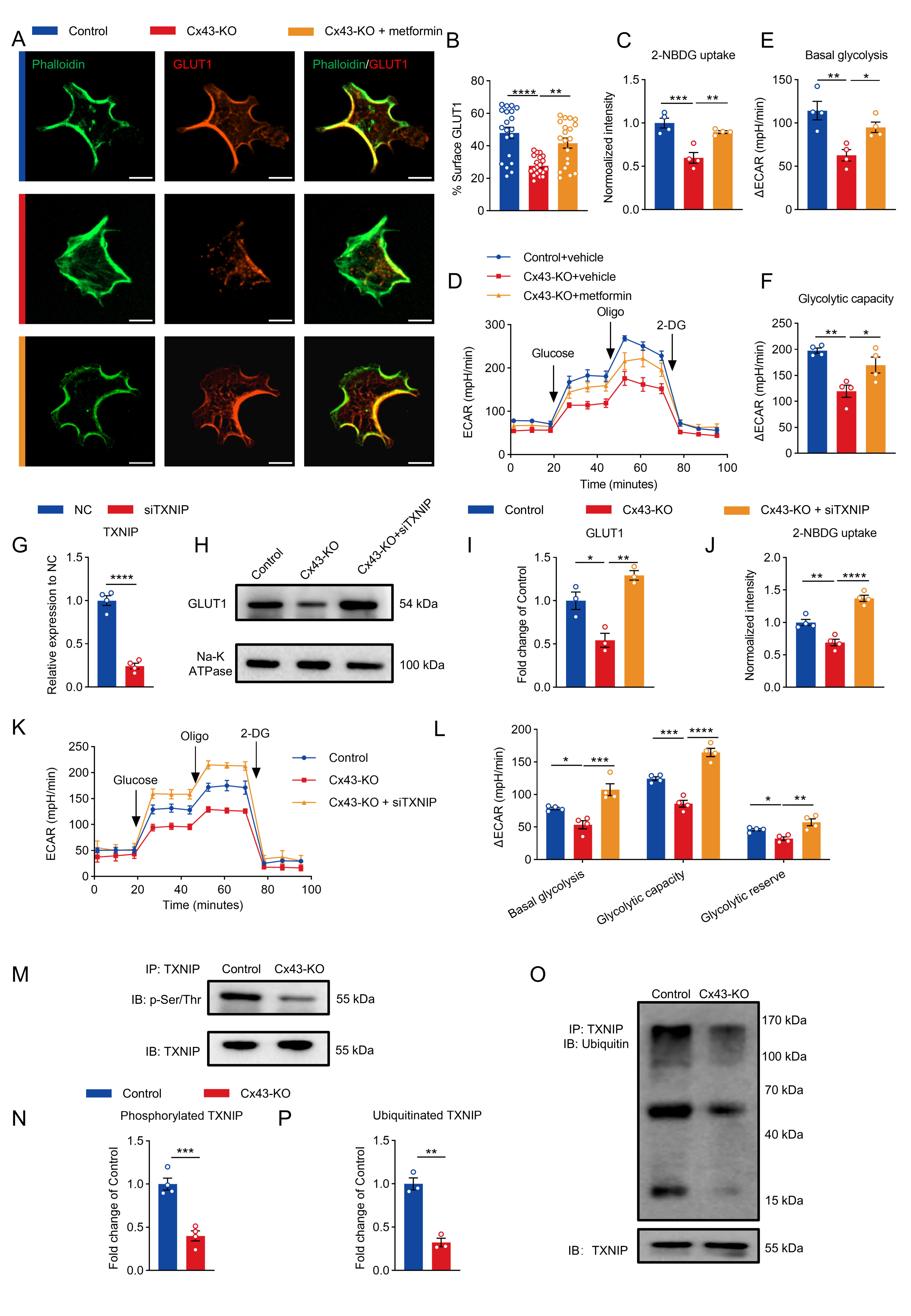


**Fig. S6.** Cx43 regulates astrocyte glycolysis via the AMPK–TXNIP–GLUT1 signaling pathway. (A, B) Representative images of immunofluorescence staining (A) and quantification (B) of cell surface GLUT1 in Control and Cx43-KO astrocytes. Scale bar, 20 μm. n = 20 cells. (C) Glucose uptake ability assessed by 2-NBDG uptake. n = 4. (D) The real-time changes of ECAR trace in astrocytes treated with or without metformin. (E, F) Quantification of basal glycolysis and glycolytic capacity. n = 4. (G) qPCR-based quantification of TXNIP knockdown efficiency. n = 4. (H–I) Representative immunoblots and quantification of plasma membrane GLUT1 expression. n = 3. (J) Glucose uptake ability assessed by 2-NBDG uptake. n = 4. (K) The real-time changes of ECAR trace in astrocytes. (L) Quantification of basal glycolysis, glycolytic capacity and glycolytic reserve. n = 4. (M) Representative Western blot bands of phosphorylated TXNIP. (N) Quantification of phosphorylated TXNIP. n = 4. (O) Representative Western blot bands of ubiquitinated TXNIP. (P) Quantification of ubiquitinated TXNIP. n = 3. One-way ANOVA followed by Dunnett's multiple comparisons test was performed in multiple groups; two-tailed Student’s *t*-test was performed between two groups. All data represent the mean ± SEM. ^*^*P* < 0.05, ^**^*P* < 0.01, ^***^*P* < 0.001, ^****^*P* < 0.0001.


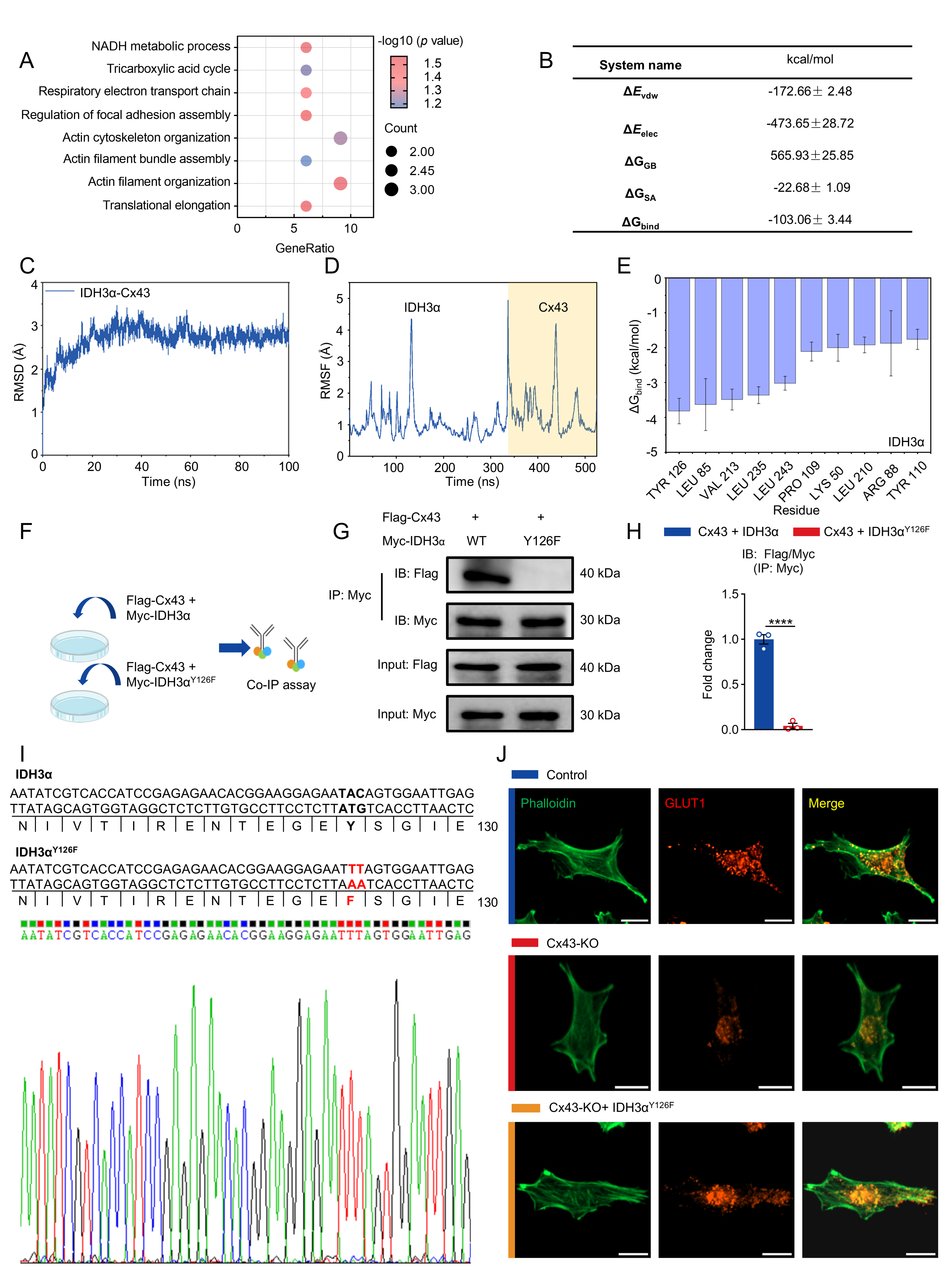


**Fig. S7.** The interaction between Cx43 and IDH3α regulates glucose uptake. (A) GO enrichment analysis of differential interacting proteins. (B) Binding free energies and energy components predicted by MM/GBSA (kcal/mol). (C) The temporal evolution of the root mean square deviation (RMSD) of the complex during molecular dynamics simulations. (D) Root mean square fluctuation (RMSF) calculated based on molecular dynamics simulation trajectories. (E) Top 10 amino acids contributing to protein-protein binding using the energy decomposition technique of MM-GBSA. (F) Schematic of the experimental workflow. (G–H) Representative Western blot bands and quantification of co-immunoprecipitation analyses assessing the interaction between Flag and Myc. n = 3. (I) Sequencing results of the IDH3α^Y126F^ mutant plasmid. (J) Representative images of immunofluorescence staining of cell surface GLUT1. Scale bar, 20 μm. Two-tailed Student’s *t*-test was performed between two groups. All data represent the mean ± SEM. ^****^*P* < 0.0001.


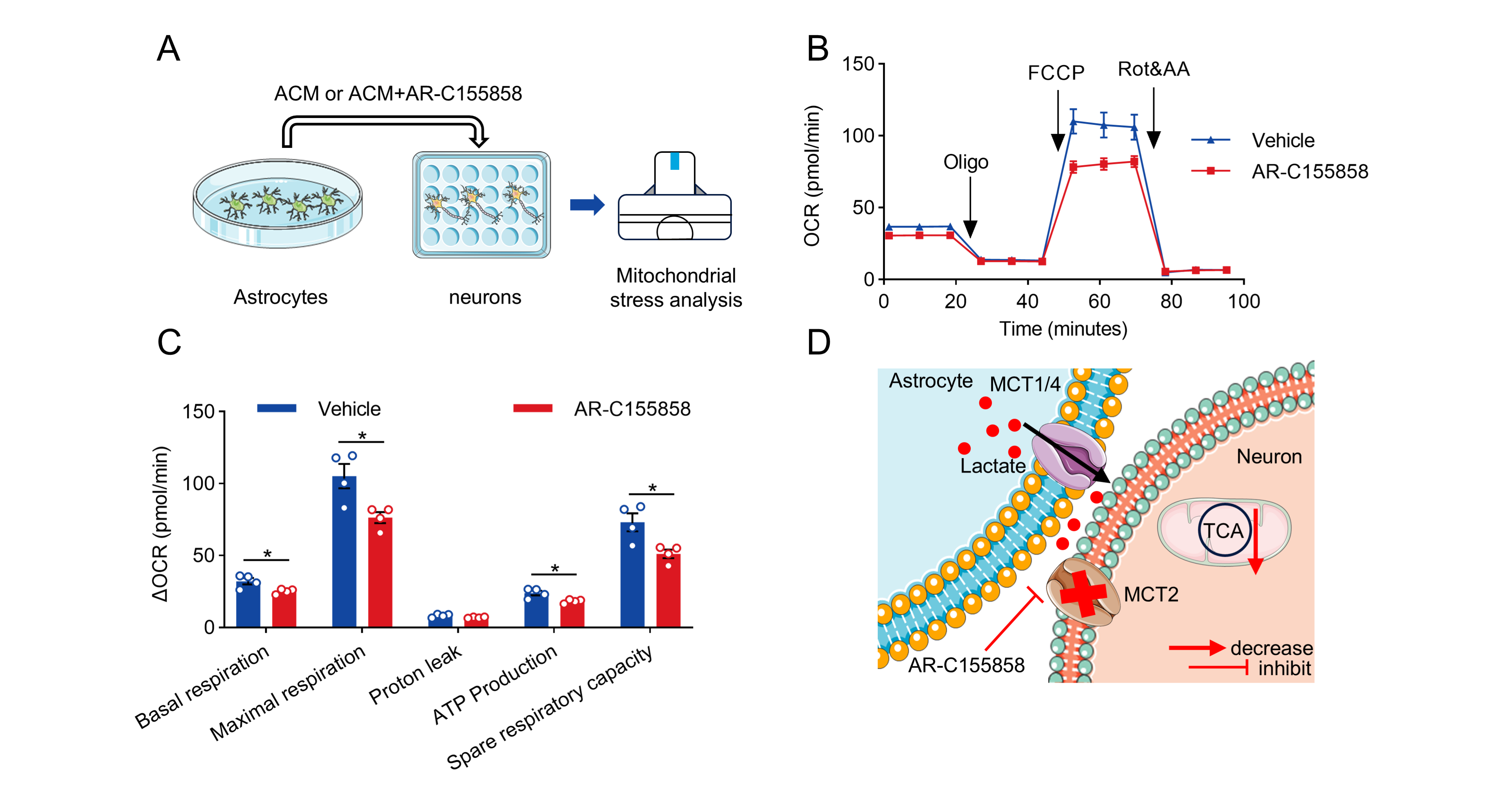
 **Fig. S8.** Inhibition of neuronal MCT2 abolishes neuronal OXPHOS. (A, B) The schematic diagram of WT mouse primary neurons treated with ACM from astrocytes with or without AR-C155858 (100 nM, a MCT2 inhibitor). (C) Quantification of basal respiration, maximal respiration, proton leak, ATP production and spare respiratory capacity. n = 4. (D) The mechanism diagram of AR-C155858 treatment. Two-tailed Student’s *t*-test was performed between two groups. All data represent the mean ± SEM. ^*^*P* < 0.05.


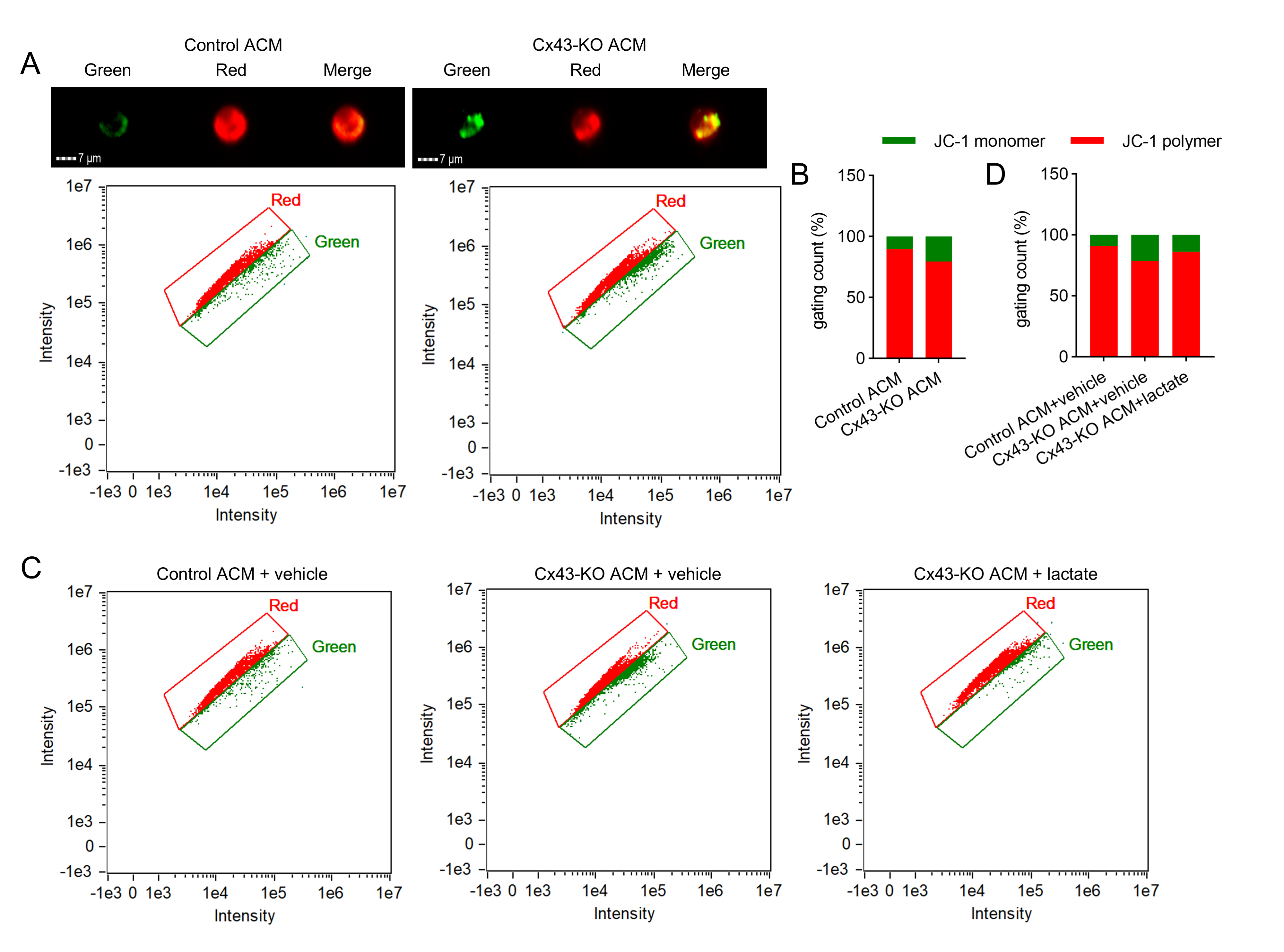


**Fig. S9.** Lactate restores decreased mitochondrial membrane potential induced by Cx43-KO astrocytes ACM. (A) Representative images in the JC-1 staining. Scale bar = 7 μm. (B, D) The ratio of JC-1 monomers/polymers in neurons. n = 4. (C) Representative flow cytometry results in the JC-1 staining.


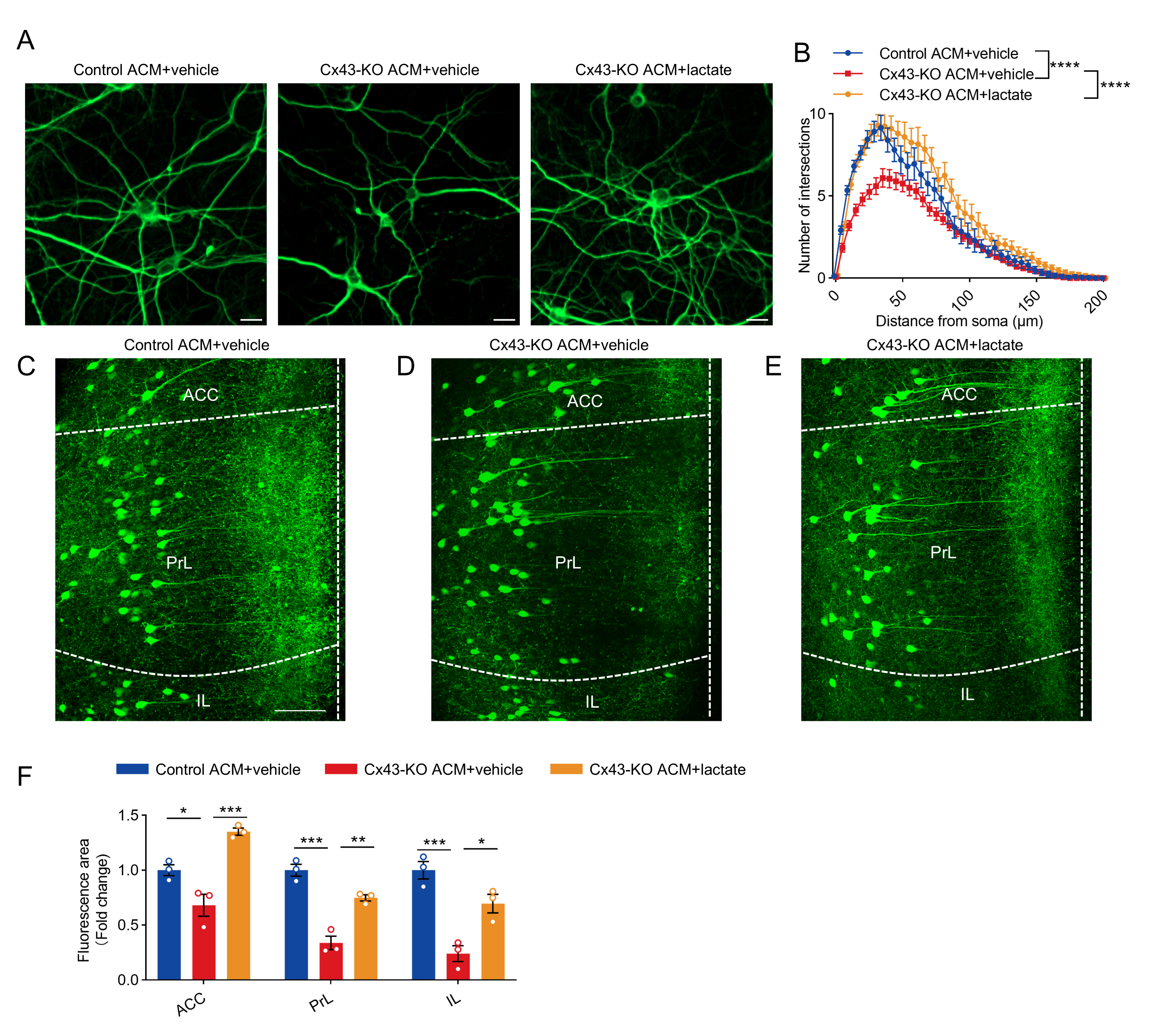


**Fig. S10.** Lactate significantly ameliorates the reduction in branching complexity of neurons induced by Cx43-KO ACM. (A) MAP2 staining of ACM-treated neurons. Scale bar, 20 μm. (B) Sholl analysis of dendritic branches. n = 20 neurons. Representative two-photon imaging images (C–E) and statistical analysis (F) of the area of GFP fluorescence in the terminal dendrites of three brain regions. Scale bar, 50 μm. n = 3. Two-way ANOVA followed by the Geisser–Greenhouse correction in B; one-way ANOVA followed by Dunnett's multiple comparisons test in multiple groups. All data represent the mean ± SEM. ^*^*P* < 0.05, ^**^*P* < 0.01, ^***^*P* < 0.001, ^****^*P* < 0.0001.


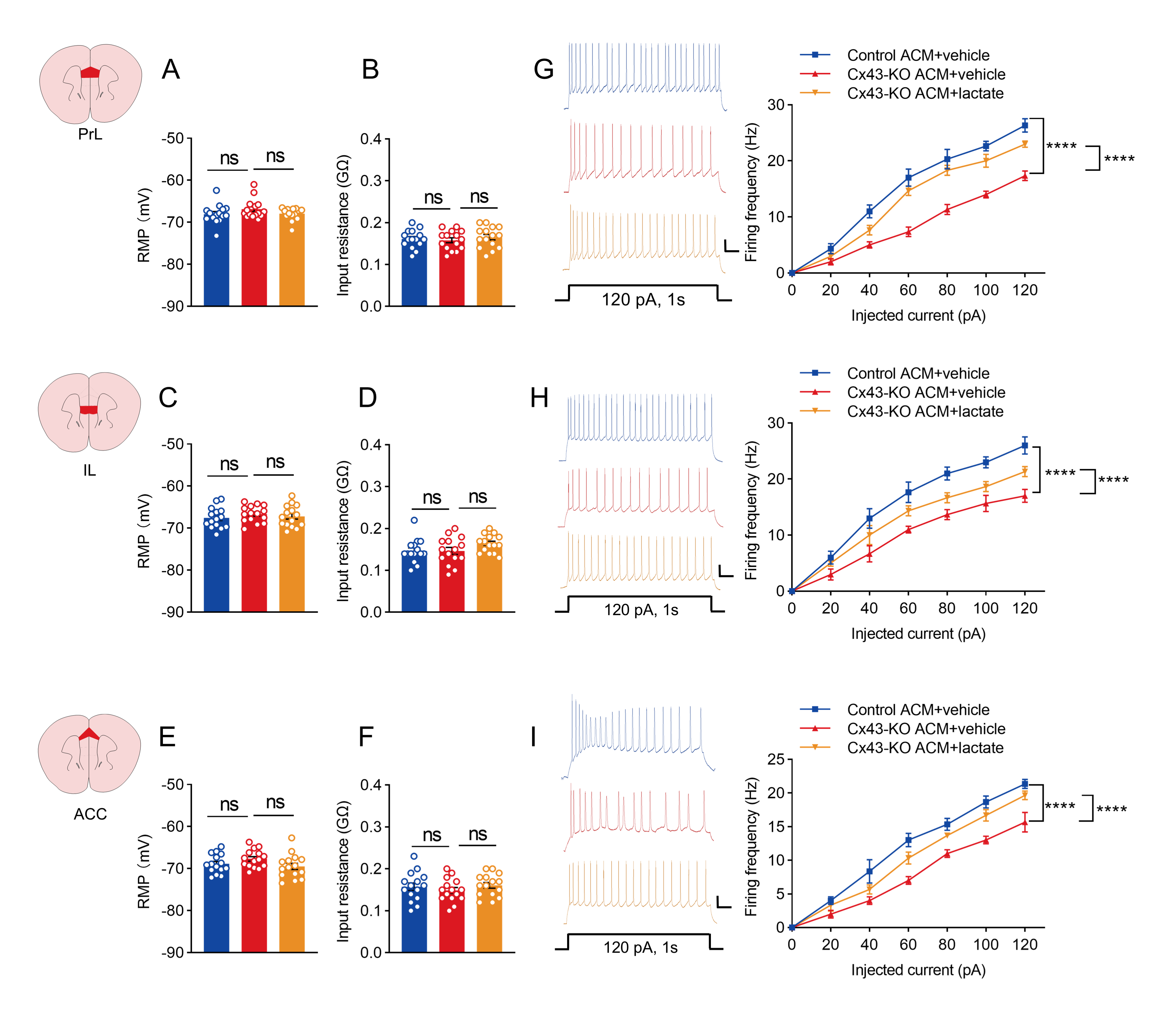


**Fig. S11.** Lactate enhances the excitability of pyramidal neurons in the mPFC. (A–F) The resting membrane potential (A, C, E) and input resistance (B, D, F) of pyramidal neurons in the PrL, IL and ACC. (G–I) Neuronal excitability in the PrL (G), IL (H) and ACC (I) was measured by injecting a series of depolarizing pulses. Left, representative firing traces. n = 15 neurons from 3 mice per group. Scale bars: 0.1 s, 20 mV. One-way ANOVA followed by Dunnett's multiple comparisons test in multiple groups; two-way ANOVA followed by the Geisser–Greenhouse correction in G–I. All data represent the mean ± SEM. ^****^*P* < 0.0001. ns, no significant difference.


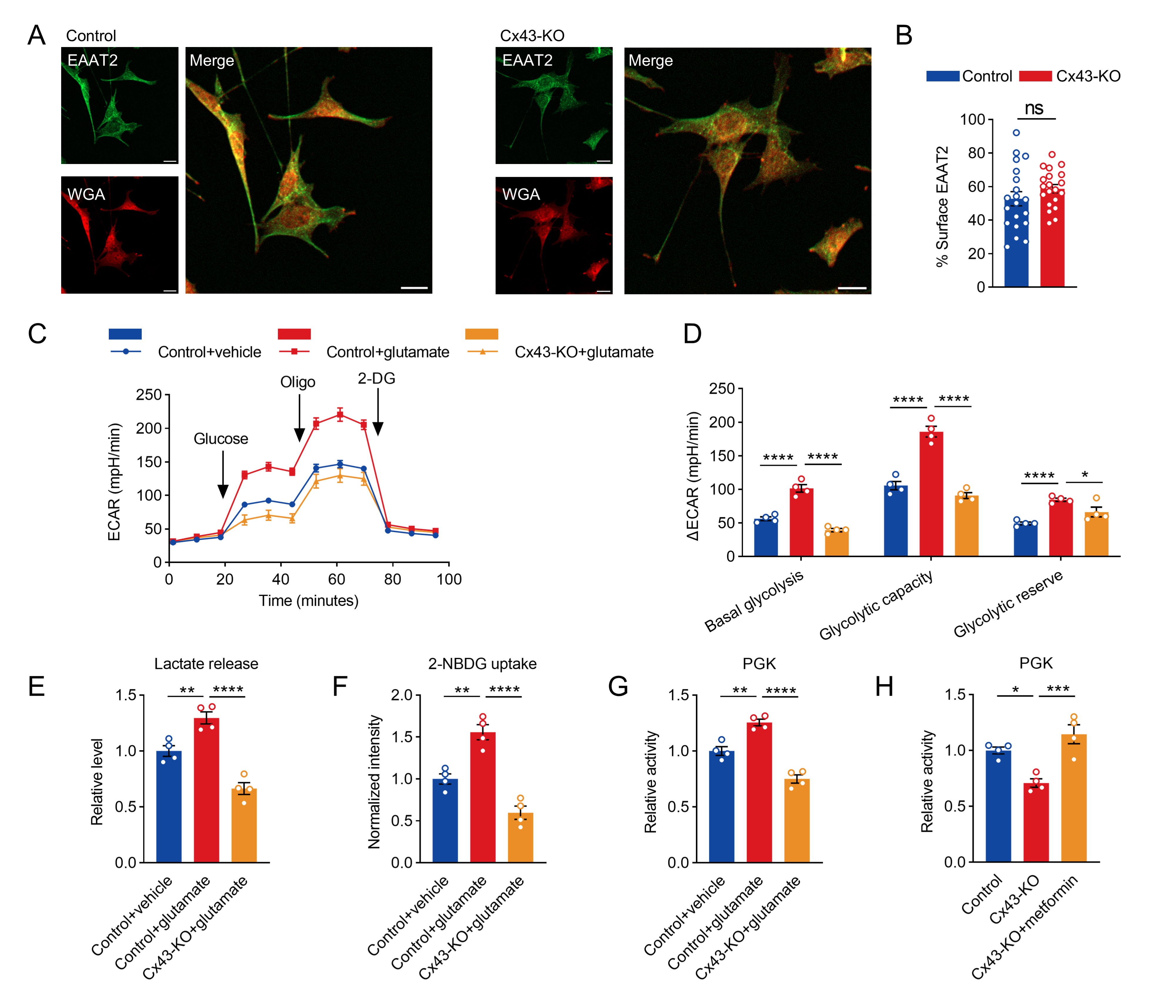


**Fig. S12.** The absence of Cx43 suppresses the elevation of glycolysis driven by glutamate. (A) Representative images of immunofluorescence staining and (B) quantification of cell surface EAAT2 in Control and Cx43-KO astrocytes. Scale bar, 20 μm. n = 20 cells. (C) The real-time changes of ECAR traces in astrocytes. (D) Quantification of basal glycolysis, glycolytic capacity and glycolytic reserve. n = 4. (E) Quantification of lactate release from astrocytes. n = 4. (F) Glucose uptake ability assessed by 2-NBDG uptake. n = 4. (G) Variations in PGK enzymatic activity in response to glutamate stimulation. (H) Changes in PGK enzymatic activity following treatment with metformin. Two-tailed Student’s *t*-test was performed between two groups; one-way ANOVA followed by Dunnett's multiple comparisons test in multiple groups. All data represent the mean ± SEM. ns, no significant difference. ^*^*P* < 0.05, ^**^*P* < 0.01, ^***^*P* < 0.001, ^****^*P* < 0.0001. ns, no significant difference.

**2. Supplementary table**

**Table S1.** The primer sequences

| Primer | Forward primer (5' to 3') | Reverse primer (5' to 3') |
| --- | --- | --- |
| *Actb* | GTGACGTTGACATCCGTAAAGA | GCCGGACTCATCGTACTCC |
| *C3* | CCAGCTCCCCATTAGCTCTG | GCACTTGCCTCTTTAGGAAGTC |
| *H2-t23* | GGACCGCGAATGACATAGC | GCACCTCAGGGTGACTTCAT |
| *Serping1* | ACAGCCCCCTCTGAATTCTT | GCTGAGAAGGCGTGGTAGAG |
| *H2-D1* | TCCGAGATTGTAAAGCGTGAAGA | ACAGGGCAGTGCAGGGATAG |
| *Ggta1* | GTGAACAGCATGAGGGGTTT | GTTTTGTTGCCTCTGGGTGT |
| *Iigp1* | GGGGCAATAGCTCATTGGTA | ACCTCGAAGACATCCCCTTT |
| *Gpb2* | CTGCACTATGTGACGGAGCTA | CGGAATCGTCTACCCCACTC |
| *Fbln5* | GCTTGTCGTGGGGACATGAT | TGGGGTAGTTGGAAGCTGGTA |
| *Ugt1a* | CCTATGGGTCACTTGCCACT | AAAACCATGTTGGGCATGAT |
| *Fkbp5* | TGAGGGCACCAGTAACAATGG | CAACATCCCTTTGTAGTGGACAT |
| *Psmb8* | CAGTCCTGAAGAGGCCTACG | CACTTTCACCCAACCGTCTT |
| *Srgn* | GCAAGGTTATCCTGCTCGGA | TGGGAGGGCCGATGTTATTG |
| *Clcf1* | CTTCAATCCTCCTCGACTGG | TACGTCGGAGTTCAGCTGTG |
| *Tgm1* | CTGTTGGTCCCGTCCCAAA | GGACCTTCCATTGTGCCTGG |
| *Ptx3* | CCTGCGATCCTGCTTTGTG | GGTGGGATGAAGTCCATTGTC |
| *S100a10* | CCTCTGGCTGTGGACAAAAT | CTGCTCACAAGAAGCAGTGG |
| *Sphk1* | GATGCATGAGGTGGTGAATG | TGCTCGTACCCAGCATAGTG |
| *Cd109* | CACAGTCGGGAGCCCTAAAG | GCAGCGATTTCGATGTCCAC |
| *Ptgs2* | GCTGTACAAGCAGTGGCAAA | CCCCAAAGATAGCATCTGGA |
| *Emp1* | TTGGTGCTACTGGCTGGTCT | CATTGCCGTAGGACAGGGAG |
| *Slc10a6* | GCTTCGGTGGTATGATGCTT | CCACAGGCTTTTCTGGTGAT |
| *Tm4sf1* | GCCCAAGCATATTGTGGAGT | AGGGTAGGATGTGGCACAAG |
| *Lcn2* | CCAGTTCGCCATGGTATTTT | CACACTCACCACCCATTCAG |
| *Steap4* | CCCGAATCGTGTCTTTCCTA | GGCCTGAGTAATGGTTGCAT |
| *S1pr3* | AAGCCTAGCGGGAGAGAAAC | CAGGGAACAATTGGGAGAGT |
| *Timp1* | GCAACTCGGACCTGGTCATAA | CGGCCCGTGATGAGAAACT |
| *Hspb1* | GACATGAGCAGTCGGATTGA | GGATGGGGTGTAGGGGTACT |
| *Cxcl10* | CCAAGTGCTGCCGTCATTTTC | GGCTCGCAGGGATGATTTCAA |
| *Cd44* | CACCATTGCCTCAACTGTGC | TTGTGGGCTCCTGAGTCTGA |
| *Osmr* | GTGAAGGACCCAAAGCATGT | GCCTAATACCTGGTGCGTGT |
| *Cp* | TGTGATGGGAATGGGCAATGA | AGTGTATAGAGGATGTTCCAGGTCA |
| *Serpinga3n* | CCTGGAGGATGTCCTTTCAA | AGGGGGCAATTTCAGTTTCT |
| *Aspg* | CTGACCAGAGGATCATCTACACG | CTCTATGGTTTGGGCAATCTGAA |
| *Vim*  *Slc16a1*  *Slc16a3*  *Txnip* | AGACCAGAGATGGACAGGTGA  GACCATTGTGGAATGCTGCCCT  TCCATCCTGCTGGCTATGCTCT  CATTATCTCAGGGACTTGCG | TTGCGCTCCTGAAAAACTGC  CGATGATGAGGATCACGCCACA  CAGAAGGACGCAGCCACCATTC  CTCACTGCACGTTGCTGT |

**3. Supplementary methods**

**3.1. Construction of Cx43^flox/flox^ gene-modified mice**

Male Cx43^flox/flox^ gene-modified mice, aged 6–8 weeks, were generated using the CRISPR/Cas9 system at the Institute of Laboratory Animal Sciences, Chinese Academy of Medical Sciences and Peking Union Medical College. A sgRNA-target sequence was chosen from the second coding exon of the Cx43 gene, and T7 promoter-containing sgRNA PCR products were amplified from pUC57-sgRNA expression vector (Addgene ID: 51132) using the following oligos: m-Cx43-gRNA UP1:5'-TAGGGTAATAGCACCCAGATAC-3' and m-Cx43-gRNA DOWN1:5'-AAACGTATCTGGGTGCTATTAC-3,' targeting site 1: CCTGTATCTGGGTGCTATTAC, m-Cx43-gRNA UP2:5'-TAGGCAAAAGTAGGGAAAGGGG-3', and m-Cx43-gRNA DOWN2 5'-AAACCCCCTTTCCCTACTTTTG-3', targeting site 2: CAAAAGTAGGGAAAGGGGAGG. The resulting product was gel-purified and used as a template for in vitro transcription using a MEGA short-script T7 kit (Life Technologies, AM1354). Cas9 messenger RNA (mRNA) was transcribed *in vitro* from the linearized pST1374-NLS-flag-linker-Cas9 (Addgene ID: 44758) using a T7 ULTRA kit. Subsequently, the Cas9 mRNA, sgRNAs, and Cx43 floxing donors were purified and combined for injection into C57BL/6 mouse fertilized eggs. The injected embryos were then transferred into the fallopian tubes of pseudopregnant females to generate chimeric mice. Germline transmission and genotyping were verified using polymerase chain reaction (PCR). The PCR products were gel-purified and used for TA cloning, followed by sequencing.

**3.2. Chronic social defeat stress (CSDS)** **model**

The CSDS mouse model was established as previously described [1]. Throughout the training phase, each mouse experienced daily interactions with an aggressive CD-1 mouse for 5-10 minutes each day over a span of ten days. After each encounter, the subject mouse remained in the aggressor's home cage, separated by a perforated translucent plastic divider. In contrast, control mice were housed in pairs with the same divider but did not have any exposure to CD-1 mice; instead, they were moved daily to a cage with a novel control mouse. A social interaction test was performed 24 hours after the last social defeat session.

**3.3. Behavioral tests**

All behavioral experiments were conducted using a double-blind procedure. No sample size estimation was performed for this study.

The social interaction test was conducted to assess social behavior in adult male C57BL/6 mice. Each mouse was placed in an open field arena (50 cm × 50 cm × 50 cm) containing an empty enclosure. The behavior of C57BL/6 mice was assessed in two conditions: first, in the absence of social targets, where a single mouse was placed in a corner of an open field with a transparent cage for 2.5 minutes. In the second condition, an unfamiliar CD-1 mouse was introduced into the cage, and the same C57BL/6 mouse was monitored in the open field for another 2.5 minutes. The social interaction (SI) ratio is calculated by dividing the time the C57BL/6 mouse spends in the interaction area with the unfamiliar CD-1 mouse by the time spent in the interaction area without the CD-1 mouse. Mice exhibiting an SI greater than 1 were classified as resistant, while those with an SI less than 1 were classified as susceptible. Susceptible mice were considered indicative of successful modeling and were utilized for subsequent experiments.

In the TST, mice were suspended 10 cm above the surface, with their tails securely attached to a horizontal pole. The test lasted for 6 min, during which immobility was observed and recorded for the final 4 min. Immobility was defined as the complete absence of any form of movement.

In the FST, mice were placed in a transparent cylinder measuring 18 cm in diameter and 58 cm in height, filled with water at 23 ± 1 °C. The mice were monitored for 6 min, and the duration of immobility during the last 4 min was recorded.

The SPT followed a previously published protocol [2]. Briefly, the mice were given free access to water and 1% sucrose solution for 48 h. The mice were then housed individually and deprived of food and water for 24 h before the test. The mice were given bottles of 1% sucrose solution and water for 12 h. The positions of the bottles were changed after 6 h. The amounts of sucrose and water consumed were recorded.

**3.4. *In vivo* ^1^H** **MRS data acquisition and processing**

^1^H MRS datasets were acquired using a PharmaScan 70/16 scanner (Bruker, USA). Data was acquired using the ParaVision 6.0.1 software (Bruker, USA). Prior to scanning, the animals were continuously anesthetized with 2% isoflurane in a mixture of 5/95% CO_2_/O_2_ gas to maintain stable positioning. The head was immobilized using a specialized animal cradle with tooth and ear strips to minimize the motion artifacts during scanning. The isoflurane concentration was reduced to 1.5% at the beginning of the scan. A FieldMap and continuous local shimming were used for each MRI examination to optimize the magnetic field uniformity. T2-weighted TurboRARE sequences were used as anatomical reference scans. A region of interest (ROI) measuring 2.5 mm × 1 mm × 2 mm, selected from the T2 scan and corresponding to the bilateral PrL, was used for ^1^H MRS. The PRESS sequence was employed with the following parameters: The repetition time (TR) = 2500 ms, the echo time (TE) = 16.2 ms, 400 data points sampled, and a total acquisition time of 16 min and 40 s. A combination of outer volume and water suppression techniques were applied. The NMR spectra of the neurochemicals were obtained using Topspin 4.2.0 software. Metabolites were quantified using the LCModel, with relative levels normalized to creatine levels.

**3.5. Electrophysiological recording and processing**

For *In vivo* electrophysiological recording, the mice were initially anesthetized with 2% isoflurane and a 5/95% CO_2_/O_2_ gas mixture and placed in a stereotaxic frame (RWD Life Science, China). A skin flap measuring approximately 1.5 cm^2^ was carefully retracted from the skull. A 16-channel electrode (Bio-Signal Technologies, China) was inserted perpendicular to the PrL. The recording coordinates from bregma were as follows: AP, +2.34 mm; DV, –2 mm; ML, –0.25 mm. The reference and ground electrodes were wrapped around a screw, and dental acrylic was applied to secure the headplate. The animals were given a recovery period of 7 days before the recording session. The recorded responses were amplified and filtered using a Cerebus 16-channel system (Blackrock Microsystems, USA). Spiking signals were sampled at 30 kHz, and spike waveforms were detected by band-pass filtering the signals within the range of 250–5000 Hz, with a threshold value of −40 μV. Putative pyramidal neurons were identified and isolated from the background noise based on waveforms, interspike intervals, and principal component clustering. The activity of each neuron was continuously monitored for approximately 10 min, and the processed spike signals were imported into NeuroExplorer (Plexon Inc., USA) for analysis.

For brain slice electrophysiology, we employed a modified version of established techniques [1]. Briefly, adult male mice were subjected to isoflurane anesthesia, after which their brains were rapidly removed and placed in ice-cold artificial cerebrospinal fluid (ACSF). Coronal slices (300 µm) containing the medial prefrontal cortex (mPFC) were prepared in ice-cold ACSF using a vibratome (Leica, Germany) and subsequently moved to an incubation chamber containing ACSF maintained at 32 °C for 30 minutes. In lactate supplementation experiments, brain slices were incubated in solution containing ACM and 10 mM sodium lactate at 32 °C for 2 hours prior to electrophysiological recording. All solution was continuously saturated with a gas mixture of 95% O_2_ and 5% CO_2_ (vol/vol).

During recordings, the slices were placed in a recording chamber that was continuously superfused with ACSF at a rate of 2 ml/min. Whole-cell patch-clamp recordings were performed on pyramidal neurons located in the three subregions of the mPFC, utilizing infrared optics with an upright microscope and an infrared-sensitive CCD camera for visualization. Pipettes were fabricated using a micropipette puller (Sutter Instrument, USA) to achieve resistances between 3 and 5 MΩ. Recording was carried out with a MultiClamp 700B amplifier and a 1440A digitizer (Molecular Devices, USA). The excitability of pyramidal neurons was assessed by applying a series of depolarizing current pulses from 0 to 120 pA in 20 pA increments.

**3.6.** **fMRI data acquisition and processing**

MRI datasets were generated using a PharmaScan 70/16 machine (Bruker, USA) and acquired with ParaVision 6.0.1 software (Bruker, USA). During the scan, animals were anesthetized with a continuous flow of 2% isoflurane mixed with 5/95% CO_2_/O_2_ gas mixture to maintain stable positioning. The animal head was immobilized using a specialized cradle equipped with tooth and ear strips to minimize motion artifacts. The isoflurane concentration was reduced to 1.5% at the beginning of the scan. Before each MRI examination, a FieldMap and continuous local shimming were implemented to enhance the magnetic field uniformity. Subsequently, T2-weighted TurboRARE sequences were used as anatomical reference scans. Functional MRI was performed using a free-induction decay echo-planar imaging (FID-EPI) sequence with 300 repetitions. The imaging parameters included a field of view (FOV) of 20 × 20 mm^2^, 40 sections with a thickness of 0.35 mm at 0.05 mm intervals, an image size of 64 × 64, a TR of 2000 ms, and a TE of 15 ms. The functional brain data was processed as follows: first, the original data was converted to nifti format files using dcm2nii (2MAY2016) software with ten-fold voxel magnification, and the middle layer was selected as the reference layer for temporal alignment. The temporally processed volumes of each subject were realigned to the mean volume to correct for head motion, and a mean image was created from 300 realigned volumes. After cephalometric correction, all subject data were registered in the standard space. Subsequently, the functional images were registered to the Turone Mouse Brain Template and Atlas (TMBTA) (https://www.nitrc.org/projects/tmbta_2019) through co-registration with the T2 and average function images. Gaussian smoothing (FWHM = 6 mm) and filtering with a 0.01–0.08 Hz range were applied to the registered data using SPM12. After regression covariate analysis and detrending, the preprocessed data were analyzed. The ALFF and ReHo results were calculated using the Resting State fMRI data analysis Toolkit (REST) [3]. The normalized ALFF and ReHo data were subjected to a two-sample *t*-test using SPM12 for statistical analysis. Significant amplitude of ALFF and ReHo values between the chosen groups were obtained based on a voxel-level height threshold of *p* < 0.05 and a cluster-extent threshold of 20 voxels. The whole brain was divided into 258 seed points, and the correlation coefficients between these points were computed to evaluate functional connectivity among different brain regions. A total of 258 brain regions were divided into eight regions based on their locations: cortical, visual, somato, olfactory, midbrain, subcortical, limbic, and hindbrain. These correlation coefficients were transformed using Fisher-z standardization to obtain zFC values using DPABI (v3.1) [4]. Statistical analysis was performed using MATLAB R2013b (MathWorks, USA) and GRETNA (v2.0.0) [5]. The functional connectivity results were visualized using Circos (v0.69) and BrainNet Viewer (v1.61) [6, 7]. The localization of brain regions was determined based on the atlas.

**3.7. Luminex liquid suspension chip detection**

The Bio-Plex Pro Mouse Cytokine Grp I Panel 23-plex was used following the manufacturer's instructions by Wayen Biotechnologies (Shanghai). Briefly, GfaABC1D-Control or GfaABC1D-Cre mouse PrL lysates were incubated in 96-well plates containing microbeads for 30 min, followed by a 30-minute incubation with the detection antibody. Subsequently, streptavidin-PE was added to each well for 10 min, and measurements were obtained using a Luminex 200 system (Luminex Corporation, USA).

**3.8. Isolation of astrocytes from adult mouse brain**

Isolation of astrocytes from adult mouse brain was performed as previously described [8]. In brief, adult mice were deeply anesthetized and then perfused with phosphate-buffered saline (PBS). The prefrontal cortex was quickly removed and washed with pre-chilled PBS. The brain tissue was dissociated using both mechanical and enzymatic methods, following the protocol provided with the adult brain dissociation kit (RWD Life Science, China). After myelin removal, the resulting cell pellet was resuspended in PBS containing 0.5% bovine serum albumin (BSA) for the isolation of specific cell types. Astrocytes were isolated using anti-ACSA-2 microbeads (Miltenyi Biotec, Germany) with a MACS MultiStand Separator (Miltenyi Biotec, Germany), adhering to the manufacturer’s guidelines. The obtained astrocytes were cultured in a high-glucose DMEM/F12 medium (Thermo Fisher Scientific, USA) consisting of 10% fetal bovine serum (Thermo Fisher Scientific, USA) and 1% penicillin/streptomycin.

**3.9 Pure mitochondria isolation**

Cellular mitochondrial isolation was performed as previously described [9]. In brief, crude mitochondrial fractions were obtained by differential centrifugation. Cells were washed in Ca2^+^/Mg2^+^-free PBS, trypsinized, homogenized, and subjected to sequential low-speed spins to remove unbroken cells and nuclei (5 min at 600 × g). The resulting supernatant was centrifuged at 7000 × g for 10 min to pellet crude mitochondria; the pellet was washed, re-pelleted at 7000 × g, and clarified at 10000 × g to yield the crude mitochondrial fraction. For further purification, the crude mitochondrial pellet was resuspended in MRB buffer and layered onto a Percoll gradient. Following centrifugation at 95000 × g for 30 min, mitochondrial bands were collected, diluted and washed by centrifugation at 6300 × g for 10 min. The washed pellet (pure mitochondria) was recovered and, where indicated, further enriched by an additional 100000 × g spin to isolate the mitochondrial-associated membrane fraction. All steps were performed at 4 °C in the presence of protease and phosphatase inhibitors.

**3.10. Cell treatment**

For plasmid transfection, dnCx43(T154A) plasmid and mito-Cx43 plasmid were constructed as previously described [10, 11]. dnCx43(T154A) plasmid, Myc-IDH3α^Y126F^ plasmid, Myc-IDH3α plasmid, Flag-Cx43 plasmid, and mito-Cx43 plasmid were constructed by OBiO Technology (Shanghai, China) and Genomeditech (Shanghai, China) and transfected using TransIntro EL Transfection Reagent (TransGen Biotech, China) according to the manufacturer's instructions.

For the pharmacological treatment of astrocytes, 2.5 mM metformin (yuanye Bio-Technology, China) was added in the media for 24 h. For glutamate stimulation, astrocytes were treated with 200 μM glutamate (MCE, USA) for 1 hour. The culture supernatant of astrocytes was carefully aspirated after centrifugation at 1000 × g for 10 min, which was then referred to as the astrocyte conditional medium (ACM). ACM was collected as previously described [12].

For the transfection of siRNA, the INTERFERin transfection reagent (Polyplus, France) was utilized according to the manufacturer's instructions. Scramble siRNA (NC) and siRNAs for MCT1, MCT4 and TXNIP were provided by OBiO Technology (Shanghai, China).

For neuronal drug treatment, 2 mM lactate (aladdin, China) was added to the ACM for 48 h, and 100 nM AR-C155858 (aladdin, China) was added for 24 h. Primary neurons were treated with ACM for 48 h before experiments.

**3.11. Measurements of the ECAR and the OCR**

The Seahorse XFe24 Analyzer (Agilent Technologies, USA) was used in accordance with the manufacturer's instructions. A glycolytic assay of the isolated tissues was conducted as previously described [1]. Mice were anesthetized with 1.5% isoflurane, followed by the rapid collection of brain tissue in ice-cold modified artificial cerebrospinal fluid (ACSF). Coronal brain slices encompassing the PrL (200 µm) were sliced using a vibratome (Leica, Germany). The tissue was microdissected and relocated to an incubation chamber with the same ACSF composition, excluding glucose, at room temperature for 30 min before conducting glycolytic assessments. The tissue was transferred into a 24-well Seahorse XF islet capture microplate (Agilent Technologies, USA) for subsequent assays. The measured data were normalized to the total protein content of the corresponding tissues. For the cellular assay, astrocytes and neurons were seeded at a density of 5 × 10^4^ cells per well in a 24-well Seahorse XF cell culture microplate (Agilent Technologies, USA) with culture medium. Prior to detection, the culture medium was rinsed and replaced with Seahorse XF DMEM (Agilent Technologies, USA). Cells were incubated in a CO_2_-deprived environment at 37 °C for 1 h. ECAR measurements were recorded prior to the addition of D-glucose (10 mM for astrocytes, 100 mM for isolated tissue), oligomycin (1 μM for astrocytes, 10 μM for isolated tissue), and 2-DG (50 mM). For mitochondrial stress measurements, the OCR was recorded before the addition of oligomycin (2.5 μM), FCCP (2 μM), and rotenone/antimycin A (0.5 μM). OCR measurements were conducted using inhibitors, including BPTES (3 μM), etomoxir (4 μM), and UK5099 (2 μM) to assess mitochondrial fuel dependency. OCR measurements were conducted using compounds including BPTES (3 μM), oligomycin (1.5 μM), FCCP (2 μM), and rotenone/antimycin A (0.5 μM) to analyze glutamine oxidation stress.

**3.12. Metabolic flux assay**

For glucose flux assay, astrocytes were cultured in glucose-free DMEM medium (Thermo Fisher Scientific, USA) supplemented with 2 g/L [U-^13^C_6_]-D-glucose (Energy Chemical, China), 10% FBS, and 1% penicillin/streptomycin for 24 h. For the glutamine flux assay, astrocytes were cultured in glutamine-free DMEM medium (Thermo Fisher Scientific, USA) supplemented with 4 mM [U-^13^C_5_]-glutamine (MCE, USA), 10% FBS, and 1% penicillin/streptomycin for 12 h. After incubation, the cells were washed with cold PBS, covered with liquid nitrogen, and cold methanol added. The cells were placed at −80 °C for 30 min. The cells were then collected in an 80% methanol-water solution and stored at −80 °C. Cells were obtained from frozen storage and mixed with 1 mL of an extraction reagent (methanol, acetonitrile, and water at a ratio of 4:4:2). The mixture was then treated with ultrasound for 1 min followed by a 1-minute pause, repeated for a total of five cycles in an ice water bath. After treatment, the sample was kept at −40 ℃ for 30 min and then centrifuged at 1500 × g for 15 min at 4 ℃. The supernatant was collected, dried under mild nitrogen gas, and reconstituted in a 50% acetonitrile aqueous solution (1:1, v/v) for ultra-high-performance liquid chromatography high-resolution mass spectrometry (UHPLC-HRMS) analysis. An equal volume of the solution was collected from each prepared sample and mixed to create a quality control (QC) sample. The samples were maintained at 8 ℃ in an automatic injector throughout the analysis process. Separation of the samples was achieved using an ACQUITY UPLC BEH Amide column (2.1 × 100 mm, 1.7 μm) (Waters, USA) with specific parameters including an injection volume of 2 μL, a column temperature of 30℃, and a flow rate of 0.4 mL/min. For positive ion mode, the gradient program was as follows: 0–0.5 min, B: 95%; 0.5–7 min, B: 95–65%; 7–8 min, B: 65–50%; 8–9 min, B: 50%; 9–9.1 min, B: 50–95%; 9.1–12 min, B: 95%. For negative-ion mode, the gradient program was the same as that for positive-ion mode. QC samples were included in the sample queue to ensure the stability and reliability of the system and the experimental data. Each sample was analyzed in the negative ion mode using electrospray ionization (ESI) and separated by UHPLC before being subjected to mass spectrometry analysis using a Thermo QE HF-X mass spectrometer (Thermo Fisher Scientific, USA). Mass spectrometry conditions: Ionization source: ESI ion source; Sheath gas flow rate: 30; Auxiliary gas: 10; Spray voltage: 2.5KV (+)/2.5KV (-); S-Lens RF: 50; Capillary temperature: 325 ℃; Auxiliary gas temperature: 300 ℃; Normalized collision energy (NCE): 30; Isolation window 1.5 m/z, Top N = 8. Scan range: 70–1050 m/z; scan mode: negative-ion scan. The acquired spectral data were analyzed using Xcalibur software 4.0.27.19 (Thermo Fisher Scientific, USA) to identify and quantify the target metabolites. Raw integrated area data were corrected using the natural isotope correction method as previously described [13].

**3.13. Enzyme activity, ATP, and lactate assays**

The enzyme activities of glutaminase (GLS), glutamate dehydrogenase (GDH), mitochondrial isocitrate dehydrogenase (mIDH), citrate synthase (CS), α-ketoglutarate dehydrogenase (α-KGDH) and phosphoglycerate kinase (PGK) in astrocytes were assessed using the GLS (Bioss, China), GDH (Abbkine, China), mIDH activity (Solarbio, China), CS activity (Solarbio, China) α-KGDH activity assay kits (Solarbio, China) and PGK activity assay kits (Solarbio, China) respectively as per the manufacturer's instructions. Following the manufacturer’s instructions ATP and lactate levels in astrocytes and or tissues were determined using the ATP assay kit (Beyotime, China) and the lactate assay kits (BestBio, China)

**3.14. Isolation of RNA and quantitative real-time polymerase chain reaction (qPCR)**

Total RNA was extracted using TRIzol reagent (ABclonal, China) and dissolved in 20 μL of DEPC-treated water (Beyotime, China) to obtain the total RNA. RNA was quantified using a NanoDrop 2000 spectrophotometer (Thermo Fisher Scientific, USA). cDNA was synthesized using cDNA Synthesis SuperMix for qPCR (TransGen Biotech, China). Specifically, 1 μg of RNA was combined with cDNA synthesis reagents, incubated at 42 °C for 15 min, and then at 85 °C for 5 s to obtain cDNA. cDNA was diluted with DEPC-treated water. A qPCR System (Bioer Technology, China) was used with a TransStart Tip Green qPCR Supermix kit (TransGen Biotech, China). The PCR amplification conditions were as follows: pre-denaturation at 94 °C for 30 s, followed by denaturation at 94 °C for 5 s, and annealing at 60 °C for 30 s for 40 cycles of extension. The primer sequences are listed in Table S1.

**3.15.** **Lucifer yellow diffusion assay**

For the *in vivo* assay, we administered Lucifer yellow (5 μg/μL, 2 μL) (Sigma-Aldrich, USA) dissolved in saline into the bilateral PrL (coordinates from bregma: AP, +2.34 mm; DV, –2 mm; ML, ±0.25 mm) using an infusion rate of 0.25 μL/min. Following a 30-minute period, the animals were euthanized. The brains were subsequently fixed in 4% paraformaldehyde for 48 hours, then dehydrated in 30% sucrose paraformaldehyde. Coronal brain sections (40 μm thick) were processed for immunofluorescence staining of GFAP, and the diffusion distance of fluorescein in GFAP-positive cells was quantified.

**3.16. Parachute assay**

Following previously described methods [14, 15], gap junctional coupling was assessed using the parachute assay. Briefly, astrocytes were seeded in 12‑well plates and cultured until >90% confluence. In one well, culture medium was removed and cells were washed once with PBS, then incubated with 0.5 mL loading solution (0.5 mL serum‑free medium containing 1 μL Calcein‑AM (Beyotime, China) and 1 μL CM‑DiI (Thermo Fisher Scientific, USA) at 37°C for 30 min. The loading solution was discarded, cells were washed three times with PBS (5 min per wash), and trypsinized for 3 min. Digestion was terminated by adding culture medium, cells were gently resuspended and counted, and the cell suspension was adjusted to 500 cells/μL to generate the donor astrocyte suspension. In the remaining wells of the 12‑well plate, culture medium was removed and cells were washed once with PBS, then 1 mL of the prepared donor astrocyte suspension was added to each well. After 2.5 h incubation in a CO_2_ incubator, images were acquired with Cytation C10 (BioTek, USA) and the fluorescence intensity of Calcein in receiver astrocytes surrounding a single donor astrocyte was quantified to assess gap junctional coupling.

**3.17. 2-NBDG uptake assay**

Astrocytes were seeded at a density of 2 × 10^4^ cells per well in a 96-well plate. A 2-NBDG (BioGems, USA) staining solution was prepared by dissolving it in PBS. Astrocytes were washed with PBS and then incubated with 50 μM 2-NBDG at 37 °C for 30 min. After incubation, the cells were washed with Hank's balanced salt solution (HBSS). The fluorescence intensity of 2-NBDG (E_x_/E_m_ = 465/540 nm) was measured using a microplate reader (Perkin Elmer, USA).

**3.18. Immunoprecipitation-mass spectrometry (IP-MS)**

Anti-Cx43 antibody (Cell Signaling Technology, 83649, USA) was used with Protein A+G magnetic beads (Beyotime, China) to isolate beads containing protein complexes. The beads were incubated in a reaction buffer (1% SDC/100 mM Tris-HCl, pH 8.5/10 mM TCEP/40 mM CAA) at 95 °C for 10 min to facilitate protein denaturation, cysteine reduction, and alkylation. The resulting eluates were diluted with an equal volume of H_2_O and subjected to overnight trypsin digestion at 37 °C using a trypsin: protein ratio of 1:50 (w/w). The digestion was terminated the following day by adjusting the pH to 6.0 with trifluoroacetic acid (TFA). After centrifugation at 12000 × g for 15 min, peptide purification was performed using self-prepared SDB desalting columns. The eluate was then vacuum dried and stored at −20 °C for subsequent analysis conducted using an UltiMate 3000 RSLCnano system coupled online with a Q Exactive HF mass spectrometer via a Nanospray Flex ion source (Thermo Fisher Scientific, USA). Peptide samples were introduced into a C18 Trap column (75 µm × 2 cm, 3 µm particle size, 100 Å pore size, Thermo Fisher Scientific, USA) and separated on an in-house packed reversed-phase C18 analytical column with ReproSil-Pur C18-AQ resin (75 µm × 25 cm, 1.9 µm particle size, 100 Å pore size). A separation gradient was established using mobile phases A (0.1% formic acid/3% DMSO/97% H_2_O) and B (0.1% formic acid/3% DMSO/97% ACN) at a 300 nL/min flow rate.

The MS analysis adopted the DDA top20 mode with a full scan range of 350–1500 m/z. The AGC Target value for the full MS scan was set at 3E6 charges, with a maximum injection time of 30 ms and a resolution of 60000 at m/z 200. Fragmentation was performed by high-energy collisional dissociation (HCD) with a normalized collision energy of 28. Fragment ion scans were recorded at a resolution of 15000, AGC of 1E5, and a maximum fill time of 50 ms. Dynamic exclusion was activated with a 30-second setting. Data analysis was performed using MaxQuant and the Andromeda database search algorithm against the mouse database by applying specific parameters for quantification and modification identification.

The acquired spectra were searched against a mouse database, considering variable modifications such as oxidation (M), acetylation (Protein N-term), and deamidation (NQ), along with fixed modifications, including carbamidomethylation (C). Trypsin/P was selected based on its digestion specificity. The MS1 match tolerance was set to 20 ppm for the initial search and 4.5 ppm for the subsequent search, whereas the MS2 tolerance was set to 20 ppm. The search results were filtered with a 1% FDR threshold at both the protein and peptide levels. Proteins identified as decoy hits, contaminants, or sites only were excluded from further analysis, and the remaining identifications were used for subsequent quantification assessments.

**3.19. Molecule docking and dynamics simulation**

ZDOCK 3.0.21 was employed to predict the binding modes of the Cx43 and IDH3α proteins [16]. Structures were refined using PyMOL 2.5.3. Docking investigations were performed using the default settings of ZDOCK 3.0.2, employing global rigid-body docking. After the docking phase, energy minimization was performed using AMBER18 in the ff14SB force field [17]. The resulting conformations of the protein complexes post-energy minimization were visualized and analyzed using PyMOL 2.5.3. Based on the protein-protein complexes generated from docking, the initial structures underwent all-atom molecular dynamics simulations using AMBER 18 software. Prior to simulations, the proteins were parameterized using an ff14SB protein force field [18]. Hydrogen atoms were introduced to the systems using the LEaP module, and a truncated octahedral TIP3P solvent box was positioned at a distance of 10 Å from the systems. Na^+^ and Cl^-^ ions were added to neutralize the charge of the system. The topological and parameter files required for the simulations are generated. Molecular dynamics simulations were carried out using AMBER 18 software. Throughout the simulations, a non-bonded cutoff of 10 Å was applied, the Particle Mesh Ewald (PME) method was used for long-range electrostatic calculations, the SHAKE algorithm was employed to constrain hydrogen bond lengths, and the Langevin algorithm was utilized for temperature control with a collision frequency (γ) set to 2 ps^-1^. The system pressure was maintained at 1 atm with an integration time step of 2 fs and trajectory snapshots were saved every 10 ps for subsequent analyses. The binding free energies between the receptors and ligands in all systems were calculated using the MM/GBSA method. In this study, MD trajectories of 45–50 ns were employed for computation using the following formula:

| $\text{ΔG}_{\text{bind}}\text{=}\text{ΔG}_{\text{complex}}\text{ }\text{– (}\text{ΔG}_{\text{receptor}}\text{+ }\text{ΔG}_{\text{ligand}}\text{)}$ |  |  |
| --- | --- | --- |
| $\text{ =}\text{ΔE}_{\text{internal}}\text{+}\text{ΔE}_{\text{VDW}}\text{+}\text{ΔE}_{\text{elec}}\text{+ΔG}_{\text{GB}}\text{+}\text{ΔG}_{\text{SA}}$ |  | (1) |

In formula (1), ΔE_internal_ represented the internal energy, ΔE_VDW_ represented van der Waals interactions, and ΔE_elec_ represented electrostatic interactions. The internal energy comprised bond, angle, and torsional energy; ΔG_GB_ and ΔG_GA_ collectively denoted solvation-free energy. Here, G_GB_ represents the polar solvation-free energy, and G_SA_ represents the nonpolar solvation-free energy. For ΔG_GB_, we employed the GB model developed by Nguyen et al. for the calculations (igb = 2) [19]. The nonpolar solvation free energy (ΔG_SA_) was determined based on the product of surface tension (γ) and the accessible surface area of the solvent (SA), calculated as ΔG_SA_ = 0.0072 × ΔSASA [20]. Entropy changes were excluded from this study owing to their high computational demands and low precision [20].

**3.20. Microscale thermophoresis**

Recombinant human Cx43 protein (MCE, USA) was conjugated with RED-NHS, following the manufacturer's protocol (NanoTemper, Germany). Post buffer exchange, protein purification, and equilibration, a 2 μM solution of the labeled protein was attained. All assessments were conducted utilizing the Monolith NT.115 apparatus (NanoTemper, Germany) at 25 °C, with assays conducted at 40% excitation power. The recombinant human IDH3α protein (Biodragon, China) was solubilized in PBS to create stock solutions. Throughout the MST experiments, IDH3α protein was progressively diluted 1:1 with PBS.

**3.21. Western blot**

Cultured cells and brain tissue were collected and lysed using a cell lysis buffer for Western blotting and IP (Beyotime, China), which contained a mixture of protease and phosphate inhibitors. Mitochondrial proteins were extracted using the cell mitochondria isolation kit (Beyotime, China). Cell membrane proteins were extracted using a membrane and cytosol protein extraction kit (Beyotime, China). Anti-TXNIP antibody (proteintech, 18243-1-AP, China), anti-Cx43 antibody (Cell Signaling Technology, 83649, USA), anti-c-Myc antibody (Transgen, HT101, China) were used with Protein A+G magnetic beads (Beyotime, China) to isolate beads containing protein complexes.

The proteins were then separated by electrophoresis on a 10% SDS-PAGE gel, with equal amounts of protein (10 μg) from the lysates, and transferred onto a PVDF membrane (Merck Millipore, USA). Following a 2-hour blocking with 5% bovine serum albumin (BSA) at room temperature, the membranes were incubated overnight at 4 ℃ with primary antibodies: anti-Cx43 (1:1000, Cell Signaling Technology, 83649, USA), anti-Calnexin (1:1000, ABclonal, A15631, China), anti-GAPDH (1:50000, ABclonal, A19056, China), anti-AMPK alpha 1+AMPK alpha 2 (1:1000, Abcam, ab131512, UK), anti-AMPK alpha 1 (phospho T183)+AMPK alpha 2 (phospho T172) (1:1000, Abcam, ab133448, UK), anti-GLUT1 (1:1000, Abcam, ab115730, UK), anti-TXNIP (1:1000, ABclonal, A9342, China), anti-IDH3α (1:1000, proteintech, 15909-1-AP, China), anti-VDAC1 (1:1000, Beyotime, AF1027, China), anti-Ubiquitin (1:1000, Cell Signaling Technology, 3936, USA), anti-Phospho-(Ser/Thr) Phe (1:1000, Abcam, ab300625, UK), anti-c-Myc (1:1000, Transgen, HT101, China), anti-DYKDDDDK (1:1000, Transgen, HT201, China), anti-Na-K ATPase (1:1000, Cell Signaling Technology, 3010, USA). and anti-β-actin (1:50000, ABclonal, AC026, China). After three washes, the blots were incubated with secondary antibodies against the corresponding species at room temperature for 2 h. Protein expression was measured using an ImageQuant LAS 4000 mini-detection system (GE Healthcare, USA). Gel analysis was performed using the ImageJ software.

**3.22. Immunohistochemistry and immunocytochemistry**

For immunohistochemical analysis, the mice were anesthetized and perfused with PBS. The brains were then immersed in 4% paraformaldehyde for 48 h, followed by dehydration in 30% sucrose in paraformaldehyde. Coronal sections of the brain, with a thickness of 40 μm, were obtained using a cryomicrotome (Leica, Germany). These sections were sequentially immersed in an antigen fixation solution for 15 min, 1% Triton X-100 for 10 min, and 5% BSA for 30 min. Subsequently, the sections were incubated overnight at 4 ℃ with the primary antibodies: anti-GFAP at a 1:200 dilution (Dako, Z0334, Danmark), anti-Iba1 at a 1:100 dilution (Abcam, ab289874, UK), anti-Cx43 at a 1:200 dilution (Thermo Fisher Scientific, 13-8300, USA) and anti-IDH3α at a 1:200 dilution (proteintech, 15909-1-AP, China). The following day, the corresponding secondary antibodies (Thermo Fisher Scientific, USA) were added, and the cells were incubated in the dark at room temperature for 2 h. After a 5-minute wash in PBS, the samples were mounted with DAPI Fluoromount-G (Thermo Fisher Scientific, USA) under protection from light.

For immunocytochemical analysis, the cells were fixed with 4% paraformaldehyde for 20 min, washed with PBS, and treated with 0.3% Triton X-100 for 5 min to permeabilize the cell membrane. Subsequently, cells were blocked with 5% BSA at room temperature for 1 hour. Next, the cells were incubated overnight at 4 °C with the primary antibodies: anti-GFAP (1:200, Dako, Z0334, Danmark), anti-MAP2 (1:200, Cell Signaling Technology, 4542, USA), anti-GLUT1 (1:200, Abcam, ab115730, UK) and anti-EAAT2 (1:200, Abcam, ab313454, UK). The following day, the corresponding secondary antibodies (Thermo Fisher Scientific, USA) were added, and the cells were incubated in the dark at room temperature for 1 hour. After a 5-minute wash in PBS, the samples were mounted with DAPI Fluoromount-G (Thermo Fisher Scientific, USA) under protection from light. For phalloidin staining, the cells were incubated with phalloidin (1:250, Thermo Fisher Scientific, A12379, USA) at room temperature for 20 min, followed by incubation with a secondary antibody. For wheat germ agglutinin (WGA) staining, the cells were incubated with WGA (1:300, Thermo Fisher Scientific, W32466, USA) at room temperature for 25 min, followed by incubation with a secondary antibody. After washing with PBS, cells were mounted for imaging. The mounted samples were observed under a confocal laser-scanning microscope (Cytation C10, BioTek, USA). The complexity of branching in astrocytes and neurons was quantitatively assessed using the Sholl analysis tool (ImageJ), which measured the intersections of cellular processes with concentric circles centered around the cell body.

**3.23. Brain slice cultures and two-photon microscopy**

Thy1-GFP mice subjected to CSDS were anesthetized using isoflurane, following which their brains were rapidly removed and transferred into ice-cold ACSF. Coronal sections (200 µm) including the mPFC were prepared in ice-cold ACSF employing a vibratome (Leica, Germany) and subsequently moved to an incubation chamber containing ACSF maintained at 32 °C for 1 hour. The brain slices were then immersed in solution containing ACM and 10 mM sodium lactate, where they were maintained at 32 °C for 6 hours. All solution was continuously saturated with a gas mixture of 95% O_2_ and 5% CO_2_ (vol/vol). Upon completion of the incubation, the brain slices were fixed in 4% paraformaldehyde and subsequently mounted for observation using an upright multiphoton laser-scanning microscope (FluoView FVMPE-RS; Olympus, Japan). Two-photon excitation was achieved using MAITAI eHPDS-OL and Spectra Physics InSight DS-OL lasers (Mai Tai; Spectra-Physics, USA). The area of GFP fluorescence in the dendritic terminals was analyzed using ImageJ software.

**3.24. Imaging flow cytometry**

The mitochondrial membrane potential of neurons was evaluated using the JC-1 mitochondrial membrane potential assay kit (Beyotime, China) according to the manufacturer's instructions. Before flow cytometry analysis, the cells were washed and suspended in 50 µL of cold PBS containing 2% FBS. Subsequently, the cells were analyzed using an ImageStreamX system flow cytometer (Cytek, USA). Data analysis was performed using IDEAS 6.2 software (Merck Millipore, USA).

**4. References**

[1] Yao S, Xu MD, Wang Y, Zhao ST, Wang J, Chen GF, et al. Astrocytic lactate dehydrogenase A regulates neuronal excitability and depressive-like behaviors through lactate homeostasis in mice. Nat Commun. 2023;14:729.

[2] Liu MY, Yin CY, Zhu LJ, Zhu XH, Xu C, Luo CX, et al. Sucrose preference test for measurement of stress-induced anhedonia in mice. Nat Protoc. 2018;13:1686-98.

[3] Song XW, Dong ZY, Long XY, Li SF, Zuo XN, Zhu CZ, et al. REST: a toolkit for resting-state functional magnetic resonance imaging data processing. PLoS One. 2011;6:e25031.

[4] Yan CG, Wang XD, Zuo XN, Zang YF. DPABI: Data Processing & Analysis for (Resting-State) Brain Imaging. Neuroinformatics. 2016;14:339-51.

[5] Wang J, Wang X, Xia M, Liao X, Evans A, He Y. GRETNA: a graph theoretical network analysis toolbox for imaging connectomics. Front Hum Neurosci. 2015;9:386.

[6] Krzywinski M, Schein J, Birol I, Connors J, Gascoyne R, Horsman D, et al. Circos: an information aesthetic for comparative genomics. Genome Res. 2009;19:1639-45.

[7] Xia MR, Wang JH, He Y. BrainNet Viewer: A Network Visualization Tool for Human Brain Connectomics. PLos One. 2013;8:e68910.

[8] Pan RY, He L, Zhang J, Liu XH, Liao YJ, Gao J, et al. Positive feedback regulation of microglial glucose metabolism by histone H4 lysine 12 lactylation in Alzheimer's disease. Cell Metab. 2022;34:634-48.

[9] Wieckowski MR, Giorgi C, Lebiedzinska M, Duszynski J, Pinton P. Isolation of mitochondria-associated membranes and mitochondria from animal tissues and cells. Nat Protoc. 2009;4:1582-90.

[10] Beahm DL, Oshima A, Gaietta GM, Hand GM, Smock AE, Zucker SN, et al. Mutation of a conserved threonine in the third transmembrane helix of α- and β-connexins creates a dominant-negative closed gap junction channel. J Biol Chem. 2006;281:7994-8009.

[11] Lu G, Jiang SJ, Ashraf M, Haider KH. Subcellular preconditioning of stem cells: mito-Cx43 gene targeting is cytoprotective via shift of mitochondrial Bak and Bcl-xL balance. Regen Med. 2012;7:323-34.

[12] Zhang H, Zheng Q, Guo T, Zhang S, Zheng S, Wang R, et al. Metabolic reprogramming in astrocytes results in neuronal dysfunction in intellectual disability. Mol Psychiatry. 2024;29:1569-82.

[13] Millard P, Delepine B, Guionnet M, Heuillet M, Bellvert F, Letisse F. IsoCor: isotope correction for high-resolution MS labeling experiments. Bioinformatics. 2019;35:4484-7.

[14] Picoli C, Soleilhac E, Journet A, Barette C, Comte M, Giaume C, et al. High-Content Screening Identifies New Inhibitors of Connexin 43 Gap Junctions. Assay Drug Dev Techn. 2019;17:240-8.

[15] Gingrich J, Pu Y, Veiga-Lopez A. A modified parachute assay for assessment of gap junction intercellular communication in placental trophoblast cells. Toxicol Mech Methods. 2021;31:393-9.

[16] Pierce BG, Hourai Y, Weng Z. Accelerating protein docking in ZDOCK using an advanced 3D convolution library. PLoS One. 2011;6:e24657.

[17] Salomon-Ferrer R, Case DA, Walker RC. An overview of the Amber biomolecular simulation package. Wires Comput Mol Sci. 2013;3:198-210.

[18] Maier JA, Martinez C, Kasavajhala K, Wickstrom L, Hauser KE, Simmerling C. ff14SB: Improving the Accuracy of Protein Side Chain and Backbone Parameters from ff99SB. J Chem Theory Comput. 2015;11:3696-713.

[19] Nguyen H, Roe DR, Simmerling C. Improved Generalized Born Solvent Model Parameters for Protein Simulations. J Chem Theory Comput. 2013;9:2020-34.

[20] Weiser J, Shenkin PS, Still WC. Approximate atomic surfaces from linear combinations of pairwise overlaps (LCPO). J Comput Chem. 1999;20:217-30.
