# Supplementary material for: Noncanonical role of astrocytic mitochondrial Cx43: suppressing IDH3α to sustain glycolytic homeostasis against depression
Source: Cell Death Dis. 2025 Dec 8;17(1):94. doi: 10.1038/s41419-025-08309-1 (PMC12830930; doi:10.1038/s41419-025-08309-1)

Fig. 2A

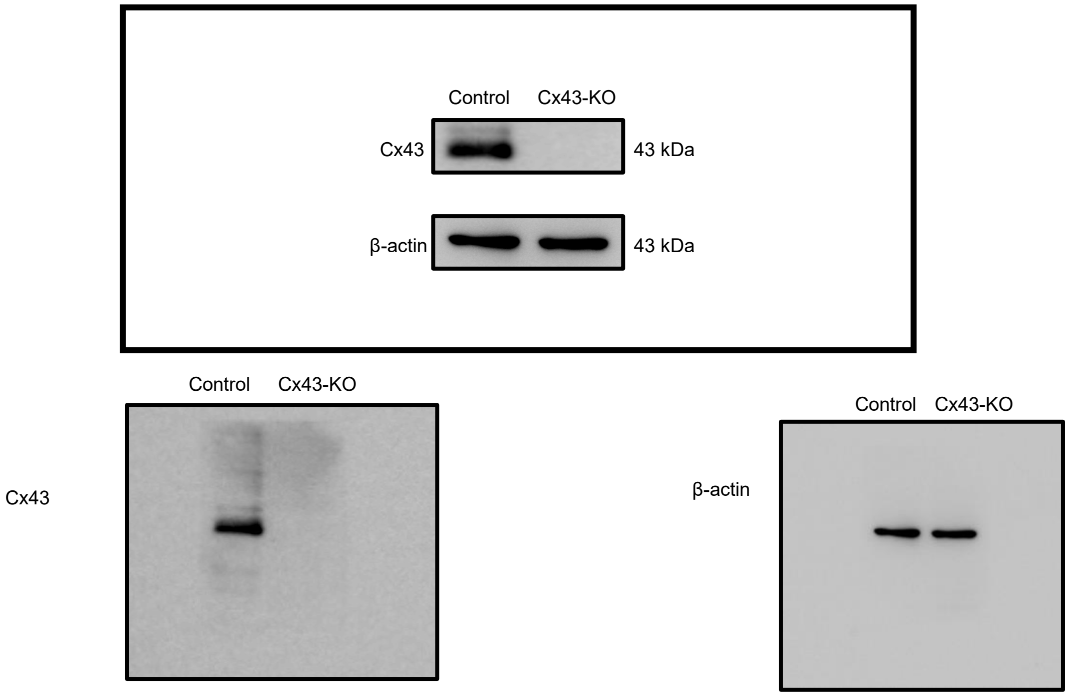
Control
Cx43-KO
Cx43
43 kDa
β-actin
43 kDa
Control
Cx43-KO
Cx43
Control
Cx43-KO
β-actin

Fig. 5D

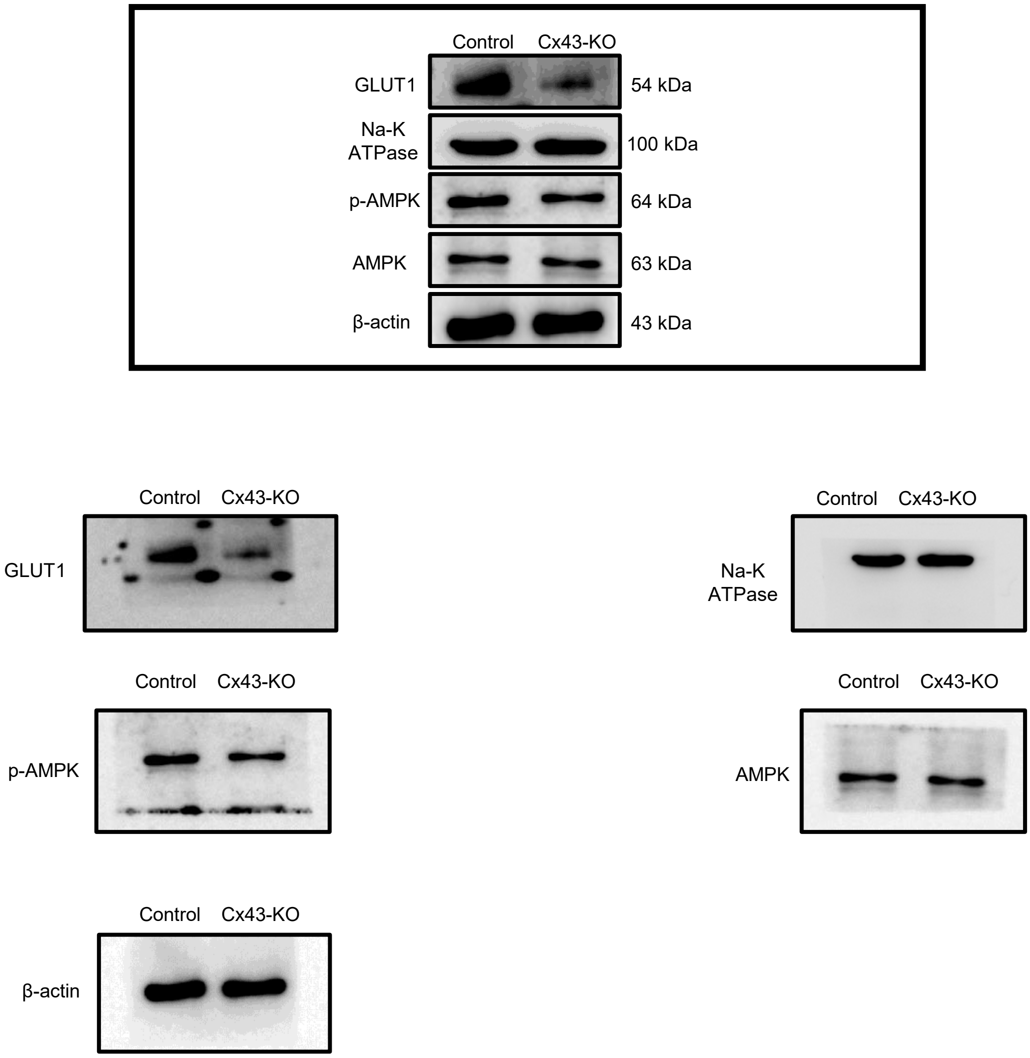
Control
Cx43-KO
GLUT1
54 kDa
Na-K ATPase
100 kDa
p-AMPK
64 kDa
AMPK
63 kDa
β-actin
43 kDa
Control
Cx43-KO
GLUT1
Control
Cx43-KO
Na-K ATPase
Control
Cx43-KO
p-AMPK
Control
Cx43-KO
AMPK
Control
Cx43-KO
β-actin

Fig. 5H

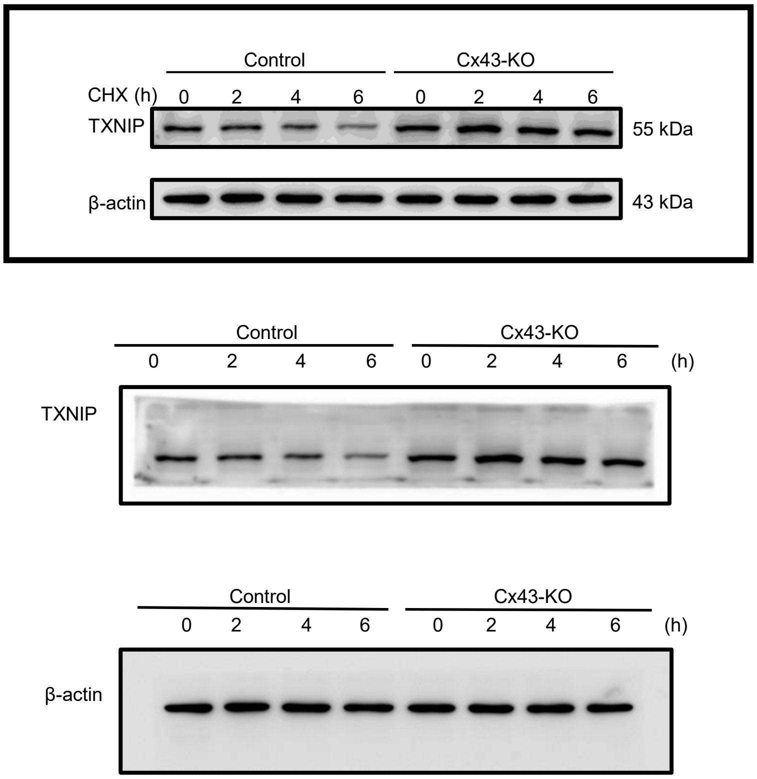

Control
Cx43-KO
CHX (h)
0 2 4 6 0 2 4 6
TXNIP
55 kDa
β-actin
43 kDa
Control
Cx43-KO
0 2 4 6 0 2 4 6 (h)
TXNIP
Control
Cx43-KO
0 2 4 6 0 2 4 6 (h)
β-actin

Fig. 5Q

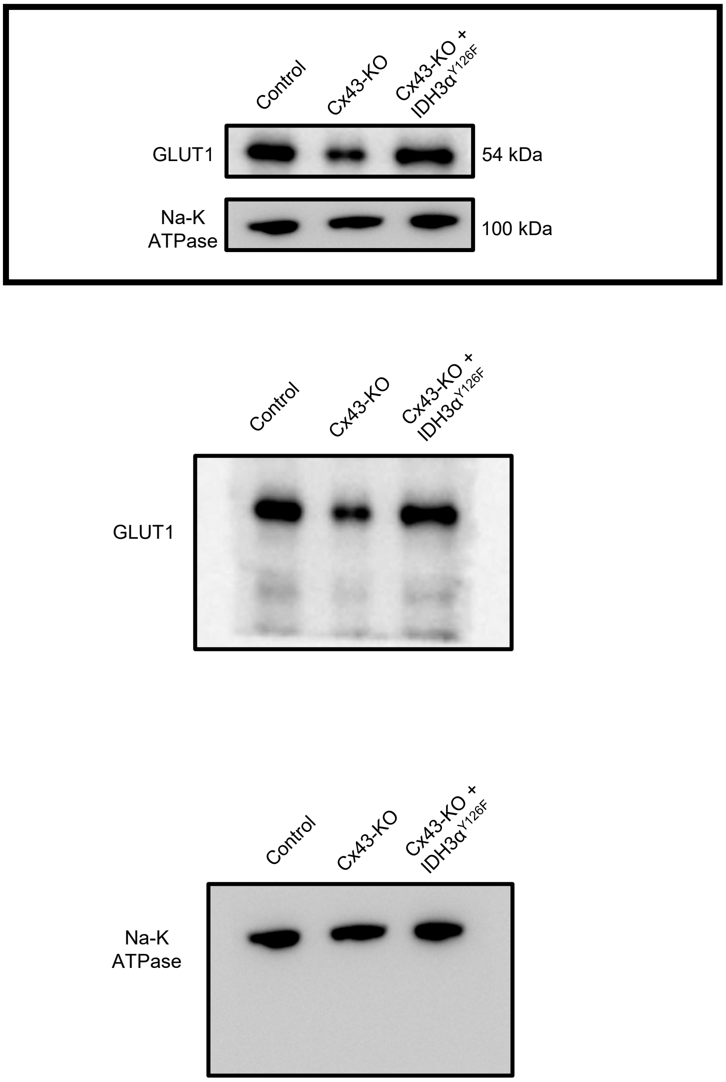

Control
Cx43-KO
Cx43-KO + IDH3α Y126F
GLUT1
54 kDa
Na-K ATPase
100 kDa
Control
Cx43-KO
Cx43-KO + IDH3α Y126F
GLUT1
Control
Cx43-KO
Cx43-KO + IDH3α Y126F
Na-K ATPase

Supplementary Fig. S1D

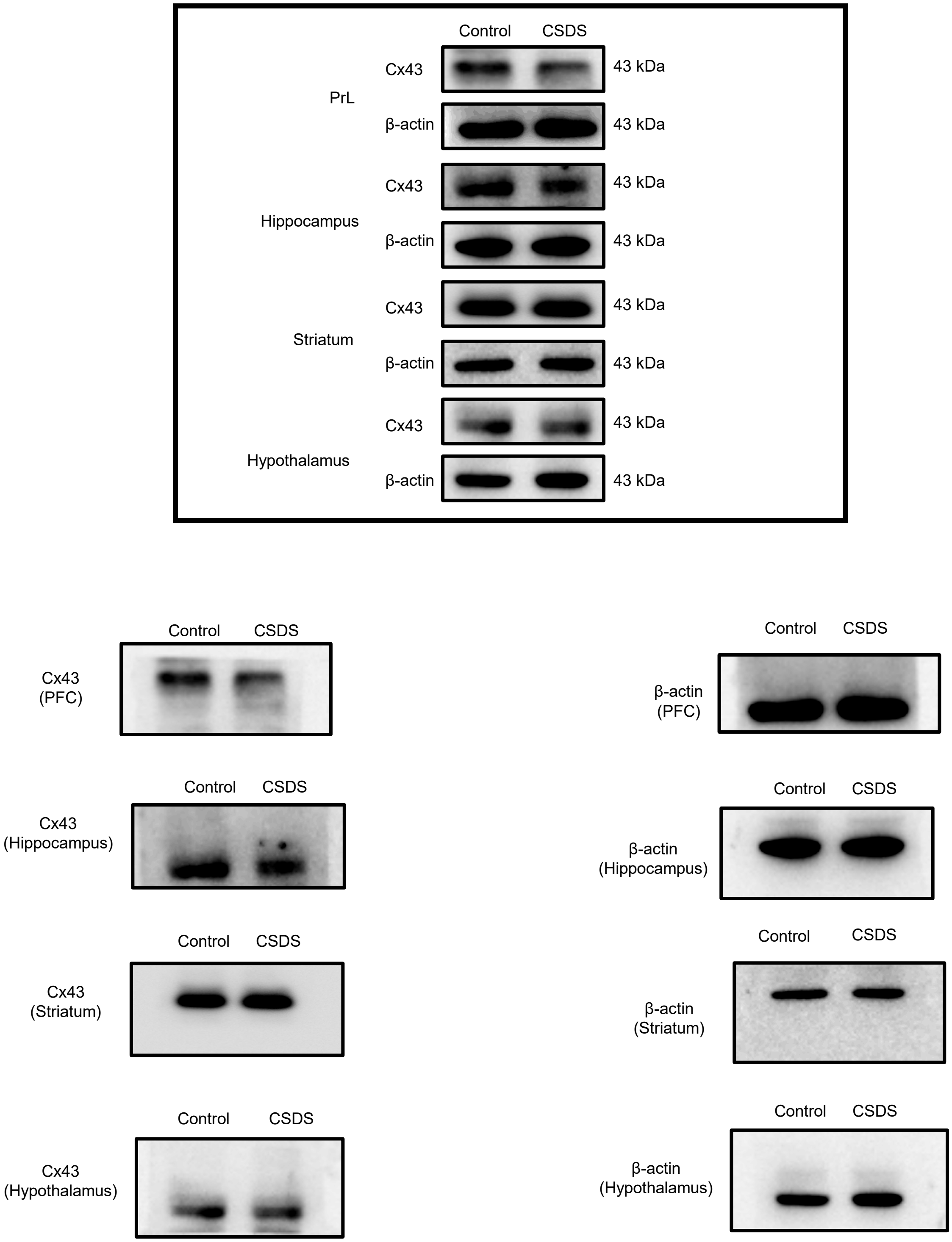

Supplementary Fig. S4A

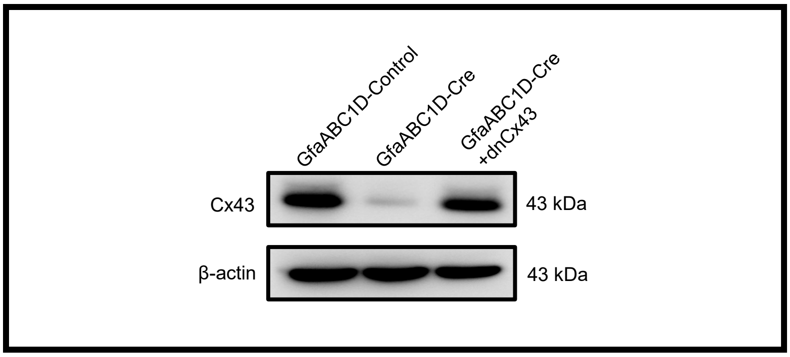

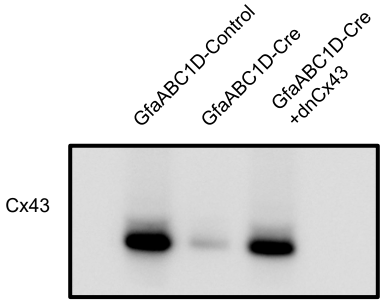

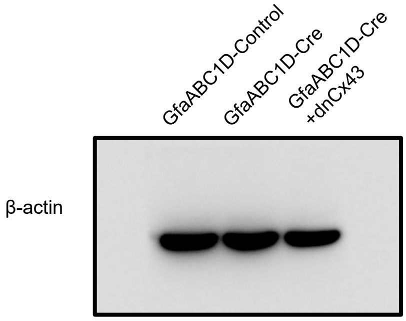

Supplementary Fig. S4D

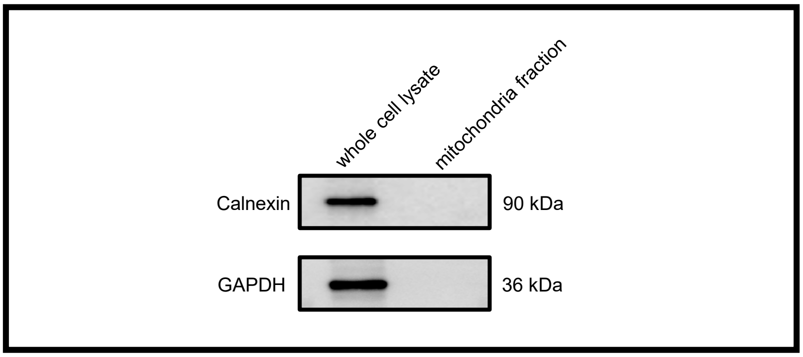

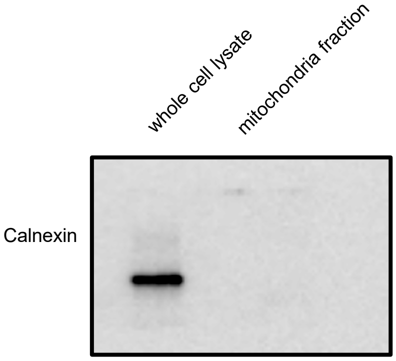

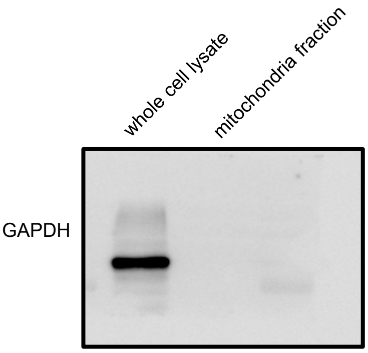

Supplementary Fig. S4E

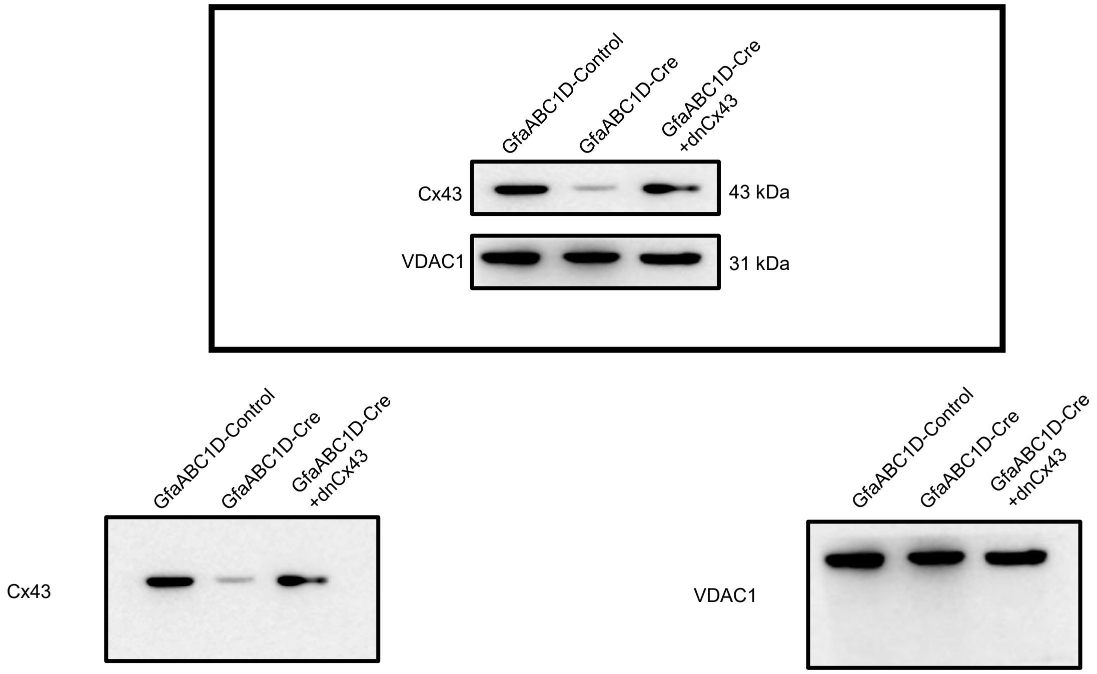

Supplementary Fig. S6H

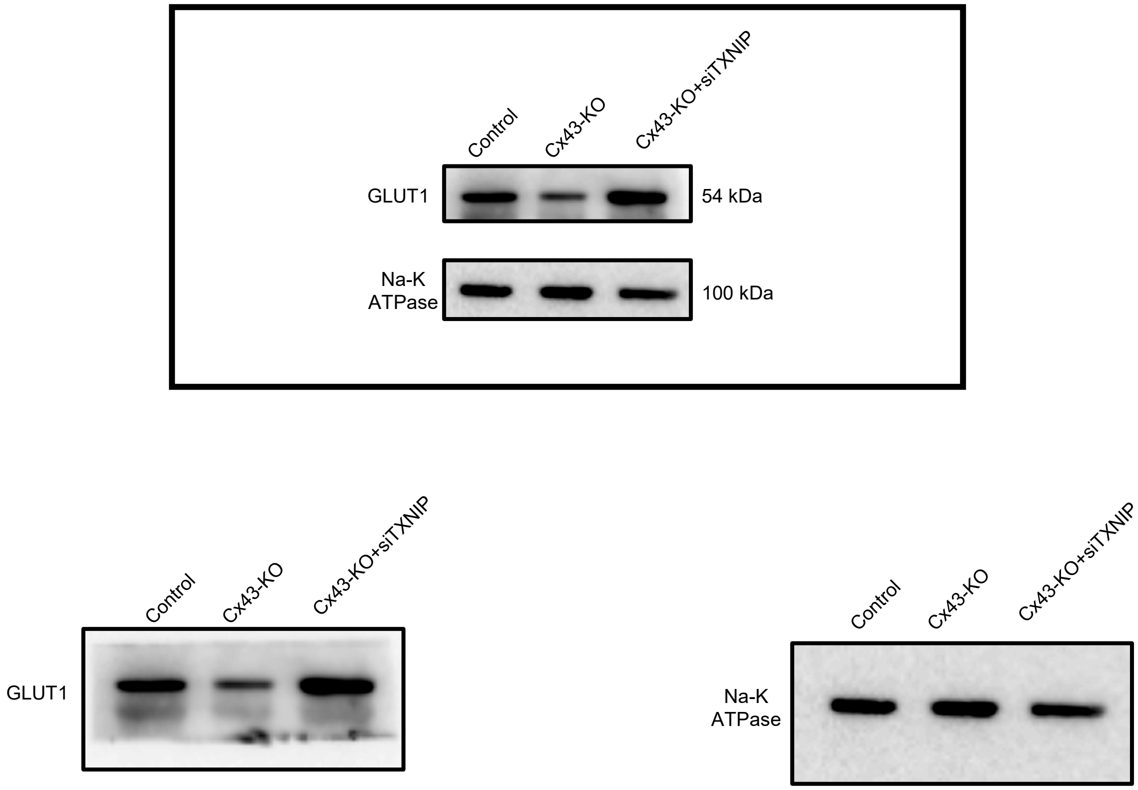

Supplementary Fig. S6M

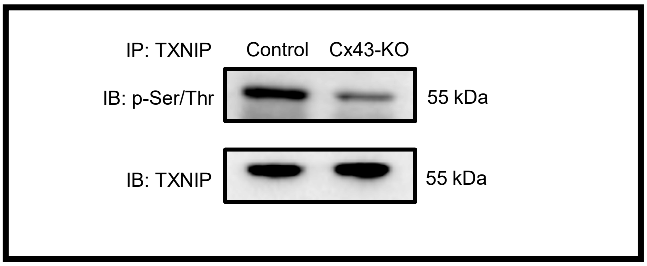

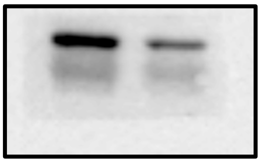

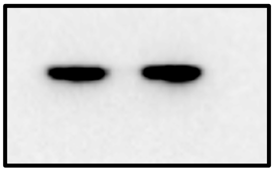

Supplementary Fig. S6O

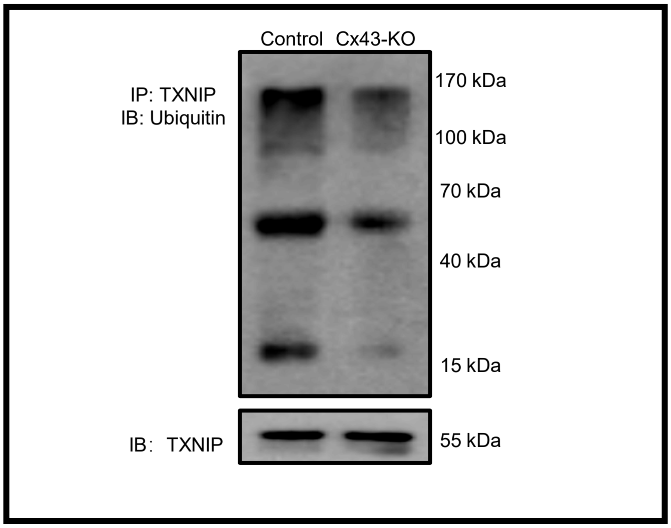

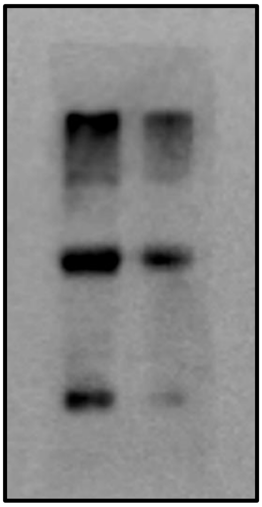

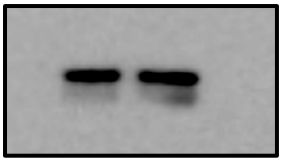

Supplementary Fig. S7G

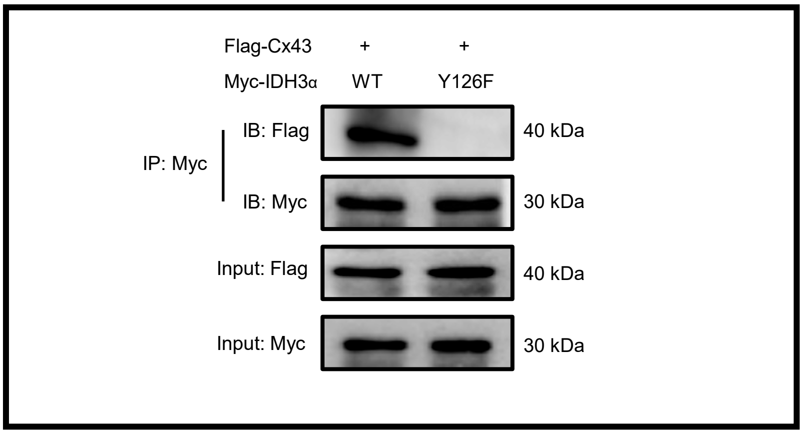

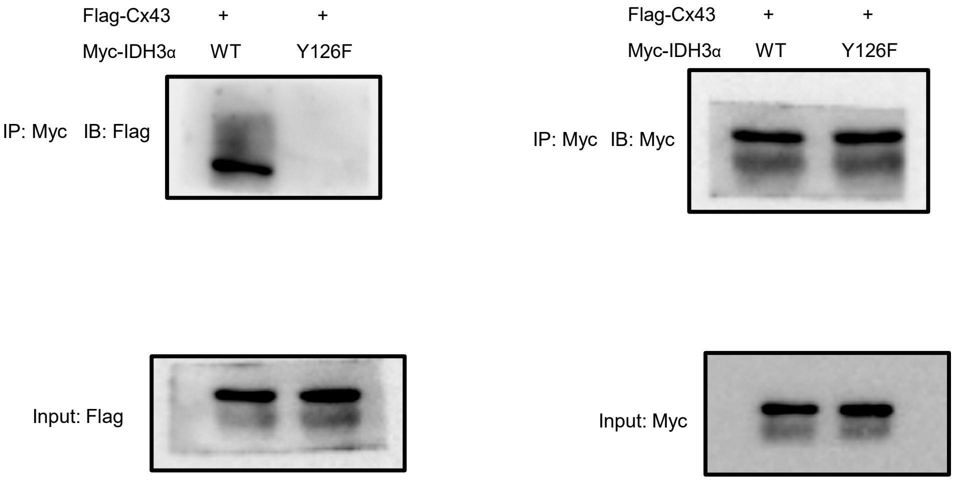

Supplement: Supplementary file 2 — Original blots [file 41419_2025_8309_MOESM2_ESM.pdf]
